# Reductive Cyclopropanation through Bismuth Photocatalysis

**DOI:** 10.1021/jacs.4c07262

**Published:** 2024-08-05

**Authors:** Shengyang Ni, Davide Spinnato, Josep Cornella

**Affiliations:** Max-Planck-Institut für Kohlenforschung, Kaiser-Wilhelm-Platz 1, Mülheim an der Ruhr, 45470, Germany

## Abstract

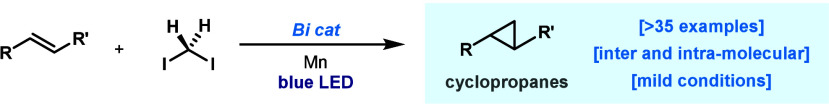

We present here a catalytic method based on a low-valent Bi complex capable of cyclopropanation of double bonds under blue LED irradiation. The catalysis features various unusual Bi-based organometallic steps, namely, (1) two-electron inner sphere oxidative addition of Bi(I) complex to CH_2_I_2_, (2) light-induced homolysis of the Bi(III)–CH_2_I bond, (3) subsequent iodine abstraction-ring-closing, and (4) reduction of Bi(III) to Bi(I) with an external reducing agent to close the cycle. Stoichiometric organometallic experiments support the proposed mechanism. This protocol represents a unique example of a reductive photocatalytic process based on low-valent bismuth radical catalysis.

The cyclopropane motif remains one of the most recognizable structures in organic chemistry, present in molecules with relevant medicinal and agrochemical applications.^1^ Construction of these motifs has long benefited from the [2 + 1] transformation, capitalizing on the broad availability of olefin precursors and carbene^2^ or carbenoid sources (Figure 1A).^3^ Since the venerable Simmons–Smith reaction using Zn(Cu) and diiodomethane,^4^ a plethora of variants have appeared in the literature using various elements of the periodic table.^5^ The importance of this moiety is also manifested in the myriad of examples where the canonical [2 + 1] disconnection expands beyond the simple two-electron olefin/carbene combination.^6^ In recent years, novel photocatalytic methods have appeared enabling one-electron cyclopropanation reactions, with or without the aid of transition metals.^7^ Despite the continuous efforts,^5−7^ examples of main-group catalysts performing one-electron redox processes,^8^ and in particular, cyclopropanation reactions, are largely underexplored. Our group has recently reported the rich reactivity of *N,C,N*-pincer bismuthinidenes in one- and two-electron oxidative addition processes.^9^ The resulting Bi(III) complexes are characterized by having weak Bi–X bonds, which succumb to homolysis upon thermal or photochemical stimuli. We have recently capitalized on this weak bonding situation to develop C–N amination reactions^9a^ and a radical trifluoromethylation process under light irradiation.^9b^ In order to expand the horizon of opportunities for Bi as a redox catalyst, we decided to interrogate its potential in C–C bond forming radical reductive couplings, a reactivity paradigm that blossomed for first-row transition metals.^10^ To this end, we provide here an *unprecedented reductive C–C coupling catalyzed by Bi* toward cyclopropanation, using alkyl diiodides and olefins (Figure 1B). The process is characterized by the ability of Bi to concatenate various mechanistic steps, through open-shell intermediates.^11^ Cyclopropanation is achieved through a series of (1) S_N_2-type reaction to C(sp^3^)–I;^12^ (2) homolysis of the Bi–C bond upon irradiation;^9a,9b^ (3) radical ring-closing forming Bi(III)–I_2_; and (4) recovery of the Bi(I) species through two-electron reduction. All proposed steps are supported via stoichiometric experiments, providing a blueprint of the putative working hypothesis.

**Figure 1:**
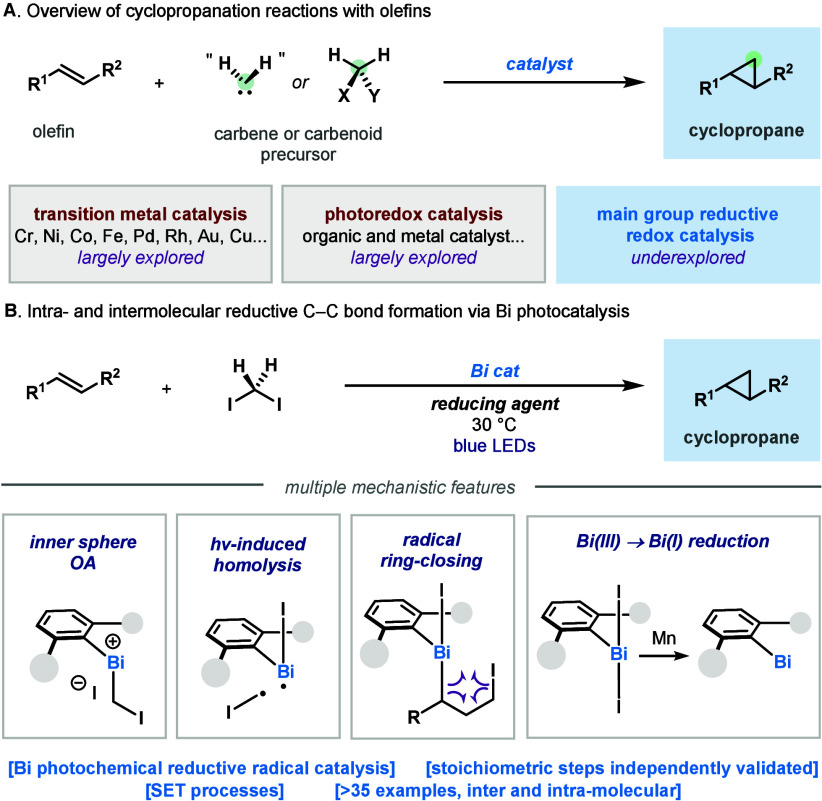
(A) Canonical [2 + 1] cyclopropane transformation: olefins and carbenes/carbenoids as precursors. (B) Expanding the palette of opportunities for bismuthinidenes: radical cyclopropanation with unique mechanistic features in Bi catalysis.

When chalcone (**1**) was mixed with diiodomethane (**2**) in the presence of 10 mol % of bismuthinidene **Bi-1** and 2.0 equiv of Mn in DMA, and irradiated at 465 nm at 30 °C, 76% of the cyclopropanated product **3** was obtained (Table 1, entry 1). The reaction in the absence of blue light or a Bi catalyst does not proceed (entries 2 and 3). The use of red light in place of blue light also resulted in no conversion to **3** (entry 4).^9c^ Replacing DMA with MeCN led to a slightly decreased yield of **3** (entry 5). Substituting Mn with Zn also resulted in a dramatically reduced reactivity toward **3**, possibly due to the weaker reducing capacity (entry 6). Interestingly, when the reaction was carried in an electrochemical cell to replace the bulk insoluble reductant by a Zn(s) cathode, a satisfactory 55% yield of **3** was obtained, thus opening the door to photoelectrochemical processes using Bi catalysis (entry 7).^13^ Interestingly, the use of the **Bi-1·[Cl**_**2**_**]** precursor also led to a significant 68% yield of **3**, thus highlighting the possibility of accessing Bi(I) *in situ* from an air-stable precursor (entry 8). Finally, product **3** could also be obtained starting from CH_2_Br_2_ and NaI, albeit in lower yields, likely due to the slower reaction of **Bi-1** with CH_2_Br_2_ compared to that with CH_2_I_2_ (entry 9).

**Table 1:**
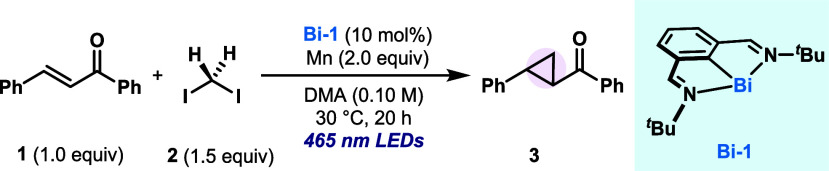
Optimization of the Bi-Catalyzed Reductive Cyclopropanation with Light^a^

entry	deviations from above^a^	yield of **3** (%)^b^
1	none	74 (70)^c^
2	without catalyst	trace
3	without light	trace
4	red LEDs	trace
5	MeCN instead of DMA	51
6	Zn instead of Mn	30
7	(−) Ni foam/(+) Zn; 5.0 mA, 465 nm LEDs; 12 h	55
8	air stable **Bi-1**·[Cl_2_] instead of Bi-1	68
9	CH_2_Br_2_/NaI instead of CH_2_I_2_	40

a Reaction conditions: **1** (1.0 equiv, 0.10 mmol) and **2** (1.5 equiv) in the presence of bismuthinidene **1** (10 mol %) under 465 nm LEDs irradiation at 30 °C for 20 h under argon atmosphere.

b Yields were determined by ^1^H NMR with 1,3,5-trimethoxybenzene as the internal standard.

c Isolated yield.

With an optimized protocol in hand, the scope of the alkene was studied (Table 2). In addition to **3**, chalcone derivatives bearing electron-donating (**4** and **5**) and electron-withdrawing groups (**6** and **7**) could be tolerated. Simple enones (**8**) or acrylates (**9**) were also amenable to the cyclopropanation reaction. Particularly, unprotected phenols were well tolerated, affording good yields of **10** and **11**. Interestingly, the use of deuterated diiodomethane-*d*_2_ resulted in complete retention of the deuterium percentage, thus giving access to interesting isotopically labeled compounds **12**. The radical cyclopropanation could be extended to β-styrenes simply by changing the solvent from DMA to MeCN (**13**–**23**). Amines (**15**), esters (**16**), acetals (**17**), biaryls (**18**), thioethers (**19**), halides (**20**), ethers (**13**, **14**, **17**) and heterocyclic motifs (**21**) were included in the scope and well tolerated under the optimized conditions. Compound **22** is the result of a cyclopropanation into a vinyl cyclopropane, which leads to stimulation of double-strained carbocyclic structures. The reaction was successfully scaled-up to 1.0 mmol for **13**, obtaining a satisfactory 77% isolated yield while reducing the catalyst loading to 1 mol %. Interestingly, the *E*/*Z* configuration of the alkene in **13** did not affect the stereoselectivity of the product: both *E*- and *Z*-anethole yielded the *trans*-product in d.r > 20:1. Remarkably, a trisubstituted alkene can also be converted into the desired product **23** in 46% yield, with a 9:1 diastereoselectivity, favoring the *trans* isomer. However, aliphatic and terminal alkenes, as well as tetrasubstituted styrenes, are currently beyond the scope (see Supporting Information (SI) for details).

**Table 2:**
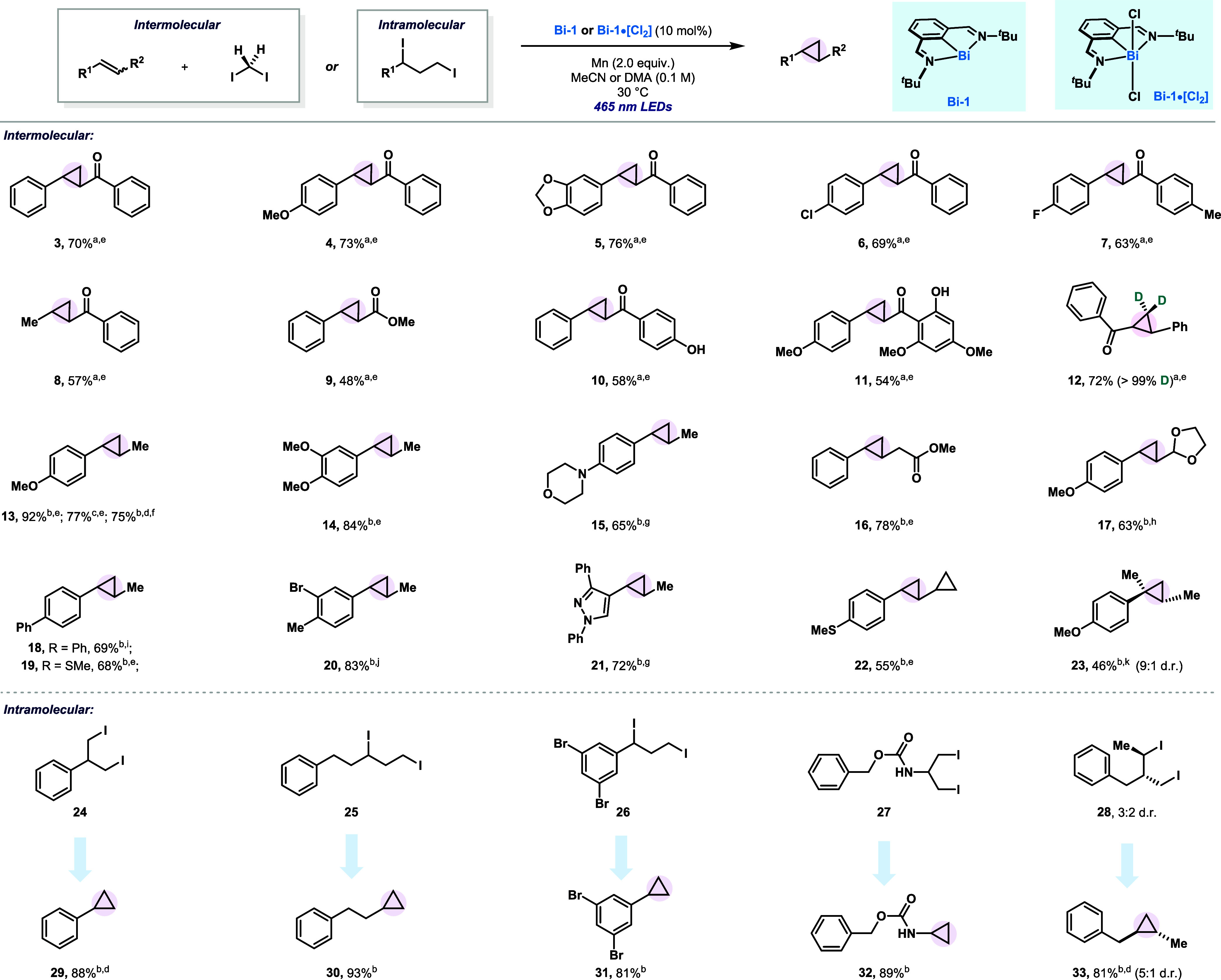
Scope of the Bi-Catalyzed Reductive Cyclopropanation with Light

a Conditions: **Bi-1** (10 mol %), Mn (2.0 equiv), DMA (0.10 M), 24 h, 465 nm LEDs.

b Conditions: **Bi-1·[Cl**_**2**_**]** (10 mol %), Mn (2.0 equiv), MeCN (0.10 M), 24 h, 465 nm LEDs.

c Conditions: 1.0 mmol scale, using **Bi-1·[Cl**_**2**_**]** (1 mol %), 24 h, 465 nm LEDs.

d ^1^H NMR yield using 1,3,5-trimethoxybenzene as the internal standard.

e From *E*/*Z* alkene >20:1.

f From *Z*/*E* alkene >20:1.

g From *E*/*Z* alkene = 1:4.

h From *E*/*Z* alkene = 1.8:1.

i From *E*/*Z* alkene = 1:3.

j From *E*/*Z* alkene = 3:5.

k From *E*/*Z* alkene = 1:1. All the products possessing 2 stereocenters have a *trans* configuration. Unless otherwise specified, the substrates have a d.r. > 20:1 (d.r.: diastereomeric ratio). Yields of isolated product are indicated in each case.

Having established the generality of intermolecular radical cyclopropanation, we were interested in applying this reactivity in an intramolecular fashion. For this reason, we focused on the use of 1,3-diiodoalkyl substrates, which could potentially afford the corresponding cyclopropanes. As depicted in Table 2, substituted 1,3-diiodoalkanes derived from the 1,3-diols could undergo ring-closing cyclopropanation with Bi under light in a reductive fashion. From simple aromatic (**29**) and aliphatic groups (**30**, **33**) to the presence of bromides (**31**) and carbamates (**32**), all were successfully accommodated, affording cyclopropanes in high yields. In line with previous work,^6d^ a stereoconvergent cyclization was conducted using substrate **28** (d.r. 3:2). Indeed, 1,2-disubstituted cyclopropane **33** could be formed in 81% yield with a dr of 5:1. Substrates with two secondary 1,3-diiodalkyl groups remain outside the scope of the transformation, likely due to the slower rate for OA with Bi(I).^9a,12b^

In order to shed light on the potential mechanistic steps behind the reactivity observed, we initially reacted **Bi-1** with diiodomethane. When mixing **Bi-1** (1.0 equiv) with **2** (5.0 equiv), a 77% yield of complex **34** could be isolated (Figure 2A, top). Formation of **34** was confirmed by HRMS and NMR, which enabled complete characterization in solution. Similarly, when **Bi-1** was treated with 1,3-diiodoalkane **24**, quantitative formation of **36** was obtained (Figure 2A, bottom). Again, complete characterization in solution was obtained via NMR spectroscopy (see SI). When **34** was reacted with β-styrene **35** under light irradiation in the absence of a reductant, 79% of **13** was observed with concomitant formation of **Bi-1·[I**_**2**_**]**. In the same vein, when **36** was irradiated also in the absence of a reducing agent, complete conversion to **29** was obtained (Figure 2A). Importantly, none of the reactions proceeded in the absence of 465 nm light, supporting the important role of the light in the homolytic process. This was further evidenced by stoichiometric light ON/OFF experiments (see SI). Additionally, when compound **34** was reacted with radical-stabilized TEMPO under 465 nm light, new adduct **37** was obtained together with **Bi-1·[I**_**2**_**]**, thus providing additional evidence of the radical process (Figure 2B). Finally, the role of the reducing reagent was also studied. When mixing both Bi(III) precursors **Bi-1·[I**_**2**_**]** and **Bi-1·[Cl**_**2**_**]** with an excess of insoluble Mn, complete conversion to **Bi-1** was obtained in the dark, thus suggesting that the Bi(III) → Bi(I) process can successfully proceed in the absence of light (Figure 2C).

**Figure 2:**
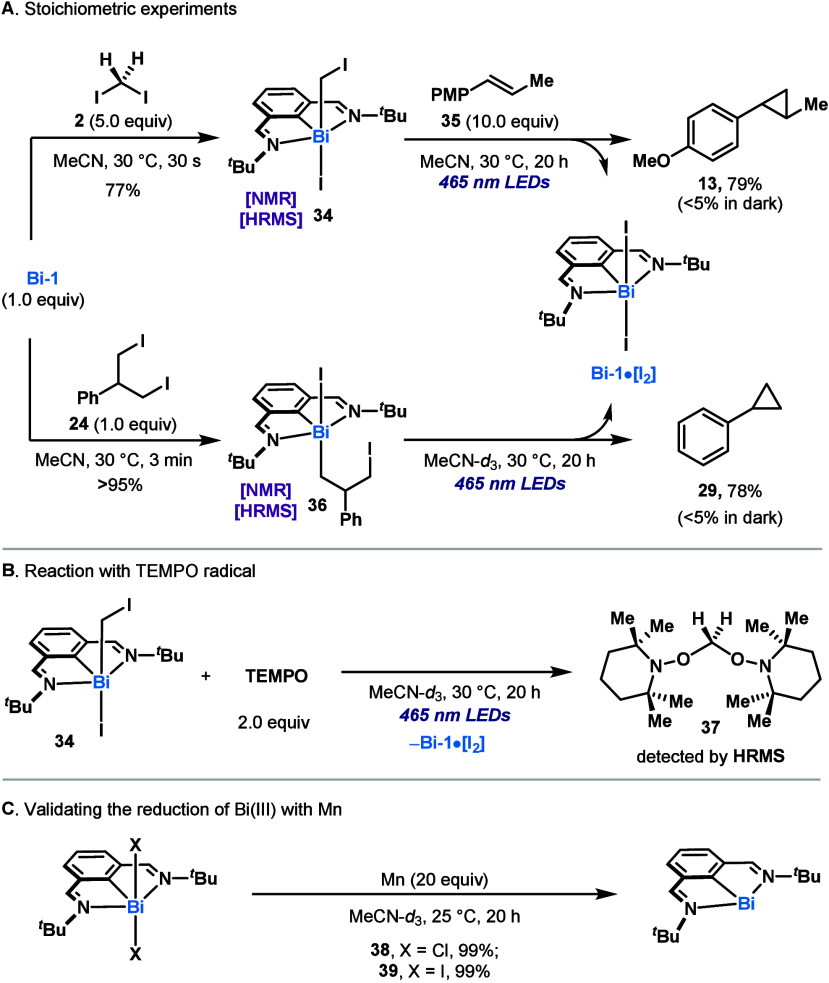
(A) Stoichiometric and radical trapping experiments; (B) Trapping alkyl radicals with TEMPO; (C) Reduction of Bi(III).

Based on these stoichiometric experiments, we tentatively proposed the catalytic cycle depicted in Figure 3. Initially, low-valent **Bi-1** (**I**) engages in an S_N_2-type oxidative addition with diiodomethane **2**,^12^ leading to intermediate **II**. Based on precedents from our group^9^ and the results in Figure 2A, these species can undergo bond-homolysis, presumably leading to fleeting species **III**, which rapidly engage in a radical addition to the olefin partner. This addition leads to **IV**, which upon ring-closing would afford **V**. We believe that the formation of Bi–I bonds (HSAB) provides a large driving force for the ring-closure and formation of **V**.^9c^ At this point, **V** can be reduced by the external reducing agent, thus recovering the propagating species **I**.

**Figure 3:**
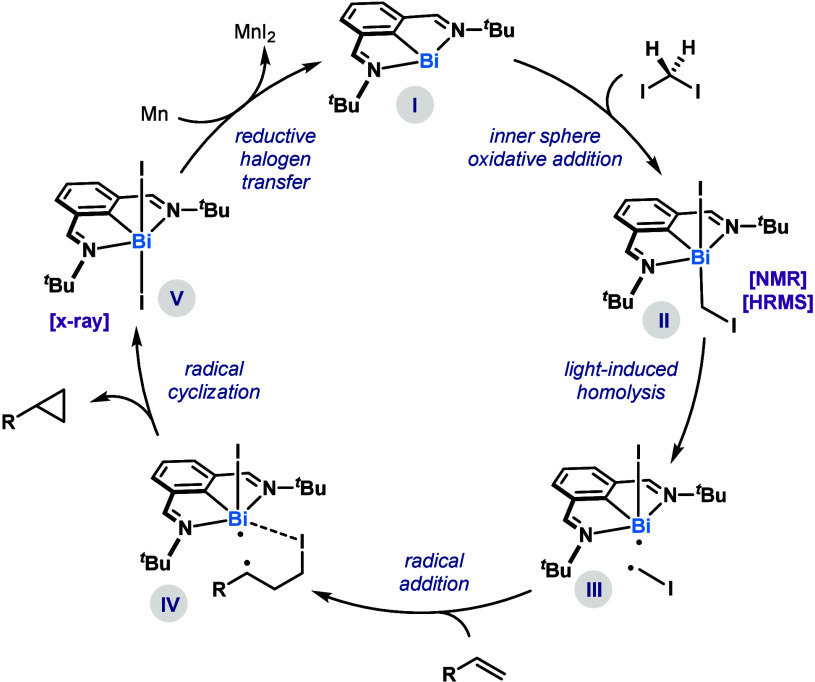
Working hypothesis for the Bi phototo-catalytic reductive cyclopropanation of olefins.

In summary, we disclose a photocatalytic radical reductive C–C bond formation reaction catalyzed by Bi that forges cyclopropanes. The reaction proceeds in both an intermolecular fashion between olefins and 1,1-diiodomethane and intramolecularly from the parent 1,3-diiodoalkanes. The process proceeds at room temperature and accommodates a variety of functional groups. Mechanistic experiments validate the role of light in promoting a homolytic cleavage of a Bi–C bond. Moreover, we were able to provide insight into a putative open-shell Bi(I)/Bi(II)/Bi(III)/Bi(I) redox catalytic cycle, which is uncommon for the main group elements. The combination with Mn as a reductant and light also provides a novel avenue which has not been reported either in bismuth catalysis or in other main group catalysis. We believe that these novel mechanistic features will aid the development of Bi-based radical chemistry in the near future. Our laboratory is working on expanding the palette of opportunities on this front.

## Supporting Information Available

Experimental procedures and analytical data (^1^H, ^13^C and ^19^F NMR, HRMS, X-ray crystallographic details). (PDF)
