# Supplementary material for: Reductive Cyclopropanation through Bismuth Photocatalysis
Source: J Am Chem Soc. 2024 Aug 5;146(32):22140–4. doi: 10.1021/jacs.4c07262 (PMC11328130; doi:10.1021/jacs.4c07262)

Supporting Information

Reductive Cyclopropanation through Bismuth Photocatalysis

Shengyang Ni, Davide Spinnato and Josep Cornella*

Max-Planck-Institut für Kohlenforschung, Kaiser-Wilhelm-Platz 1, Mülheim an der Ruhr, 45470, Germany

Table of Contents

1 General Methods 2
2 Optimization table 3
2.1 Different catalysts 3
2.2 Different reductants 3
2.3 Different loadings of CH_2I_2 4
2.4 Different solvents 4
2.5 Different lights 5
3 Stoichiometric experiments 6
3.1 Preparation of oxidative addition complex **34** 6
3.2 Preparation of oxidative addition complex **36** 11
3.3 Stoichiometric experiments of complex **34** and **35** 16
3.4 Stoichiometric experiments of complex **36** 17
3.5 Radical trapping experiments 18
3.6 Reduction of Bi(III) 19
3.7 Photo NMR experiments 20
3.8 Radical clock experiments 21
4 Synthesis of the 1,3-diiodoalkyl substrates 23
5 Unsuccessful substrates 27
6 General procedure for Bi-catalyzed reductive cyclopropanation with light (General Procedure A) 28
7 General procedure for Bi-catalyzed reductive cyclopropanation with light (General Procedure B) 28
8 Characterization Data 30
9 References 46
10 NMR Spectra 48

1 General Methods

Instruments

GC-MS (FID): GC-MS-QP2010 equipped (Shimadzu Europe Analytical Instruments). ESI-MS: ESQ 3000 (Bruker). Accurate mass determinations: Bruker APEX III FT-MS (7 T magnet) or MAT 95 (Finnigan). Melting points were measured with an EZ-Melt Automated Melting Point Apparatus from Standford Research Systems. NMR spectra were recorded using a Bruker AVIIIHD 300 MHz, Bruker AVneo 500 MHz or Bruker AVneo 600 MHz NMR spectrometer. The chemical shifts (δ) are given in ppm and were measured relative to solvent residual peak as an internal standard. For ^{1}H NMR: $CDCl_3$, δ 7.26; CD_3CN, δ 1.940. For ^{13}C NMR: $CDCl_3$, δ 77.16; CD_3CN, δ 1.32. The data is being reported as (s = singlet, d = doublet, t = triplet, q = quartet, quint = quintet, m = multiplet or unresolved br s = broad signal, coupling constant(s) in Hz, integration, interpretation). UV-Vis absorption spectra were recorded on a Cary 6000i UV-Vis-NIR Spectrophotometer.

Chemicals

Unless otherwise stated, all manipulations were performed using Schlenk techniques under dry argon in heatgun-dried glassware. Unless otherwise noted, all reagents were obtained from commercial suppliers and used without further purification. Anhydrous DMA (1.00 L, 99.8%) and anhydrous MeCN (1.00 L, 99.8%) were purchased from Sigma-Aldrich, stored directly in the glovebox, and use as received. Zinc powder (325 mesh, 99.9% (metal basis)) was purchased from Alfa Aesar and manganese powder (≥99.9% trace metals basis) was purchased from Sigma-Aldrich, stored directly in the glovebox, and used as received. Non-commercially available substrates were prepared according to procedures reported in the literature.[1] The bismuth catalysts were synthesized according the literture.[2]

2 Optimization table

2.1 Different catalysts

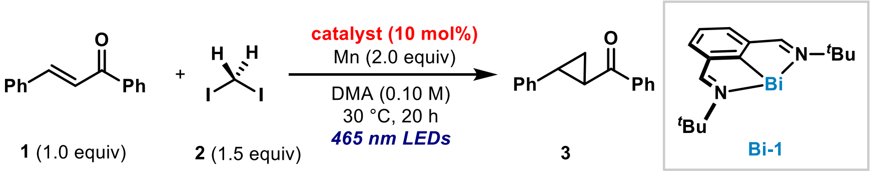

entry	different catalyst	yield of **3** (%)[b]
1	Bi-1	74
2	Bi-1•Cl_2	68
3	$BiPh_3$	trace
4	$BiCl_3$	57
5	$NiCl_2$	trace
6	$CuCl_2$	ND
7	no catalyst	ND

Note: Yields were determined by crude ^{1}H NMR using 1,3,5-trimethoxybenzene as internal standard.

2.2 Different reductants

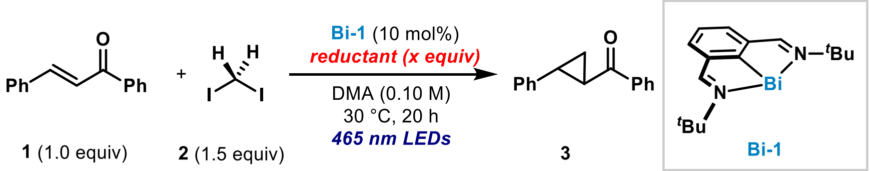

entry	reductant (x equiv)	yield of **3** (%)[b]
1	Mn (2.0 equiv)	74
2	Zn (2.0 equiv)	30
3	Hantzsch ester (2.0 equiv)	trace
4	TMS_3SiH (2.0 equiv)	trace
5	Mn (1.0 equiv)	57
6	$CoCp_2$ (2.0 equiv)	18

Note: Yields were determined by crude ^{1}H NMR using 1,3,5-trimethoxybenzene as internal standard.

2.3 Different loadings of CH_2I_2

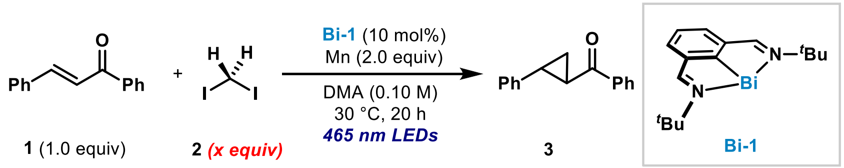

entry	CH_2I_2 stoichiometry (x equiv)	yield of **3** (%)[b]
1	1.0 equiv	48
2	1.5 equiv	74
3	2.0 equiv	74

Note: Yields were determined by crude ^{1}H NMR using 1,3,5-trimethoxybenzene as internal standard.

2.4 Different solvents

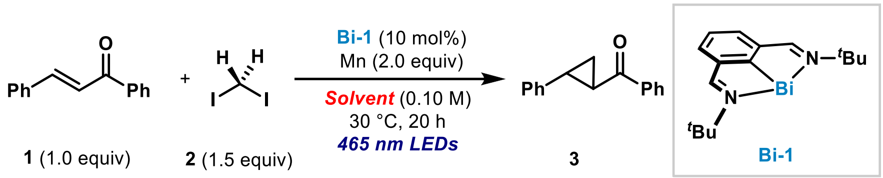

entry	solvent	yield of **3** (%)[b]
1	DMA	74
2	DMF	47
3	DMSO	45
4	toluene	trace
5	CH_3CN	51
6	THF	trace

Note: Yields were determined by crude ^{1}H NMR using 1,3,5-trimethoxybenzene as internal standard.

2.5 Different lights

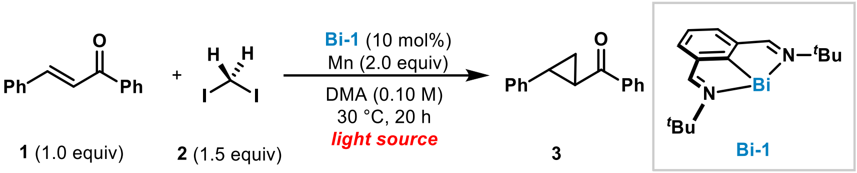

entry	light source	yield of **3** (%)[b]
1	465 nm LEDs	74
2	390 nm LEDs	trace
3	525 nm LEDs	42
4	625 nm LEDs	trace

Note: Yields were determined by crude ^{1}H NMR using 1,3,5-trimethoxybenzene as internal standard.

3 Stoichiometric experiments

3.1 Preparation of oxidative addition complex 34

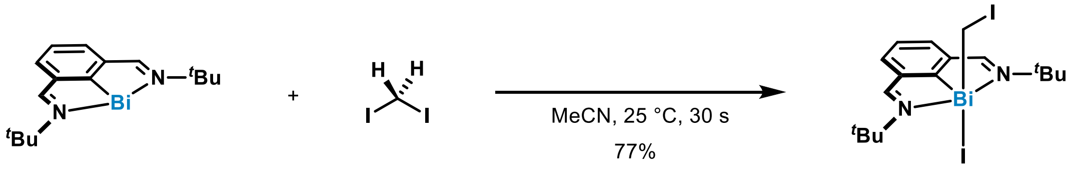

Bi-1, 0.10 mmol **2** (5.0 equiv) **34**

In an argon-filled glove box, **Bi-1** (45.2 mg, 0.100 mmol) was placed in an oven-dried 50 mL Schlenk flask. Dry MeCN (10 mL, 0.010 M) was added using a syringe, and **2** (41 µL, 0.50 mmol) was added via a microsyringe. The resulting light yellow mixture was stirred for 30 seconds. The schlenk flask was taken out of the glovebox and the mixture was concentrated under reduced pressure. The obtained yellow solid was washed with pentane (×5, approximately 10 mL in total) and Et_2O (×5, approximately 10 mL in total) and dried to afford the product in 77% yield (55.4 mg) as a pale yellow solid.

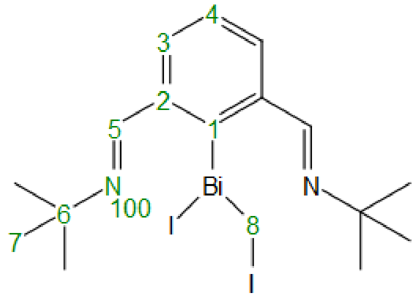

^{1}H NMR (600 MHz, CD_3CN) δ 9.88 (s, 2H, H-5), 8.26 (d, J = 7.6 Hz, 2H, H-3), 8.03 – 7.97 (m, 1H, H-4), 3.24 (s, 2H, H-8), 1.51 (s, 18H, H-7).

^{13}C NMR (151 MHz, CD_3CN) δ 195.9 (C-1), 169.1 (C-5), 149.0 (C-2), 137.4 (C-3), 131.3 (C-4), 62.1 (C-6), 31.1 (C-7), 12.9 (C-8).

Note: From the DEPT135-edited HSQC data, the carbon at C8 is a methylene group (CH_2). The NMR shifts of pos 8 is rather unusual (^{1}H: 3.24 ppm, ^{13}C: 12.93 ppm). The connectivity to the rest of the complex could be confirmed from one HMBC cross peak of H8 to C1 and a NOESY cross peak between H8 and H7.

Compound 34 ^{1}H NMR

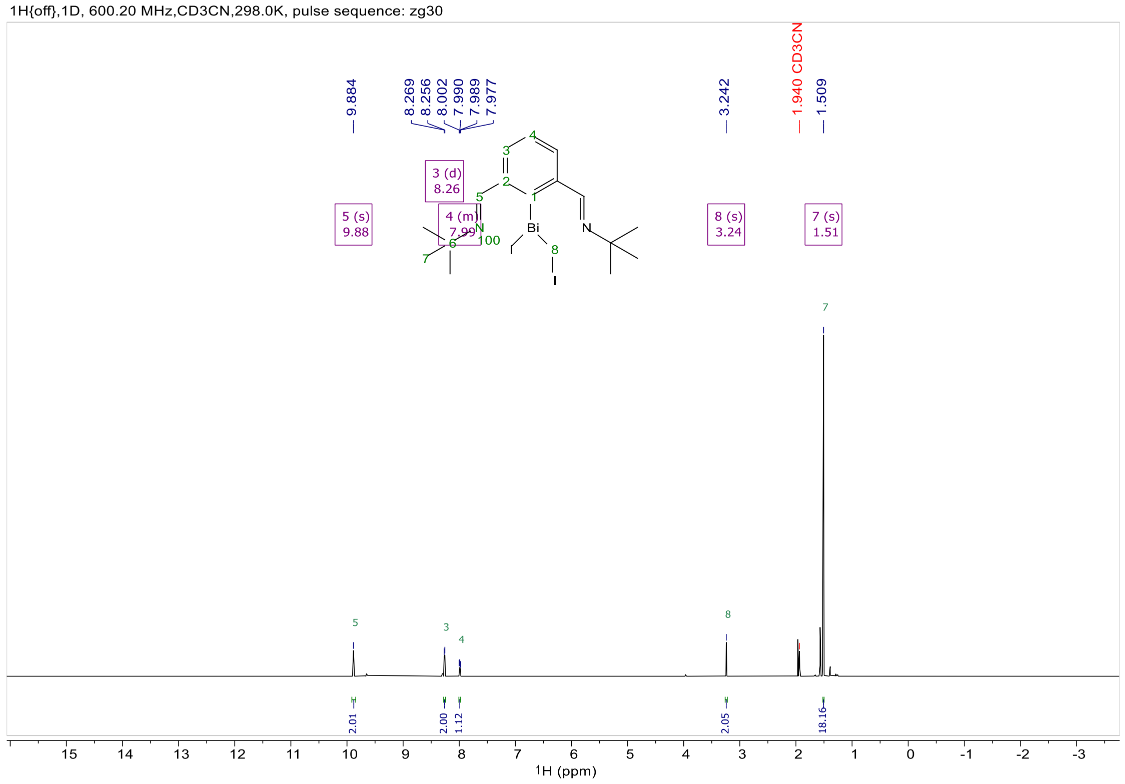

Compound 34 ^{13}C NMR

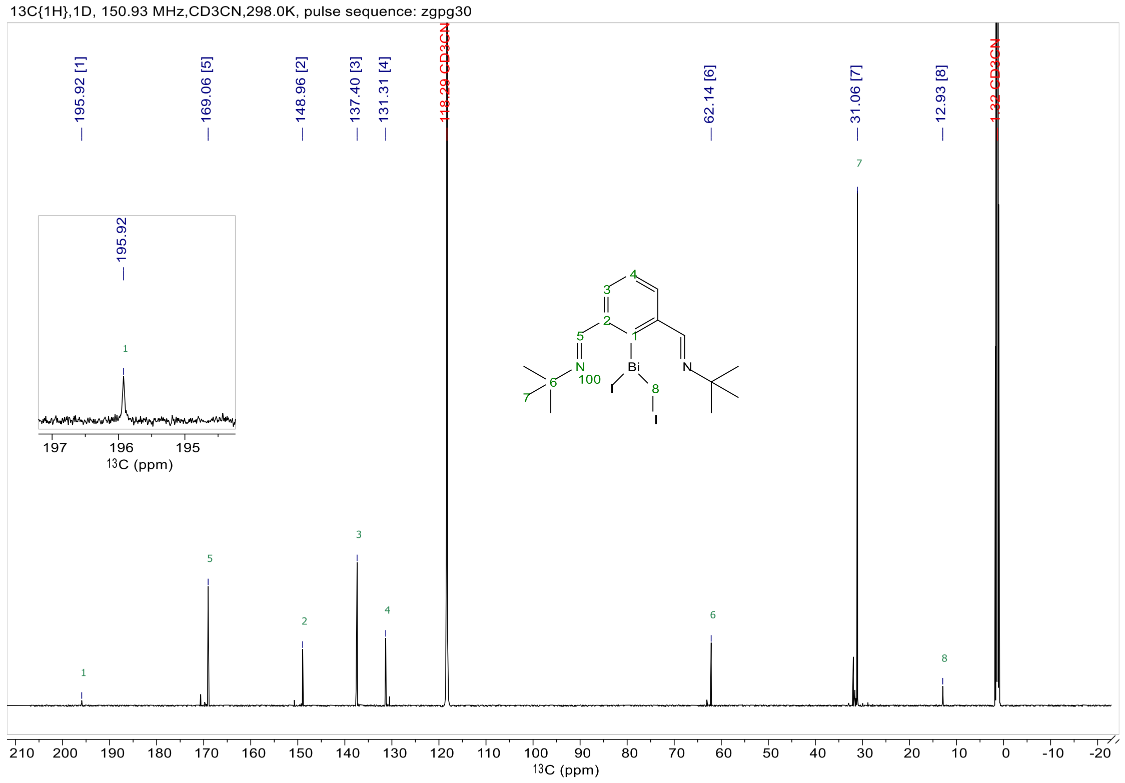

Compound 34 HSQC

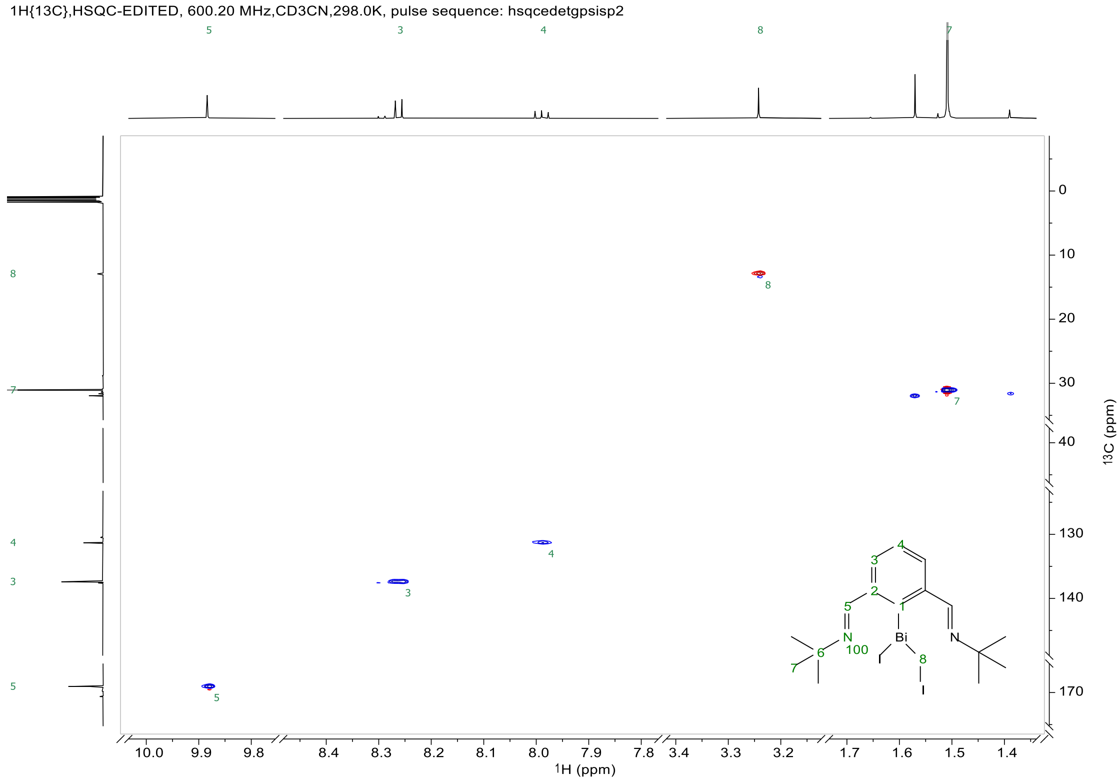

Compound 34 HMBC

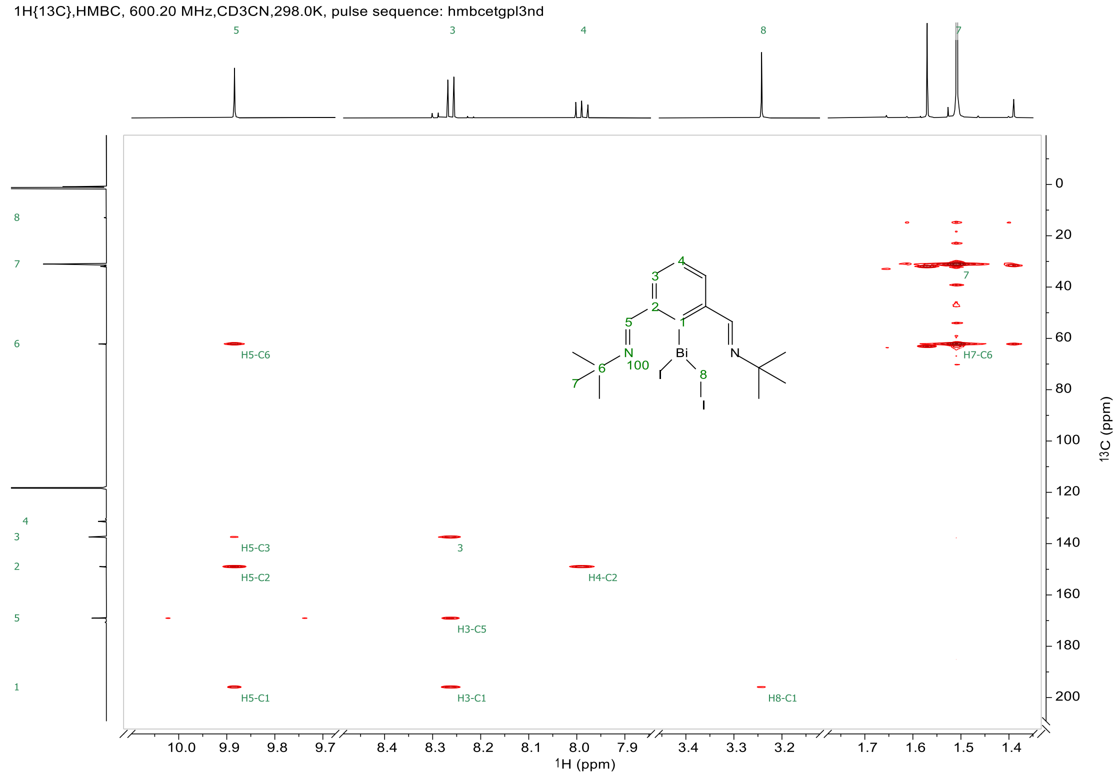

Compound 34 COSY

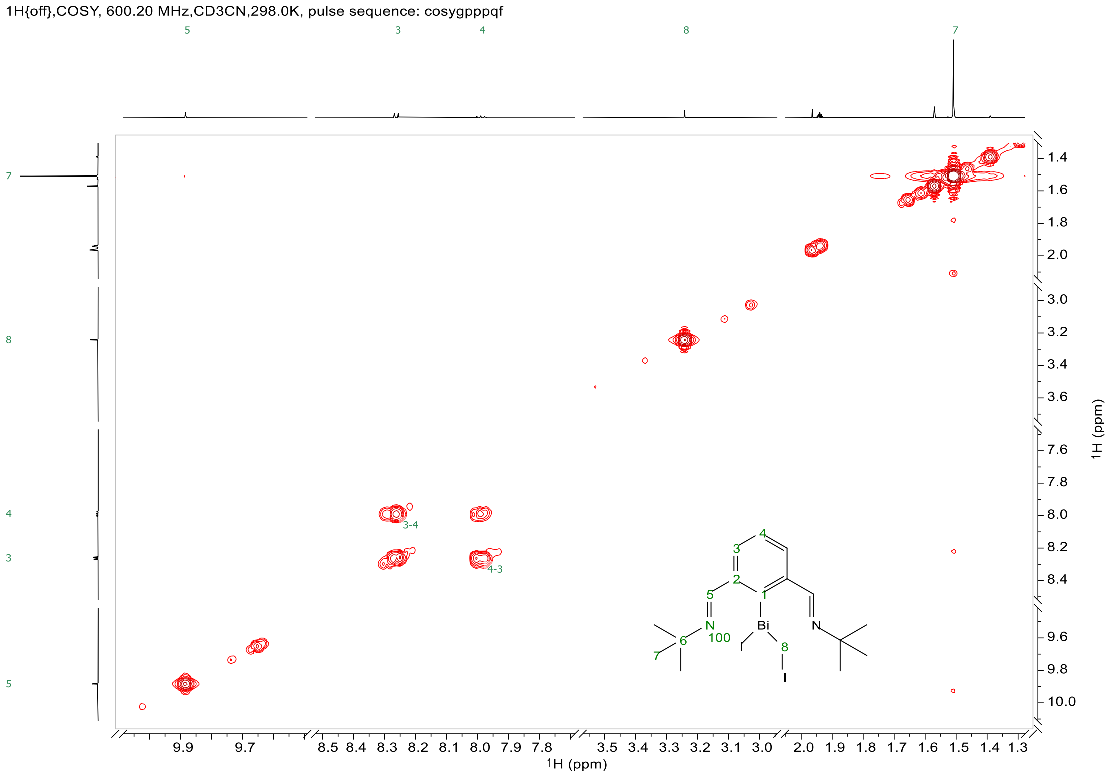

Compound 34 NOESY

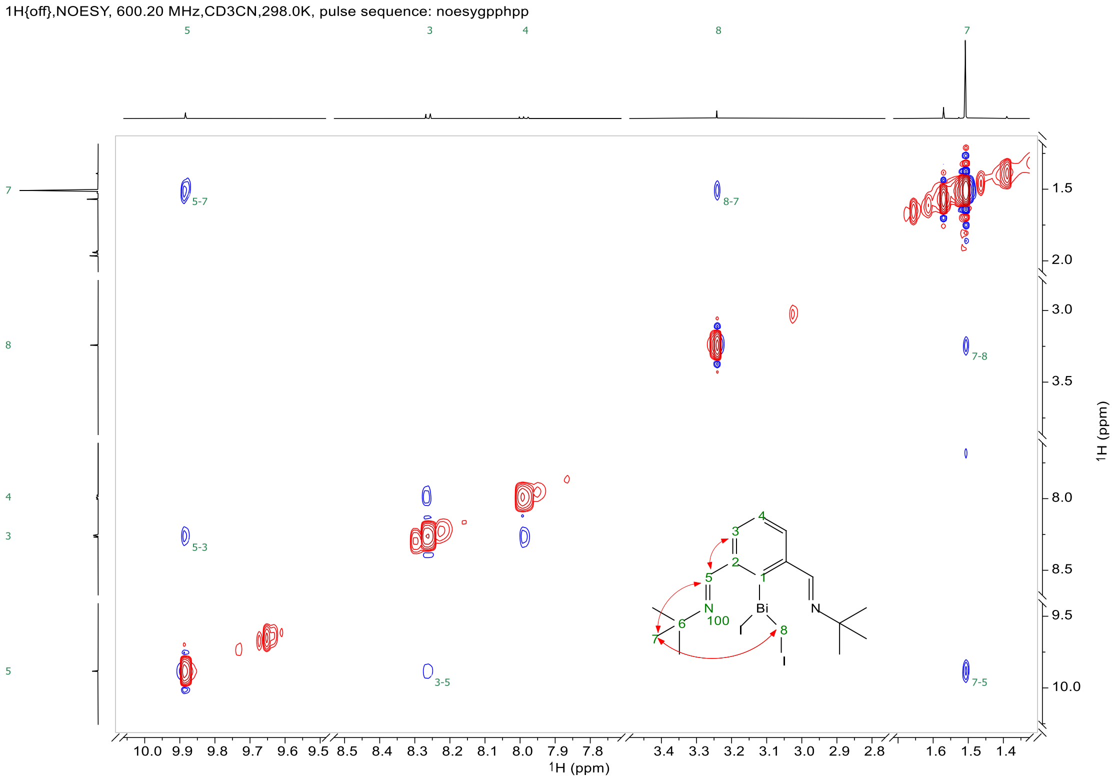

Compound 34 H-N HMBC

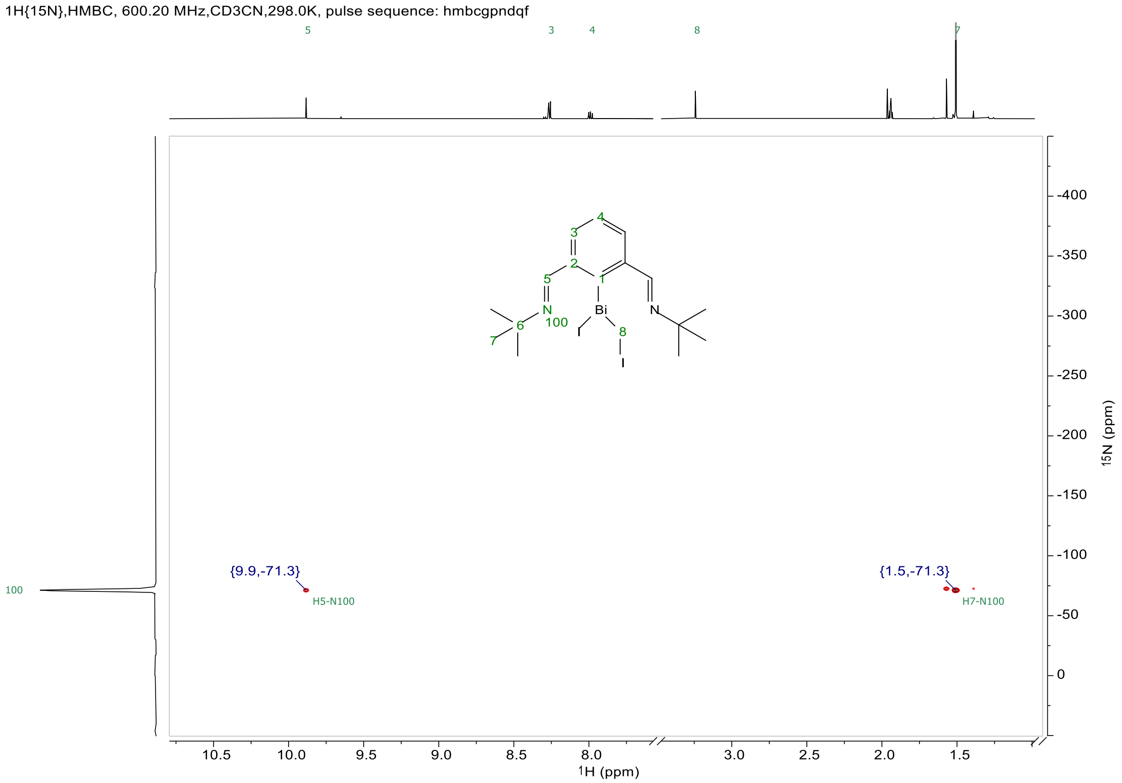

HRMS (ESI): calc'd for $C_{17}H_{25}Bi_1I_1N_2$ $[M-I]^+$: 593.0861, found: 593.0861.

UV-Vis measurement

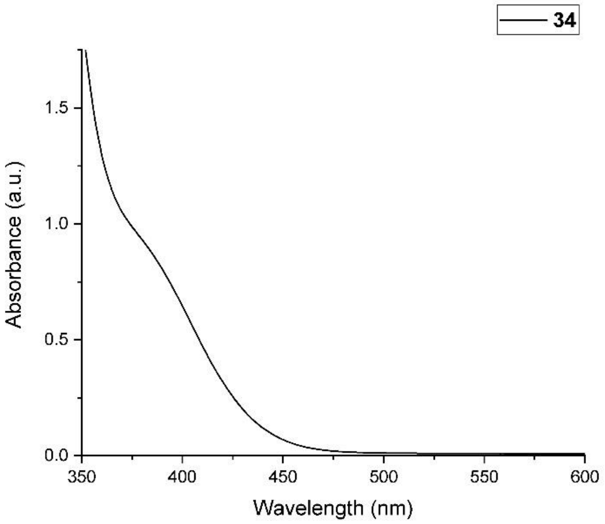

Figure S1. UV-Vis absorption spectrum of **34** in CH_3CN. The sample was diluted with CH_3CN to 4.0 mM and sealed in a cuvette (0.10 cm). **34** displayed an inset of absorption at ca. 480nm.

3.2 Preparation of oxidative addition complex 36

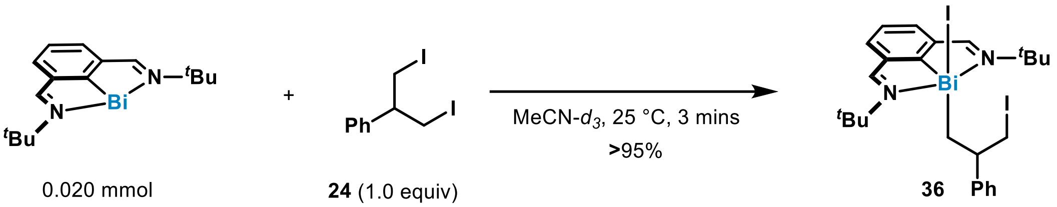

In an argon-filled glove box, **Bi-1** (9.1 mg, 0.020 mmol) was placed in an oven-dried reaction tube. MeCN-d_3 (1.0 mL, 0.050 M) was added using a syringe, and **24** (7.4 mg, 0.020 mmol) was added via a microsyringe. The reaction was stirred for 3 min and the color changed from dark green to light yellow. After 3 min, the mixture was transferred to the NMR tube and analyzed directly.

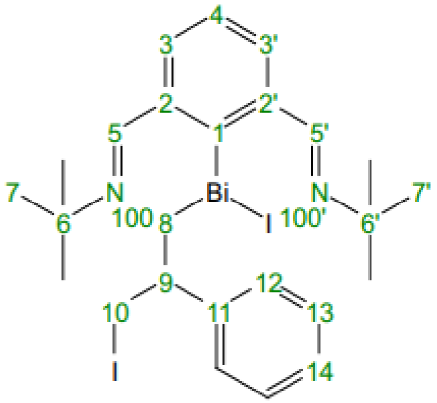

^{1}H NMR (600 MHz, CD$_3$CN) δ 9.79 (s, 1H, 5), 9.25 (s, 1H, 5'), 8.21 (m, 1H, 3), 7.93 – 7.83 (m, 2H, 3', 4), 7.10 – 7.05 (m, 1H, 14), 7.01 – 6.97 (m, 2H, 13), 6.61 – 6.57 (m, 2H, 12), 3.68 – 3.60 (m, 1H), 3.28 (dd, J = 9.8, 8.1 Hz, 1H, 10'), 3.13 (dd, J = 9.8, 6.2 Hz, 1H, 10"), 2.47 (dd, J = 12.7, 10.5 Hz, 1H, 8'), 2.40 (dd, J = 12.7, 3.6 Hz, 1H, 8"), 1.49 (s, 9H, 7), 1.41 (s, 9H, 7').

^{13}C NMR (151 MHz, CD$_3$CN) δ 185.8 (C-1), 169.8 (C-5), 169.1 (C-5'), 149.6 (C-2'), 148.9 (C-2), 144.3 (C-11), 137.4 (C-3'), 137.2 (C-3'), 130.8 (C-4), 129.0 (C-13), 128.1 (C-12), 128.0 (C-14), 62.2 (C-6), 62.1 (C-6'), 59.4 (C-8), 48.4 (C-9), 31.3 (C-7'), 31.1 (C-7"), 24.4 (C-10).

Note: C-8 is characteristically broadened due to relaxation effects from residual coupling to ^{209}Bi. Additionally, H-8 shows a cross peak to C-1 in the ^{1}H, ^{13}C-HMBC. The 2 sides of the *N*,*C*,*N*-pincer ligand are diastereotopic due to the chiral center of C-9 and a hindered rotation around the

C1-Bi bind. H-12 shows a characteristic NOE to H-7', but not to H-7. This is also in line with a shielding observed of H-7' compared relative to H-7 (aromatic ring current).

Over the course of a weekend in the fridge, a conversion of the major component to cyclopropyl-benzene and a likely [Bi]·I_2 species are observed.

Compound 36 ^{1}H NMR

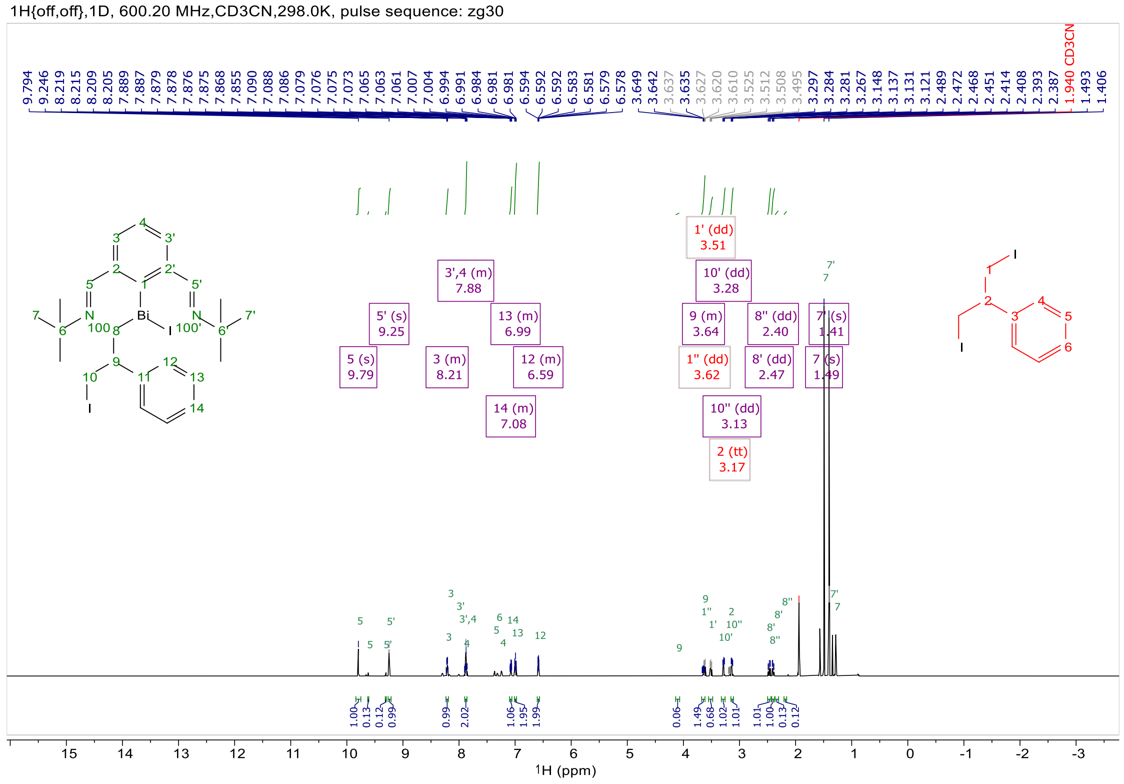

Compound 36 ^{13}C NMR

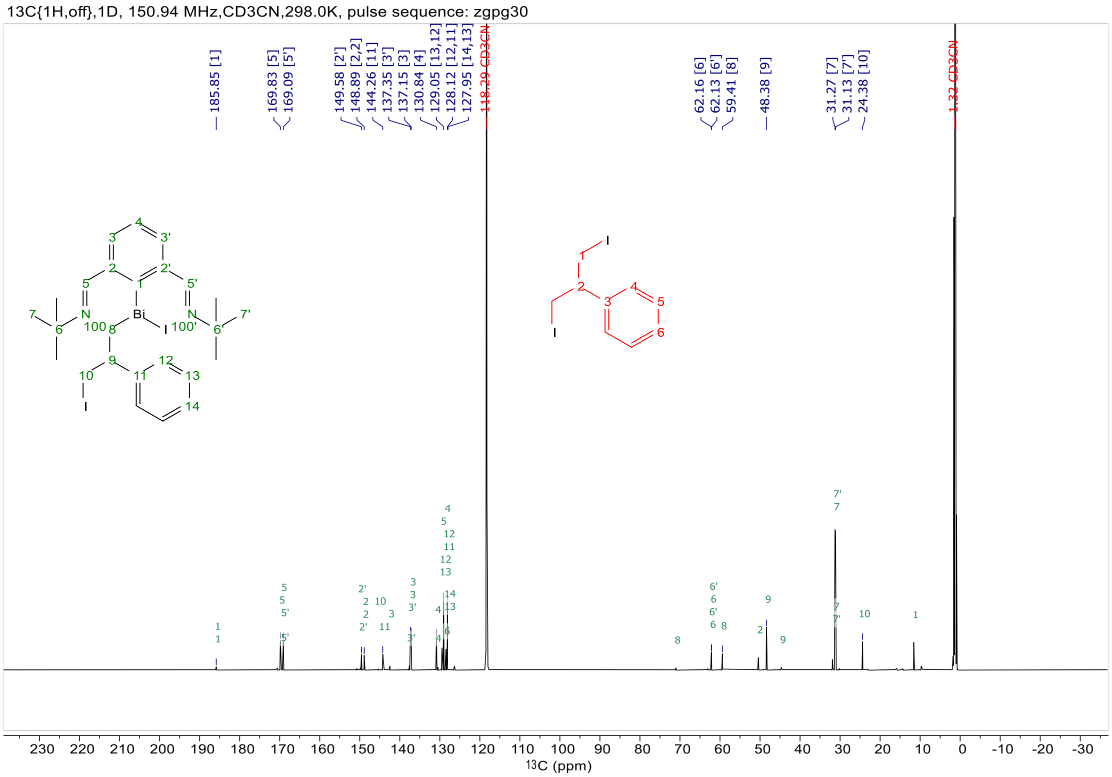

Compound 36 HSQC

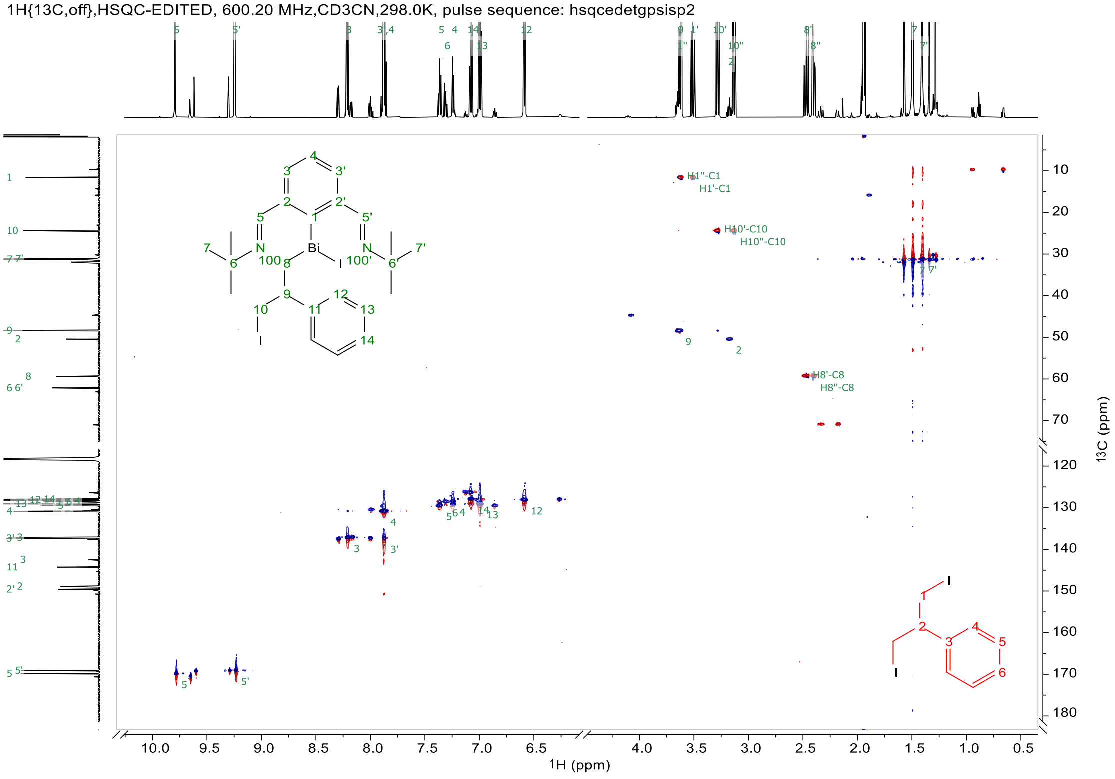

Compound 36 HMBC

1H{13C,off},HMBC, 600.20 MHz,CD3CN,298.0K, pulse sequence: hmbcetgpl3nd

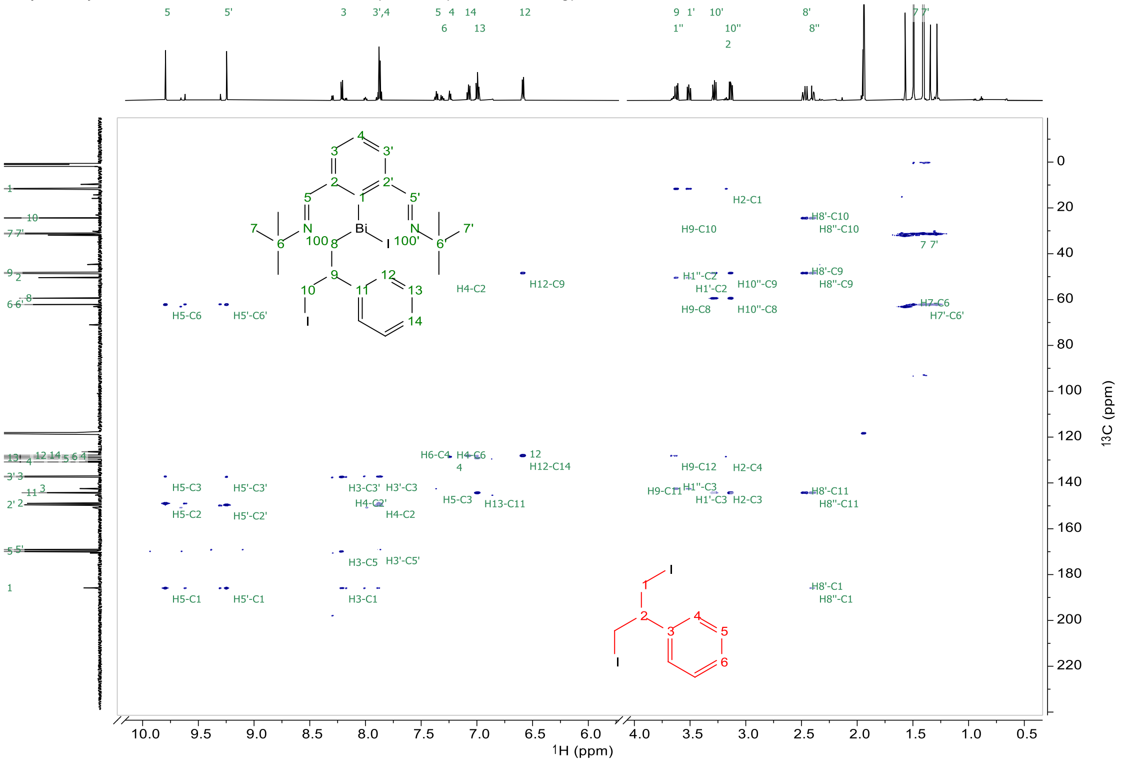

Compound 36 COSY

1H{off,off},COSY, 600.20 MHz,CD3CN,298.0K, pulse sequence: cosygpppqf

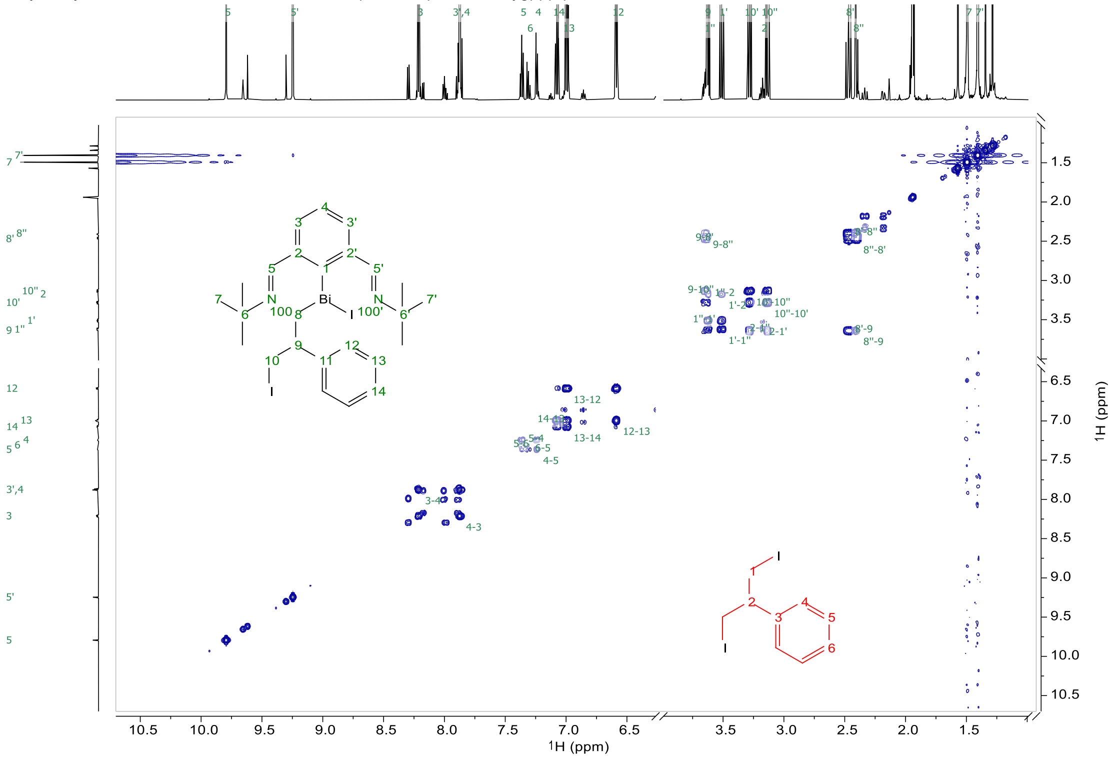

Compound 36 NOESY

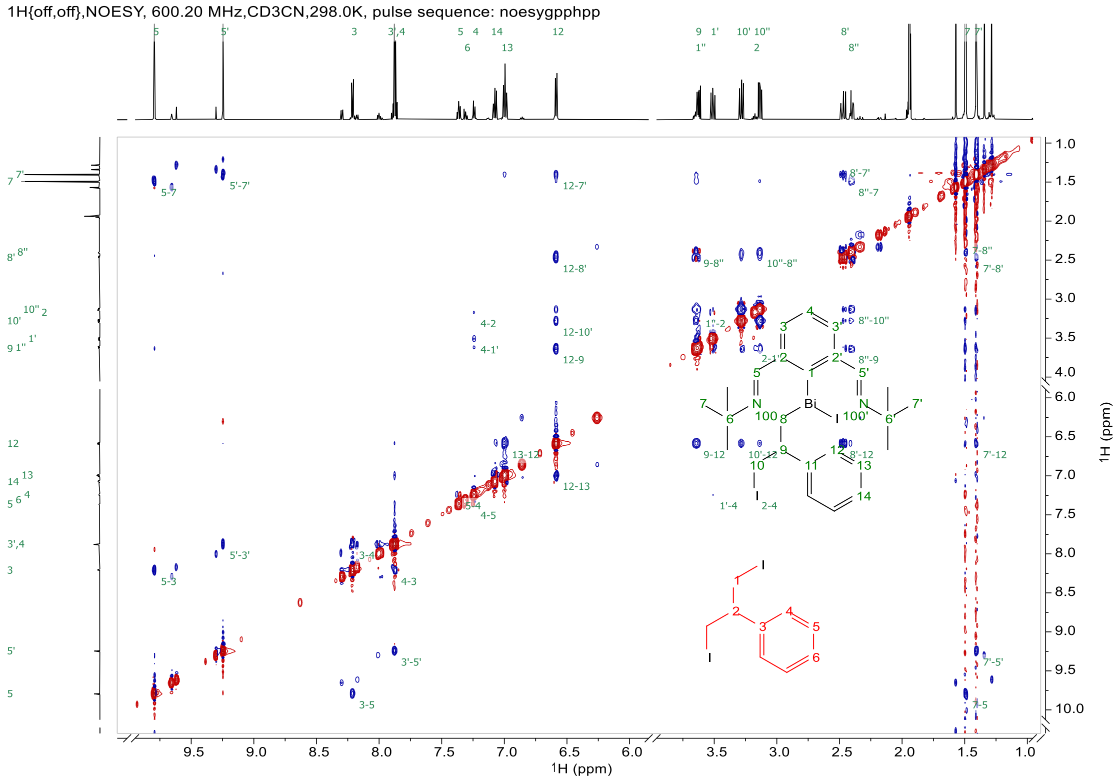

Compound 36 H-N HMBC

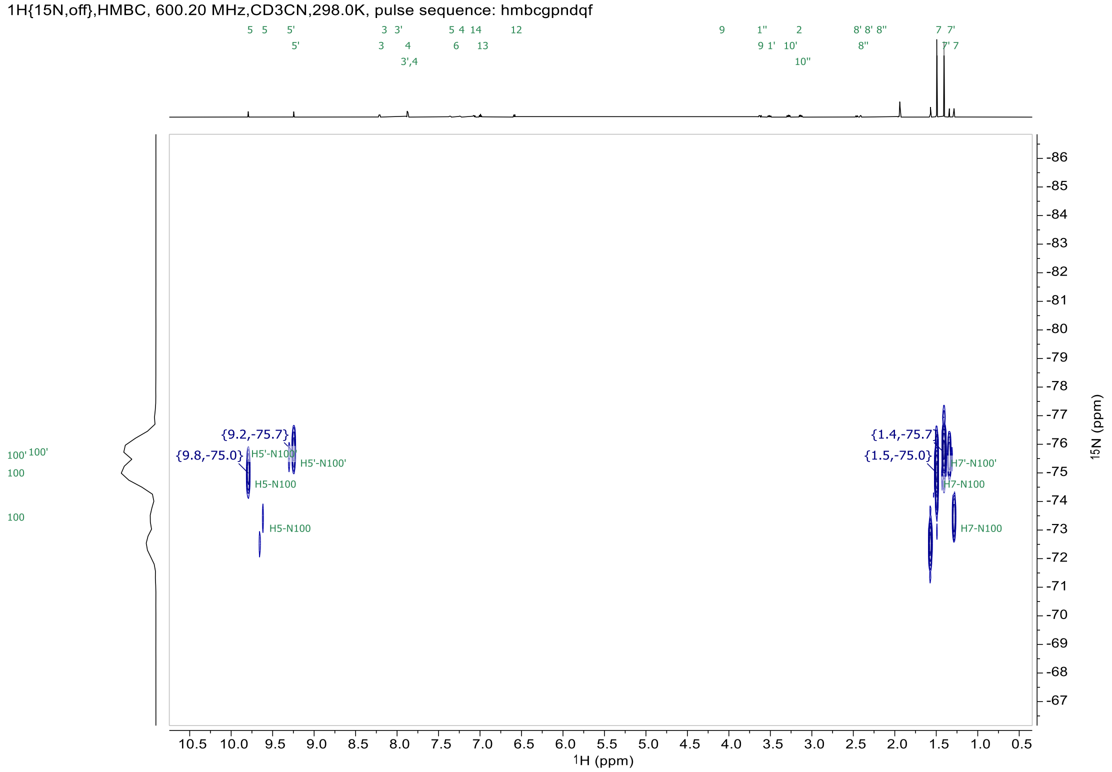

HRMS (ESI): calc'd for $C_{25}H_{33}Bi_1I_1N_2$ [M–I]$^+$: 697.1487, found: 697.1484.

UV-Vis measurement

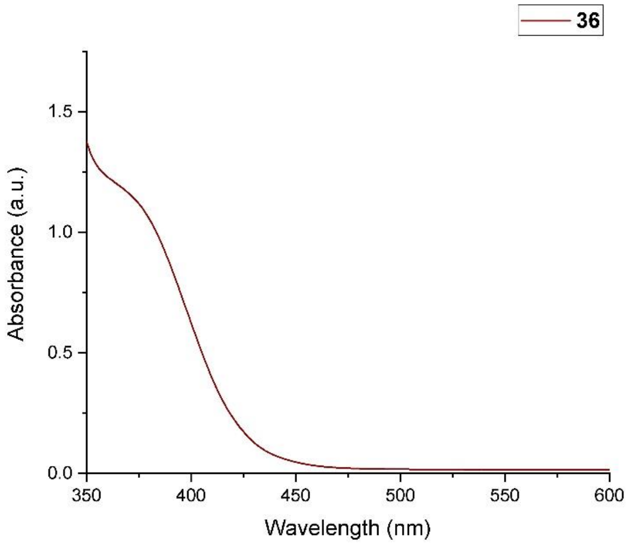

Figure S2. UV-Vis absorption spectrum of **36** in MeCN. The sample was diluted with MeCN to 4.0 mM and sealed in a cuvette (0.10 cm). **36** displayed an inset of absorption at ca. 470 nm.

3.3 Stoichiometric experiments of complex 34 and 35

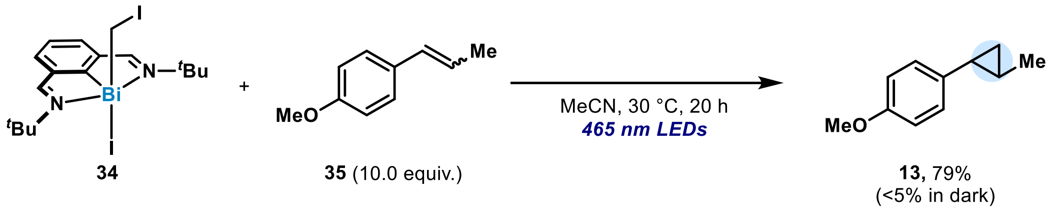

A culture tube with a Teflon screw-cap equipped with a Teflon-coated stir bar was used. The culture tube was brought into an argon-filled glovebox, complex **34** (0.010 mmol, 1.0 equiv) was introduced into the culture tube and MeCN (0.5 mL, 0.1 M) was added using a syringe. Then, outside the glovebox, **35** (14.8 mg, 0.100 mmol, 10.0 equiv) was added to the culture tube under Ar via a microsyringe. The reaction tube was sealed with parafilm and placed it into a 465 nm blue LEDs (LEDXON® 24 V LED band) reactor with a cooling fan to keep the temperature 30 °C. After 20 h, the mixture was diluted with MTBE (approximately 4 mL), washed with brine

(approximately 4 mL), and dried over Na_2SO_4. Upon filtration, the organic layer was concentrated under reduced pressure (water bath at 40 °C) and the yield (79%) was determined by ^1H NMR using 1,1,2-trichloroethene as internal standard.

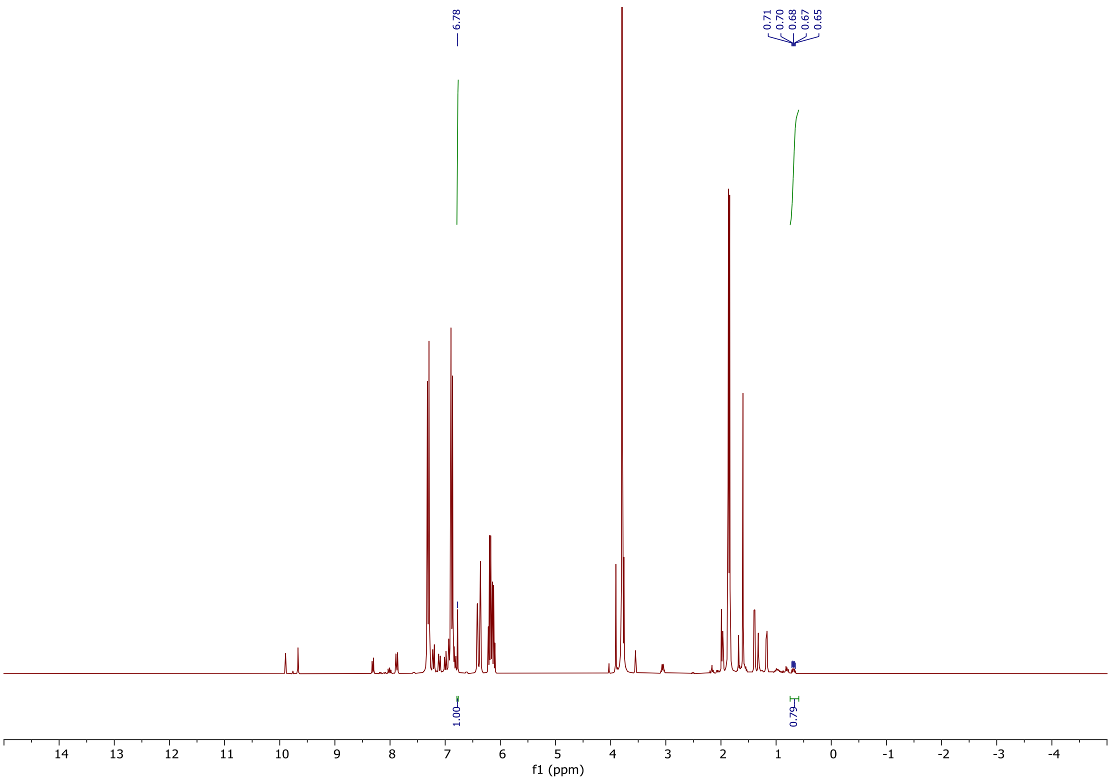

3.4 Stoichiometric experiments of complex 36

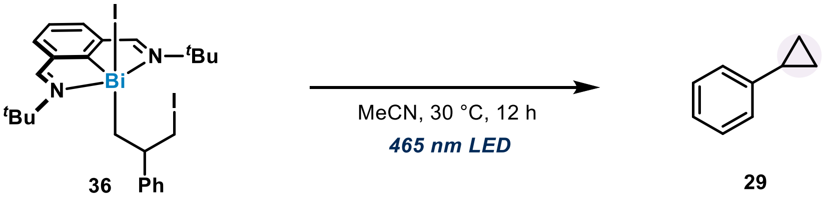

A culture tube with a Teflon screw-cap equipped with a Teflon-coated stir bar was used. The culture tube was brought into an argon-filled glovebox, complex **36** (0.010 mmol, 1.0 equiv) was introduced into the culture tube and MeCN (0.5 mL, 0.1 M) was added using a syringe. Then, outside the glovebox, **t**he reaction tube was sealed with parafilm and placed it into a 465 nm blue LEDs (LEDXON® 24 V LED band) reactor with a cooling fan to keep the temperature 30 °C. After 20 h, the mixture was diluted with MTBE (approximately 4 mL), washed with brine (approximately 4 mL), and dried over Na_2SO_4. Upon filtration, the organic layer was concentrated

under reduced pressure (water bath at 40 °C) and the yield (78%) was determined by ^{1}H NMR using 1,3,5-trimethoxybenzene as internal standard.

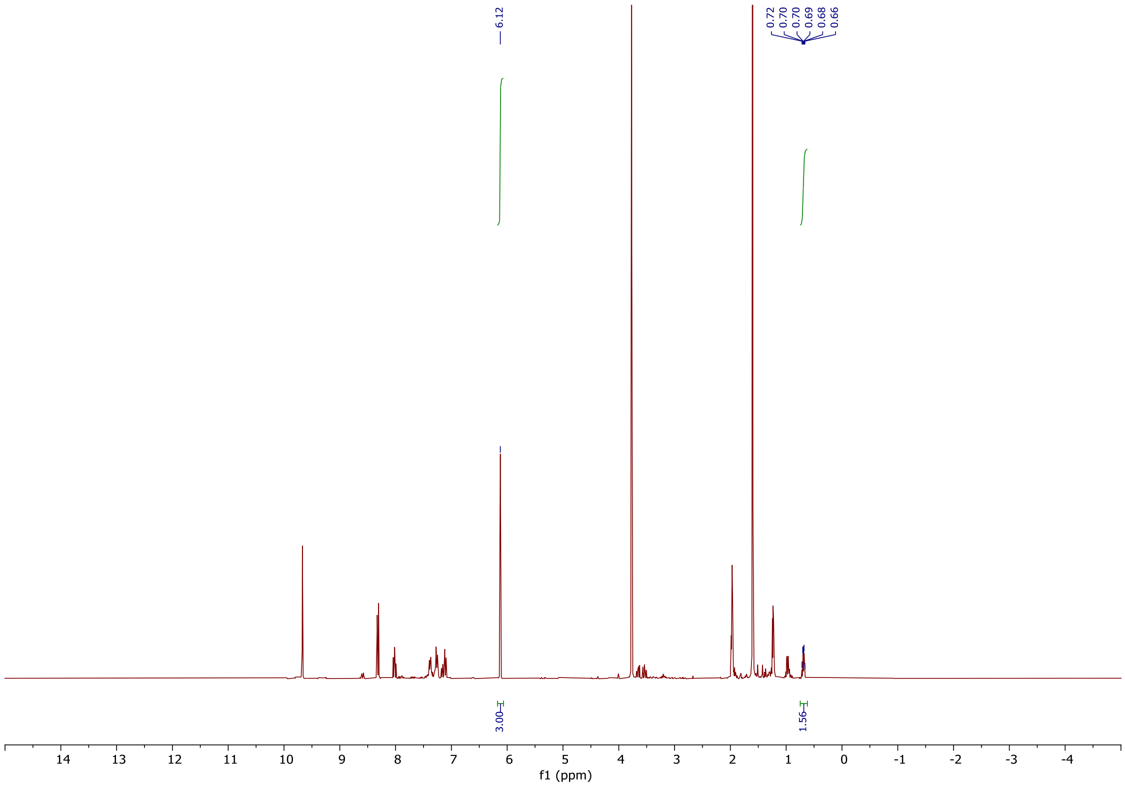

3.5 Radical trapping experiments

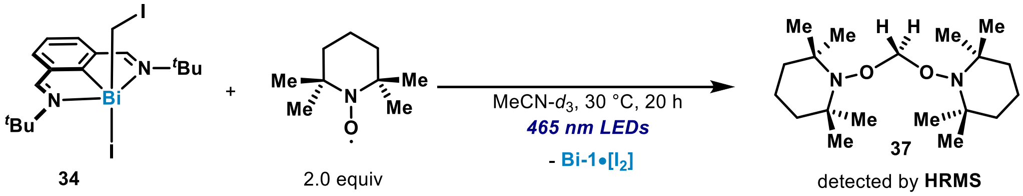

In an argon-filled glove box, **34** (7.2 mg, 0.010 mmol) and TEMPO (31.2 mg, 0.0200 mmol) was placed in an oven-dried reaction tube, MeCN-*d*3 (0.5 mL, 0.002 M) was added using a syringe. The reaction tube was sealed with parafilm and placed it into a 465 nm blue LEDs (LEDXON® 24 V LED band) reactor with a cooling fan to keep the temperature 30 °C. After 20 h, **37** was detected by HRMS.

HRMS (ESI-TOF): calc'd for $C_{19}H_{38}N_2O_2$ $[M+H]^+$: 327.3006, found: 327.3008.

3.6 Reduction of Bi(III)

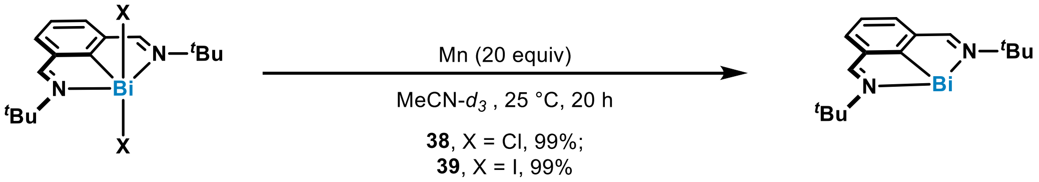

A culture tube with a Teflon screw-cap equipped with a Teflon-coated stir bar was used. The culture tube was brought into an argon-filled glovebox, **Bi-1·X$_2$** (0.010 mmol, 1.0 equiv) and Mn (0.200 mmol, 20.0 equiv) were introduced into the culture tube and MeCN-*d$_3$* (0.5 mL, 0.1 M) was added using a syringe. The reaction mixture was stirred inside the glovebox for 20 h. Then, the reaction mixture was filtered by a HPLC filter and analyzed by ^{1}H NMR. The crude ^{1}H NMR shows both these 2 reaction have a quantitative yield by using 1,3,5-trimethoxybenzene as internal standard.

The crude ^{1}H NMR of reaction of compound **38**:

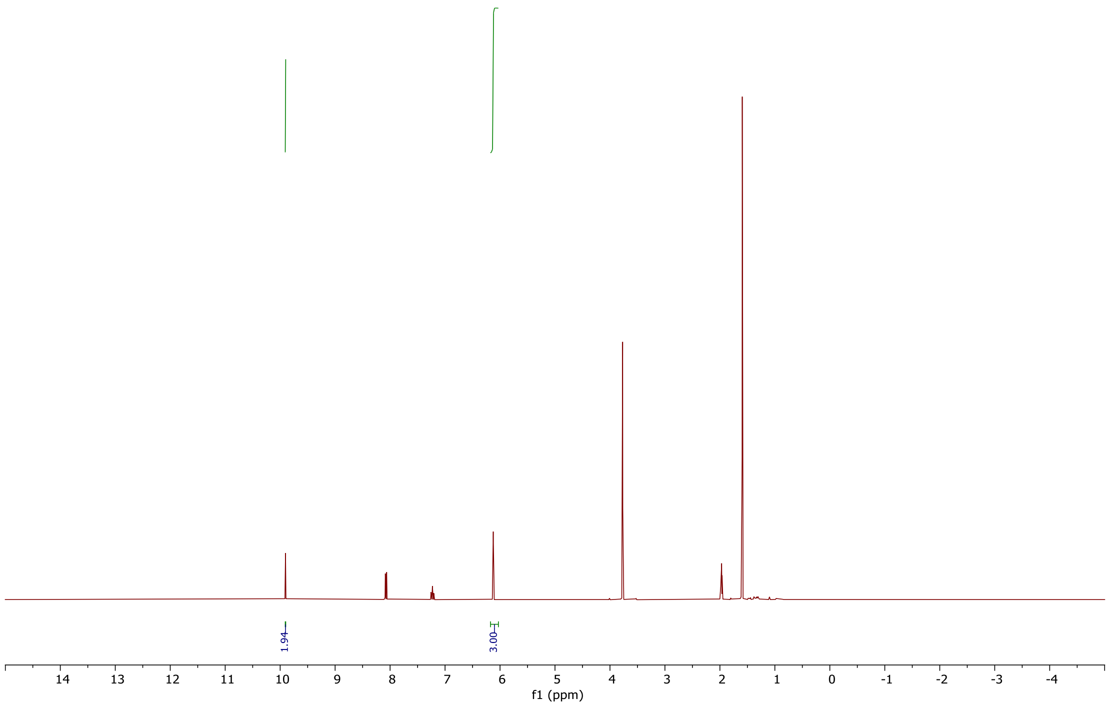

3.7 Photo NMR experiments

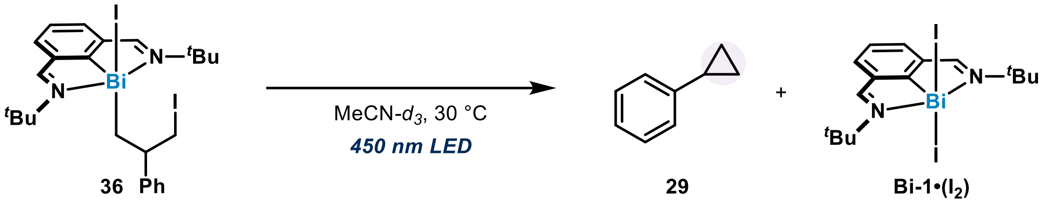

In an argon-filled glove box, **24** (7.4 mg, 0.020 mmol) was placed in an oven-dried culture tube and MeCN-d_3 (0.35 mL, 0.057M) was added. Then a stock solution of **Bi-1** (18.2 mg, 0.040 mmol) in MeCN-d_3 (0.70 mL, 0.057M) was prepared and 0.35 mL were added dropwise to the solution containing **24**. After few minutes under stirring the mixture turned yellow. Reaction monitoring under blue light was performed using a fiber-coupled LED NMR setup in analogy to our previous publication.[3] As a light source, a fiber-coupled ultra high power LED from Prizmatix Ltd. was used (LED head: UHP-T-450 SR, peak wavelength λ = 450 nm). Reactions were monitored as shown in Figure S3 with single scan ^{1}H NMR spectra for an appropriate time after the light was turned on and off. The raw NMR data was imported into MNOVA 14.3.1 with the reaction monitoring plugin and processed therein. The relative concentration profiles were referenced to the first acquired ^{1}H NMR spectrum of each reaction.

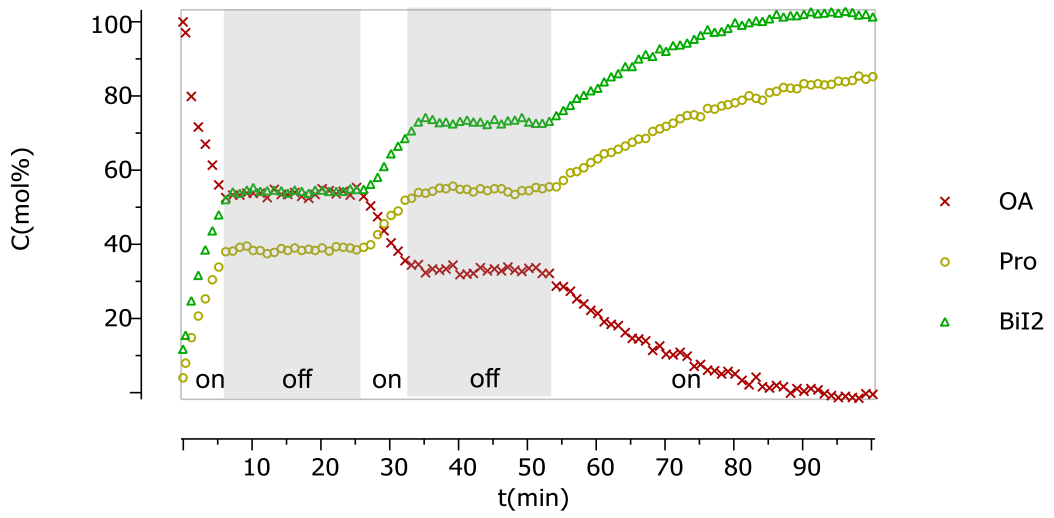

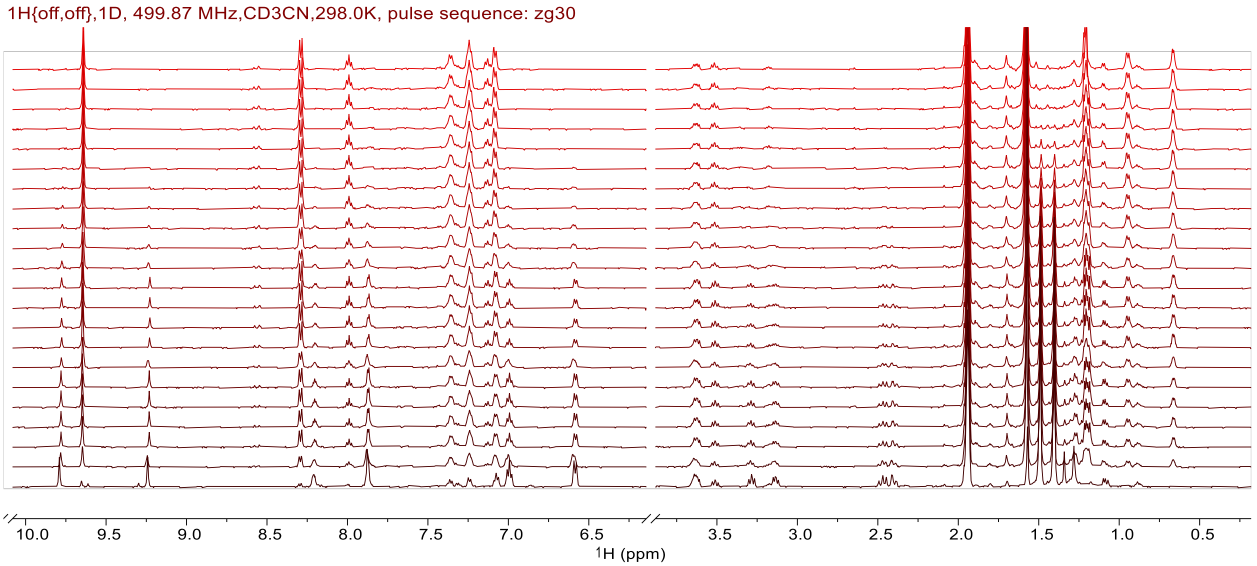

Figure S3. On/Off experiment via *in-situ* ^{1}H NMR analysis. Yellow profile **29**, red profile **36**, green profile **LBi-1•(I$_2$)**.

3.8 Radical clock experiments

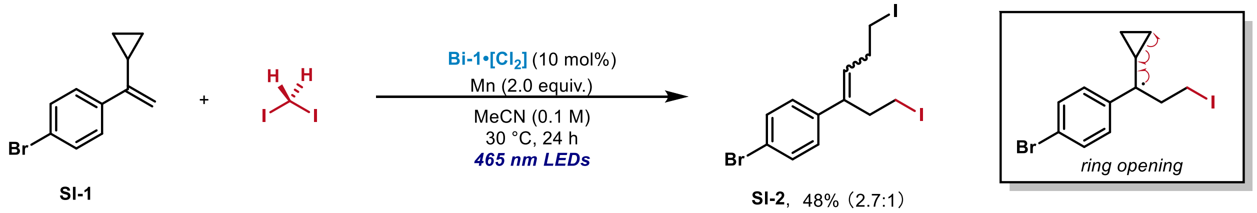

A culture tube with a Teflon screw-cap equipped with a Teflon-coated stir bar was used. The culture tube was brought into an argon-filled glovebox, **Bi-1·[Cl$_2$]** (5.2 mg, 0.010 mmol, 10 mol%) and Mn (0.20 mmol, 2.0 equiv) were introduced into the culture tube. Then, outside the glovebox, MeCN (1.0 mL, 0.10 M) was added using a syringe, **SI-1** (22.2 mg, 0.100 mmol, 1.00 equiv) and CH_2I_2 (53.4 mg, 0.200 mmol, 2.00 equiv) was added using microsyringes. The reaction tube was sealed with parafilm and placed it into a 465 nm blue LEDs (LEDXON® 24 V LED band) reactor with a cooling fan to keep the temperature 30 °C. After 24 h, the mixture was diluted with MTBE (approximately 4 mL), washed with brine (approximately 4 mL), and dried over Na_2SO_4. Upon filtration, the organic layer was concentrated under reduced pressure (water bath at 40 °C) and the

yield of the product **SI-2** was identified by ^{1}H NMR. Spectral data is in accordance with previous report.[4]

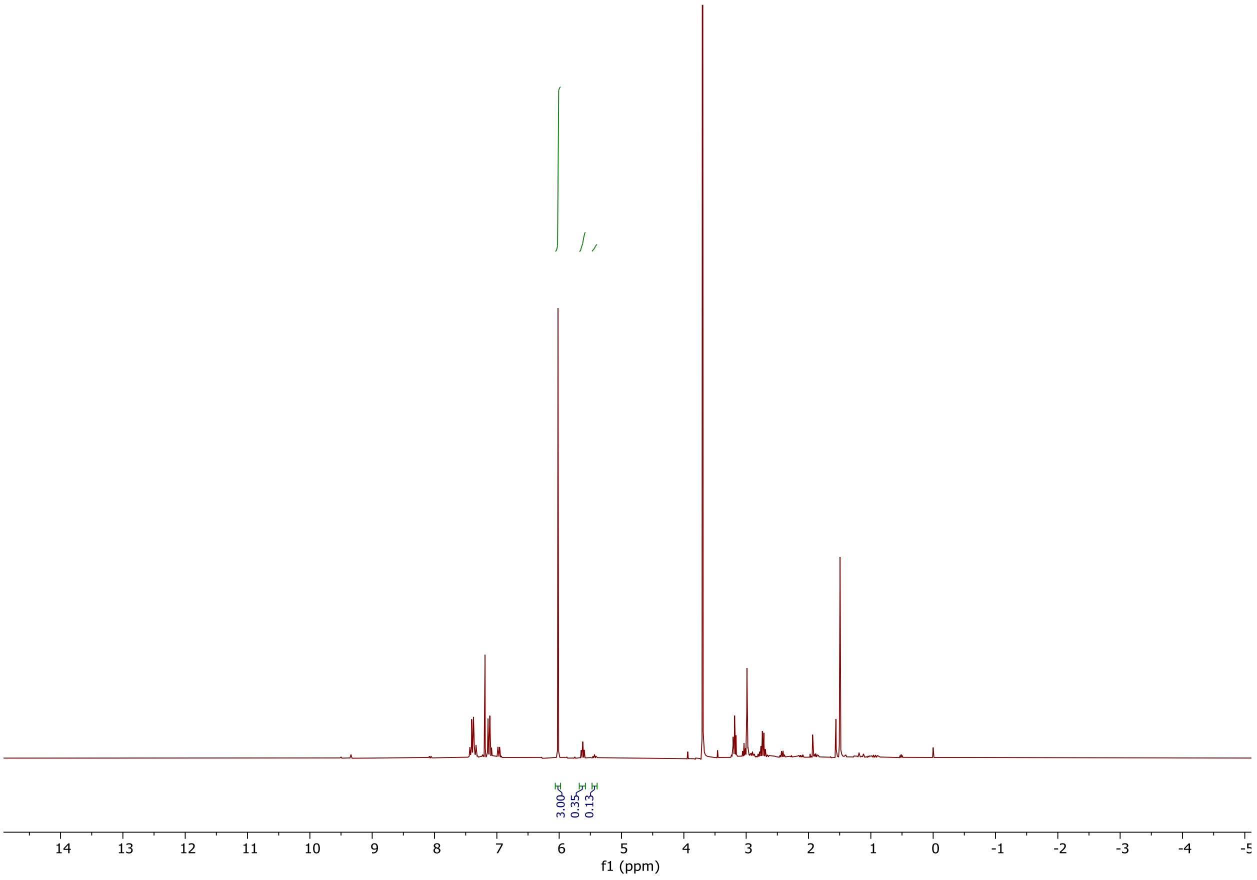

4 Synthesis of the 1,3-diiodoalkyl substrates

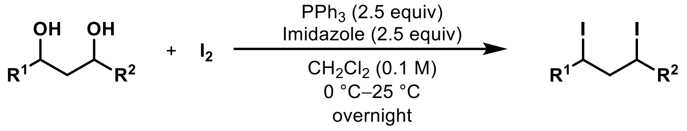

In an oven-dried 100 mL Schlenk tube under argon, equipped with a magnetic stir bar and septum, 1,3-diols (2.0 mmol), PPh_3 (5.0 mmol, 2.5 equiv) and imidazole (5.0 mmol, 2.5 equiv) was dissolved in dry CH_2Cl_2 (5.0 mL, 0.40 M). I_2 (5.0 mmol, 2.5 equiv) was added to the reaction tube over 20 mins at 0 °C under argon. After addition, the reaction mixture was allowed warm up to 25 °C. After overnight, the mixture was quenched with saturated NaS_2O_3 aqueous solution (approximately 20 mL) and diluted with CH_2Cl_2, washed with brine, and dried over Na_2SO_4. Upon filtration, the organic layer was concentrated under reduced pressure (water bath at 40 °C) and purified by flash column chromatography (silica gel) afford the desired product.

Compound 24

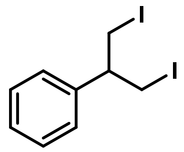

Following **the above Procedure** on 2.0 mmol scale. Purification by flash column chromatography (50:1 pentane:EtOAc) afforded 580 mg (78 %) of the title compound **24**.

Physical State: light yellow oil.

R_f = 0.55 (20:1 pentane:EtOAc).

^{1}H NMR (300 MHz, $CDCl_3$) δ 7.44 – 7.28 (m, 3H), 7.22 – 7.11 (m, 2H), 3.59 (dd, *J* = 10.0, 6.7 Hz, 2H), 3.50 (dd, *J* = 10.0, 6.9 Hz, 2H), 3.22 – 3.06 (m, 1H).

^{13}C NMR (75 MHz, $CDCl_3$) δ 141.0, 128.8, 128.0, 127.3, 49.5, 11.1.

HRMS (EI-TOF): calc'd for $C_9H_{10}I_2$ $[M]^+$: 371.8866, found: 371.8868.

Compound 25

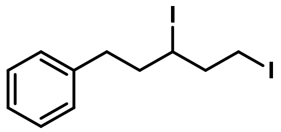

The corresponding diol was synthesized according to the literature.[5] Following **the above Procedure** on 2.0 mmol scale. Purification by flash column chromatography (50:1 pentane:EtOAc) afforded 496 mg (62 %) of the title compound **25**.

Physical State: dark brown oil.

R_f = 0.52 (20:1 pentane:EtOAc).

^{1}H NMR (300 MHz, CDCl$_3$) δ 7.31 – 7.04 (m, 5H), 4.12 – 3.93 (m, 1H), 3.41 – 3.25 (m, 1H), 3.22 – 3.10 (m, 1H), 2.92 – 2.77 (m, 1H), 2.72 – 2.59 (m, 1H), 2.34 – 1.85 (m, 4H).

^{13}C NMR (75 MHz, CDCl$_3$) δ 140.5, 128.7, 128.6, 126.4, 43.6, 41.9, 38.0, 35.6, 5.8.

HRMS (EI-TOF): calc'd for $C_{11}H_{14}I_2$ $[M]^+$: 399.9179, found: 399.3184.

Compound 26

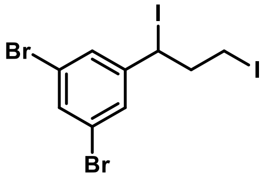

The corresponding diol was synthesized according to the literature.[5] Following **the above Procedure** on 1.5 mmol scale. Purification by flash column chromatography (50:1 pentane:EtOAc) afforded 403 mg (51 %) of the title compound **26**.

Physical State: dark brown oil.

R_f = 0.55 (20:1 pentane:EtOAc).

^{1}H NMR (300 MHz, CDCl$_3$) δ 7.56 (t, *J* = 1.7 Hz, 1H), 7.47 (d, *J* = 1.6 Hz, 2H), 5.08 (dd, *J* = 8.5, 6.4 Hz, 1H), 3.27 – 3.04 (m, 2H), 2.77 – 2.60 (m, 1H), 2.43 – 2.28 (m, 1H).

^{13}C NMR (75 MHz, CDCl$_3$) δ 146.4, 134.1, 129.4, 123.3, 43.4, 28.9, 4.5.

HRMS (ESI-TOF): calc'd for $C_9H_8Br_2I_2$ $[M+H]^+$: 528.7155, found: 528.7154.

Compound 27

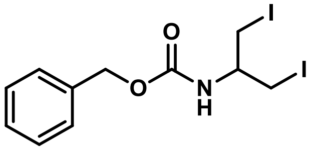

Following **the above Procedure** on 2.0 mmol scale. Purification by flash column chromatography (8:1 pentane:EtOAc) afforded 578 mg (65 %) of the title compound **27**.

Physical State: white solid.

m.p.: 108 – 110 °C.

R_f = 0.57 (4:1 pentane:EtOAc).

^{1}H NMR (300 MHz, $CDCl_3$) δ 7.45 – 7.28 (m, 5H), 5.12 (s, 2H), 5.05 (br s, 1H), 3.70 – 3.58 (m, 1H), 3.53 (dd, J = 10.3, 4.5 Hz, 2H), 3.33 (dd, J = 10.2, 6.0 Hz, 2H).

^{13}C NMR (151 MHz, $CDCl_3$) δ 155.1, 136.1, 128.8, 128.5, 128.4, 67.5, 51.1, 10.4.

HRMS (EI-TOF): calc'd for $C_{11}H_{13}I_2N_1O_2$ $[M+Na]^+$: 467,8928, found: 467,8925.

Compound 28

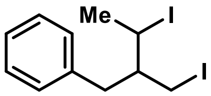

The corresponding diol was synthesized according to the literature.[5] Following **the above Procedure** on 2.5 mmol scale. Purification by flash column chromatography (hexanes to 4:1 hexanes:EtOAc) afforded 550 mg (55 %, d.r. 3:2) of the title compound **28**.

Physical State: yellowish oil.

R_f = 0.5 (hexanes).

^{1}H NMR (400 MHz, CDCl$_3$) δ 7.37 – 7.19 (m, 5H), 4.28 – 4.15 (m, 1H), 3.36 (dd, *J* = 10.2, 4.0 Hz, 1H), 3.20 (dd, *J* = 10.2, 7.1 Hz, 1H), 2.89 (dd, *J* = 13.7, 6.7 Hz, 1H), 2.76 (dd, *J* = 13.7, 7.5 Hz, 1H), 1.99 (d, *J* = 7.0 Hz, 3H), 1.50 – 1.43 (m, 1H). (major)

^{13}C NMR (101 MHz, CDCl$_3$) δ 139.1, 129.1, 128.8, 126.8, 51.0, 38.8, 34.5, 26.4, 12.7. (major)

^{1}H NMR (400 MHz, CDCl$_3$) δ 7.38 – 7.19 (m, 5H), 4.61 – 4.52 (m, 1H), 3.26 (dd, *J* = 10.2, 4.4 Hz, 1H), 3.14 – 3.05 (m, 2H), 2.50 (dd, *J* = 14.1, 9.5 Hz, 1H), 2.04 (d, *J* = 7.1 Hz, 3H), 1.40 (ddd, *J* = 9.5, 7.6, 4.4 Hz, 1H). (minor)

^{13}C NMR (101 MHz, CDCl$_3$) δ 139.0, 129.2, 128.9, 126.8, 50.1, 39.4, 36.0, 26.5, 12.1. (minor)

HRMS (EI-TOF): calc'd for $C_{11}H_{14}I_2$ $[M]^+$: 399.9179, found: 399.9178.

Compound SI-3

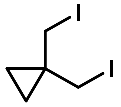

Following **the above Procedure** on 2.0 mmol scale. Purification by flash column chromatography (50:1 pentane:EtOAc) afforded 521 mg (81 %) of the title compound **SI-3**.

Physical State: dark brown oil.

R_f = 0.58 (20:1 pentane:EtOAc).

^{1}H NMR (300 MHz, CDCl$_3$) δ 3.35 (s, 4H), 1.03 (s, 4H).

^{13}C NMR (75 MHz, CDCl$_3$) δ 25.9, 22.4, 18.4.

Spectral data is in accordance with previous report.[6]

5 Unsuccessful substrates

Unsuccessful alkenes:

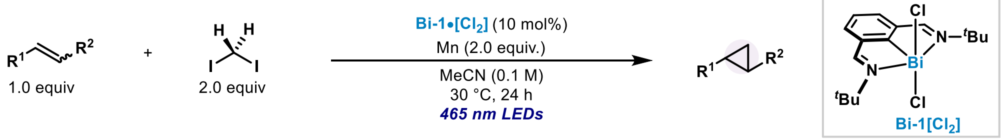

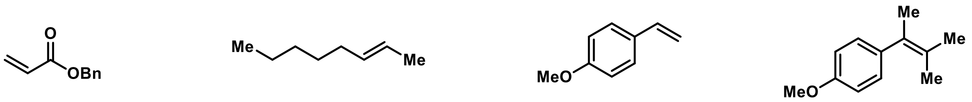

SI-4, trace
SM polymerization

SI-5, trace
SM polymerization

SI-6, trace
SM polymerization

SI-7, 10% ^{1}HNMR yield
SM remained

Intramolecular cyclization:

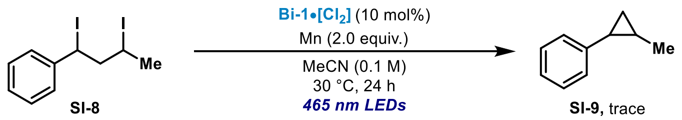

SI-9, trace

Note: The GC-MS only shows trace amount of product, the side reaction may be polymerization..

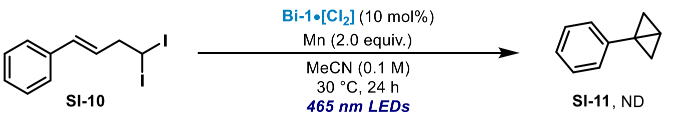

SI-11, ND

Note: The fused bis-cycopropane was not detected; the terminal C–I bonds were reduced to the dehalogenated product.

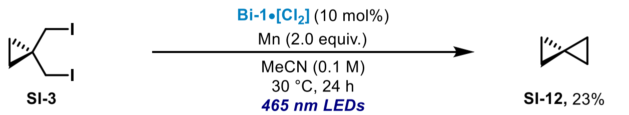

SI-12, 23%

Note: ^{1}H NMR yield using 1,3,5-trimethoxybenzene as the internal standard, The low yield observed is due to formation of competing ring-open by-products, along with unreacted starting material.

Unsuccessful gem-diiodoalkanes:

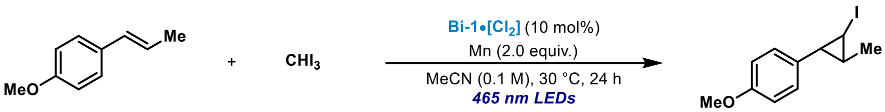

SI-13, ND
SM polymerization

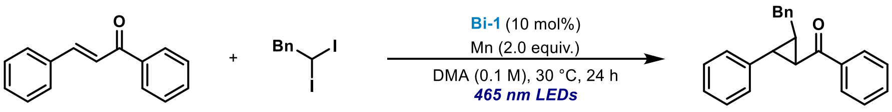

SI-15, 20%, messy

Note: The GC-MS shows 5 different peaks (peak 2,3,4,5,6) with the same MS of the products.

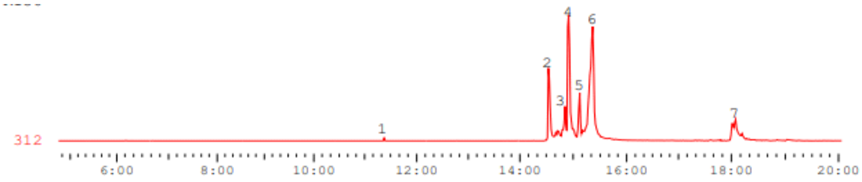

6 General procedure for Bi-catalyzed reductive cyclopropanation with light (General Procedure A)

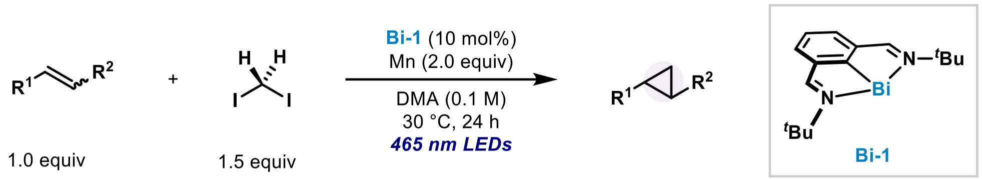

A culture tube with a Teflon screw-cap equipped with a Teflon-coated stir bar was used. The culture tube was brought into an argon-filled glovebox, alkenes (0.10 mmol, 1.0 equiv, *if solid*), **Bi-1** (0.010 mmol, 10 mol%) and Mn (0.20 mmol, 2.0 equiv) were introduced into the culture tube. Then, outside the glovebox, DMA (1.0 mL, 0.10 M) was added using a syringe, alkenes (0.10 mmol, 1.0 equiv, *if liquid*) and CH_2I_2 (0.15 mmol, 1.5 equiv) was added using microsyringes. The reaction tube was sealed with parafilm and placed it into a 465 nm blue LEDs (LEDXON® 24 V LED band) reactor with a cooling fan to keep the temperature 30 °C. After 24 h, the mixture was diluted with MTBE (approximately 4 mL), washed with brine (approximately 4 mL), and dried over Na_2SO_4. Upon filtration, the organic layer was concentrated under reduced pressure (water bath at 40 °C) and purified by flash column chromatography (silica gel) or preparative TLC (pTLC) to afford the desired product.

7 General procedure for Bi-catalyzed reductive cyclopropanation with light (General Procedure B)

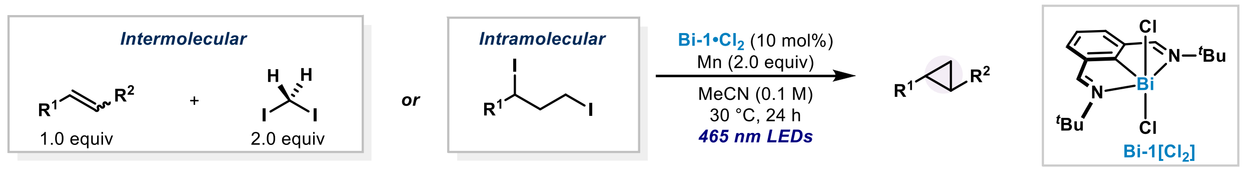

A culture tube with a Teflon screw-cap equipped with a Teflon-coated stir bar was used. The culture tube was brought into an argon-filled glovebox, alkenes (0.10 mmol, 1.0 equiv, *if solid*), **Bi-1·[Cl₂]** (0.010 mmol, 10 mol%) and Mn (0.20 mmol, 2.0 equiv) were introduced into the culture tube. Then, outside the glovebox, MeCN (1.0 mL, 0.10 M) was added using a syringe, alkenes (0.10 mmol, 1.0 equiv, *if liquid*) and CH_2I_2 (0.20 mmol, 2.0 equiv) was added using microsyringes.

The reaction tube was sealed with parafilm and placed it into a 465 nm blue LEDs (LEDXON® 24 V LED band) reactor with a cooling fan to keep the temperature 30 °C. After 24 h, the mixture was diluted with MTBE (approximately 4 mL), washed with brine (approximately 4 mL), and dried over Na_2SO_4. Upon filtration, the organic layer was concentrated under reduced pressure (water bath at 40 °C) and purified by flash column chromatography (silica gel) or preparative TLC (pTLC) to afford the desired product.

8 Characterization Data

Compound 3

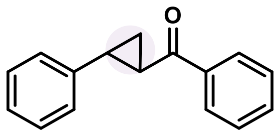

Following **General Procedure A** on 0.10 mmol scale. Purification by pTLC (20:1 hexanes:EtOAc) afforded 15.5 mg (70 %) of the title compound **3**.

Physical State: colorless oil.

R_f = 0.35 (20:1 hexanes:EtOAc).

^{1}H NMR (300 MHz, $CDCl_3$) δ 8.03 – 7.96 (m, 2H), 7.62 – 7.51 (m, 1H), 7.50 – 7.39 (m, 2H), 7.35 – 7.27 (m, 2H), 7.27 – 7.14 (m, 3H), 2.90 (ddd, *J* = 8.0, 5.3, 4.0 Hz, 1H), 2.70 (ddd, *J* = 8.9, 6.6, 4.0 Hz, 1H), 1.93 (ddd, *J* = 9.2, 5.3, 4.1 Hz, 1H), 1.56 (ddd, *J* = 8.0, 6.6, 4.1 Hz, 1H).

^{13}C NMR (75 MHz, $CDCl_3$) δ 198.7, 140.6, 137.9, 133.1, 128.7, 128.3, 126.7, 126.4, 30.2, 29.4, 19.4. (*one carbon is missing due to overlapping*)

HRMS (EI-TOF): calc'd for $C_{16}H_{14}O_1$ $[M]^+$: 222.1039, found: 222.1038.

Compound 4

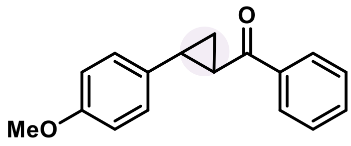

Following **General Procedure A** on 0.10 mmol scale. Purification by pTLC (20:1 hexanes:EtOAc) afforded 18.4 mg (73 %) of the title compound **4**.

Physical State: colorless oil.

R_f = 0.21 (20:1 hexanes:EtOAc).

^{1}H NMR (300 MHz, CDCl$_3$) δ 8.04 – 7.95 (m, 2H), 7.62 – 7.52 (m, 1H), 7.51 – 7.42 (m, 2H), 7.17 – 7.06 (m, 2H), 6.92 – 6.80 (m, 2H), 3.80 (s, 3H), 2.83 (ddd, *J* = 8.0, 5.2, 4.0 Hz, 1H), 2.73 – 2.60 (m, 1H), 1.90 (ddd, *J* = 9.2, 5.3, 4.1 Hz, 1H), 1.52 (ddd, *J* = 8.0, 6.7, 4.1 Hz, 1H).

^{13}C NMR (75 MHz, CDCl$_3$) δ 198.8, 158.6, 138.0, 133.0, 132.6, 128.7, 128.2, 127.5, 114.2, 55.5, 29.8, 29.4, 19.1.

HRMS (EI-TOF): calc'd for $C_{17}H_{16}O_2$ $[M]^+$: 252.1145, found: 252.1147.

Compound 5

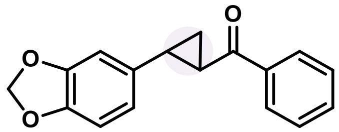

Following **General Procedure A** on 0.10 mmol scale. Purification by pTLC (20:1 hexanes:EtOAc) afforded 20.2 mg (76%) of the title compound **5**.

Physical State: colorless oil.

R_f = 0.19 (20:1 hexanes:EtOAc).

^{1}H NMR (300 MHz, CDCl$_3$) δ 8.14 – 7.93 (m, 1H), 7.66 – 7.52 (m, 1H), 7.47 (dd, *J* = 8.2, 6.6 Hz, 1H), 6.75 (d, *J* = 7.9 Hz, 1H), 6.71 – 6.60 (m, 2H), 5.94 (s, 2H), 2.82 (ddd, *J* = 8.1, 5.3, 4.0 Hz, 1H), 2.64 (ddd, *J* = 9.0, 6.6, 4.0 Hz, 1H), 1.88 (ddd, *J* = 9.2, 5.3, 4.1 Hz, 1H), 1.48 (ddd, *J* = 8.1, 6.6, 4.1 Hz, 1H).

^{13}C NMR (75 MHz, CDCl$_3$) δ 198.7, 148.0, 146.5, 137.9, 134.5, 133.0, 128.7, 128.2, 119.9, 108.4, 106.8, 101.2, 30.2, 29.3, 19.1.

HRMS (EI-TOF): calc'd for $C_{17}H_{14}O_3$ $[M]^+$: 266.0937, found: 266.0942.

Compound 6

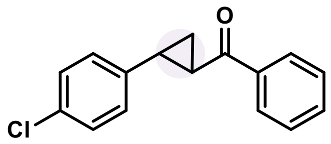

Following **General Procedure A** on 0.10 mmol scale. Purification by pTLC (20:1 hexanes:EtOAc) afforded 17.6 mg (69%) of the title compound **6**.

Physical State: colorless oil.

R_f = 0.29 (20:1 hexanes:EtOAc).

^{1}H NMR (300 MHz, $CDCl_3$) δ 7.99 – 7.92 (m, 2H), 7.57 – 7.50 (m, 1H), 7.49 – 7.39 (m, 2H), 7.29 – 7.20 (m, 2H), 7.13 – 7.04 (m, 2H), 2.83 (ddd, *J* = 8.0, 5.3, 4.0 Hz, 1H), 2.64 (ddd, *J* = 8.9, 6.6, 4.0 Hz, 1H), 1.89 (ddd, *J* = 9.2, 5.3, 4.2 Hz, 1H), 1.49 (ddd, *J* = 8.1, 6.6, 4.2 Hz, 1H).

^{13}C NMR (75 MHz, $CDCl_3$) δ 198.4, 139.2, 137.7, 133.2, 132.5, 128.8, 128.8, 128.2, 127.7, 29.4, 29.3, 19.3.

HRMS (EI-TOF): calc'd for $C_{16}H_{13}O_1Cl_1$ $[M]^+$: 256.0649, found: 256.0651.

Compound 7

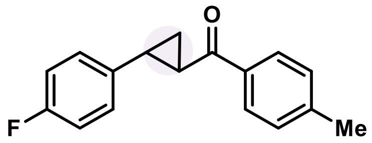

Following **General Procedure A** on 0.10 mmol scale. Purification by pTLC (96:4 hexanes:EtOAc) afforded 16.0 mg (63%) of the title compound **7**.

Physical State: colorless oil.

R_f = 0.30 (20:1 hexanes:EtOAc).

^{1}H NMR (300 MHz, $CDCl_3$) δ 7.89 (d, *J* = 8.2 Hz, 2H), 7.30 – 7.23 (m, 2H), 7.17 – 7.09 (m, 2H), 7.03 – 6.94 (m, 2H), 2.88 – 2.75 (m, 1H), 2.67 (ddd, *J* = 9.0, 6.6, 4.0 Hz, 1H), 2.42 (s, 3H), 1.88 (ddd, *J* = 9.2, 5.3, 4.2 Hz, 1H), 1.48 (ddd, *J* = 8.1, 6.6, 4.2 Hz, 1H).

^{13}C NMR (75 MHz, CDCl$_3$) δ 198.1, 163.4 (d, *J* = 245.1 Hz), 144.0, 136.4, 135.3, 129.4, 128.4, 128.0 (d, *J* = 7.9 Hz), 115.5 (d, *J* = 21.5 Hz), 29.1 (d, *J* = 2.0 Hz), 21.8, 19.1, 1.2.

^{19}F NMR (282 MHz, CDCl$_3$) δ –116.27.

HRMS (EI-TOF): calc'd for $C_{17}H_{15}O_1F_1$ $[M]^+$: 254.1101, found: 254.1105.

Compound 8

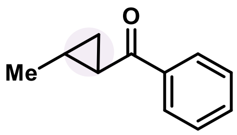

Following **General Procedure A** on 0.10 mmol scale. Purification by pTLC (20:1 hexanes:EtOAc) afforded 9.1 mg (57%) of the title compound **8**.

Physical State: colorless oil.

R_f = 0.38 (20:1 hexanes:EtOAc).

^{1}H NMR (600 MHz, CDCl$_3$) δ 8.01 – 7.97 (m, 2H), 7.58 – 7.53 (m, 1H), 7.50 – 7.44 (m, 2H), 2.40 (ddd, *J* = 7.8, 4.6, 3.9 Hz, 1H), 1.67 – 1.57 (m, 1H), 1.49 (ddd, *J* = 8.6, 4.6, 3.5 Hz, 1H), 1.23 (d, *J* = 6.0 Hz, 3H), 0.90 (ddd, *J* = 7.9, 6.5, 3.5 Hz, 1H).

^{13}C NMR (151 MHz, CDCl$_3$) δ 200.3, 138.3, 132.7, 128.6, 128.1, 26.6, 21.5, 20.3, 18.5.
HRMS (EI-TOF): calc'd for $C_{11}H_{12}O_1$ $[M]^+$: 160.0883, found: 160.0884.

Compound 9

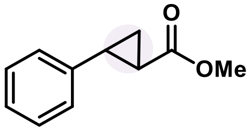

Following **General Procedure A** on 0.10 mmol scale. Purification by pTLC (20:1 hexanes:EtOAc) afforded 8.4 mg (48%) of the title compound **9**.

Physical State: colorless oil.

R_f = 0.25 (20:1 hexanes:EtOAc).

^{1}H NMR (300 MHz, CDCl$_3$) δ 7.30 – 7.23 (m, 2H), 7.22 – 7.15 (m, 1H), 7.11 – 7.06 (m, 2H), 3.70 (s, 3H), 2.52 (ddd, *J* = 9.2, 6.5, 4.1 Hz, 1H), 1.89 (ddd, *J* = 8.4, 5.3, 4.2 Hz, 1H), 1.64 – 1.53 (m, 1H), 1.31 (ddd, *J* = 8.4, 6.5, 4.6 Hz, 1H).

^{13}C NMR (75 MHz, CDCl$_3$) δ 174.0, 140.1, 128.6, 126.7, 126.4, 52.1, 26.4, 24.1, 17.2.

HRMS (EI-TOF): calc'd for $C_{11}H_{12}O_2$ $[M]^+$: 176.0832, found: 176.0834.

Compound 10

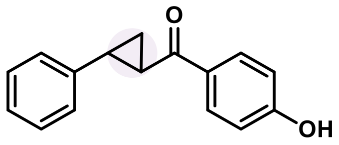

Following **General Procedure A** on 0.10 mmol scale. Purification by pTLC (4:1 hexanes:EtOAc) afforded 13.8 mg (58%) of the title compound **10**.

Physical State: white solid.

m.p.: 156 – 158 °C.

R_f = 0.15 (20:1 hexanes:EtOAc).

^{1}H NMR (300 MHz, CDCl$_3$) δ 7.93 – 7.81 (m, 2H), 7.30 – 7.06 (m, 5H), 6.88 – 6.75 (m, 2H), 6.15 (br s, 1H), 2.84 – 2.72 (m, 1H), 2.67 – 2.56 (m, 1H), 1.90 – 1.77 (m, 1H), 1.51 – 1.40 (m, 1H).

^{13}C NMR (75 MHz, CDCl$_3$) δ 197.7, 160.5, 140.7, 130.9, 130.8, 128.7, 126.7, 126.4, 115.6, 29.9, 29.2, 19.2.

HRMS (EI-TOF): calc'd for $C_{16}H_{14}O_2$ $[M]^+$: 238.0988, found: 238.0990.

Compound 11

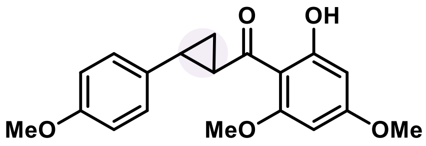

Following **General Procedure A** on 0.10 mmol scale. Purification by pTLC (20:1 hexanes:EtOAc) afforded 17.7 mg (54%) of the title compound **11**.

Physical State: white solid.

m.p.: 70 – 72 °C.

R_f = 0.73 (1:1 hexanes:EtOAc).

^{1}H NMR (300 MHz, CDCl$_3$) δ 13.79 (s, 1H), 7.15 – 7.04 (m, 2H), 6.92 – 6.77 (m, 2H), 6.07 (d, *J* = 2.4 Hz, 1H), 5.91 (d, *J* = 2.4 Hz, 1H), 3.81 (s, 3H), 3.80 (s, 3H), 3.66 (s, 3H), 3.30 (ddd, *J* = 7.9, 5.6, 4.1 Hz, 1H), 2.59 (ddd, *J* = 9.0, 6.6, 4.1 Hz, 1H), 1.99 – 1.87 (m, 1H), 1.36 (ddd, *J* = 7.9, 6.5, 4.0 Hz, 1H).

^{13}C NMR (75 MHz, CDCl$_3$) δ 202.5, 166.9, 165.9, 162.9, 158.4, 133.4, 127.6, 113.9, 106.6, 93.7, 91.2, 55.7, 55.7, 55.5, 32.9, 30.3, 18.9.

HRMS (EI-TOF): calc'd for $C_{19}H_{20}O_5$ $[M]^+$: 328.1305, found: 328.1309.

Compound 12

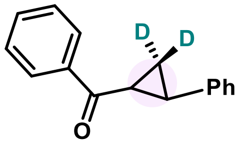

Following **General Procedure A** on 0.10 mmol scale. Purification by pTLC (20:1 hexanes:EtOAc) afforded 16.1 mg (72%) of the title compound **12**.

Physical State: colorless oil.

R_f = 0.35 (20:1 hexanes:EtOAc).

^{1}H NMR (300 MHz, CDCl$_3$) δ 8.04 – 7.96 (m, 2H), 7.62 – 7.52 (m, 1H), 7.51 – 7.41 (m, 2H), 7.36 – 7.28 (m, 2H), 7.27 – 7.15 (m, 3H), 2.90 (d, *J* = 4.0 Hz, 1H), 2.70 (d, *J* = 4.0 Hz, 1H).

^{13}C NMR (151 MHz, CDCl$_3$) δ 198.7, 140.6, 137.9, 133.1, 128.7, 128.3, 126.7, 126.4, 30.0, 29.3, 19.0, 18.8, 18.6. (*one carbon is missing due to overlapping*)

HRMS (EI-TOF): calc'd for $C_{16}H_{12}O_1D_2$ $[M]^+$: 224.1165, found: 224.1166.

Compound 13

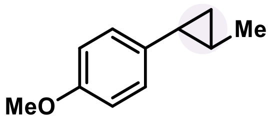

Following **General Procedure B** on 0.10 mmol scale by using anethole (*E/Z > 20:1*) as the substrate. Purification by pTLC (20:1 hexanes:EtOAc) afforded 14.9 mg (92%) of the title compound **13**.

Following **General Procedure B** on 1.0 mmol scale by using anethole (*E/Z > 20:1*) as the substrate and 1 mol% Bi-1·Cl_2 as the catalyst. Purification by flash column chromatography (50:1 hexanes:EtOAc) afforded 125 mg (77%) of the title compound **13**.

Physical State: colorless oil.

R_f = 0.26 (20:1 hexanes:EtOAc).

^{1}H NMR (300 MHz, CDCl$_3$) δ 6.97 (d, *J* = 8.6 Hz, 2H), 6.80 (d, *J* = 8.7 Hz, 2H), 3.78 (s, 3H), 1.54 – 1.48 (m, 1H), 1.17 (d, *J* = 5.9 Hz, 3H), 1.02 – 0.91 (m, 1H), 0.80 (dt, *J* = 8.5, 4.8 Hz, 1H), 0.67 (dt, *J* = 8.8, 5.0 Hz, 1H).

^{13}C NMR (75 MHz, CDCl$_3$) δ 157.6, 136.2, 126.7, 113.9, 55.5, 23.7, 19.2, 17.4, 17.1.

HRMS (EI-TOF): calc'd for $C_{11}H_{14}O_1$ $[M]^+$: 162.1039, found: 162.1039.

Compound 14

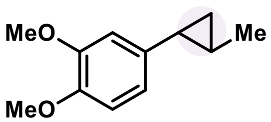

Following **General Procedure B** on 0.10 mmol scale by using the corresponding alkene (*E/Z > 20:1*) as the substrate. Purification by pTLC (20:1 hexanes:EtOAc) afforded 16.1 mg (84%) of the title compound **14**.

Physical State: colorless oil.

R_f = 0.15 (20:1 hexanes:EtOAc).

^{1}H NMR (300 MHz, CDCl$_3$) δ 6.80 – 6.72 (m, 1H), 6.62 – 6.54 (m, 2H), 3.87 (s, 3H), 3.84 (s, 3H), 1.60 – 1.48 (m, 1H), 1.18 (d, *J* = 5.9 Hz, 3H), 1.05 – 0.94 (m, 1H), 0.88 – 0.77 (m, 1H), 0.72 – 0.64 (m, 1H).

^{13}C NMR (75 MHz, CDCl$_3$) δ 149.0, 147.0, 136.8, 117.4, 111.5, 109.6, 56.2, 55.9, 24.1, 19.2, 17.5, 17.1.

HRMS (EI-TOF): calc'd for $C_{12}H_{16}O_2$ $[M]^+$: 192.1145, found: 192.1148.

Compound 15

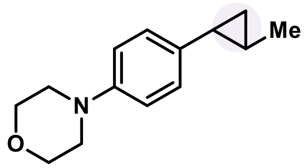

Following **General Procedure B** on 0.10 mmol scale by using the corresponding alkene (*E/Z = 1:4*) as the substrate. Purification by pTLC (20:1 hexanes:EtOAc) afforded 14.1 mg (65%) of the title compound **15**.

Physical State: dark yellow solid.

m.p.: 57 – 59 °C.

R_f = 0.60 (4:1 hexanes:EtOAc).

^{1}H NMR (300 MHz, CDCl$_3$) δ 7.02 – 6.93 (m, 2H), 6.86 – 6.79 (m, 2H), 3.90 – 3.81 (m, 4H), 3.13 – 3.07 (m, 4H), 1.55 – 1.46 (m, 1H), 1.17 (d, *J* = 5.9 Hz, 3H), 1.04 – 0.92 (m, 1H), 0.85 – 0.77 (m, 1H), 0.71 – 0.60 (m, 1H).

^{13}C NMR (75 MHz, CDCl$_3$) δ 149.3, 135.9, 126.5, 116.2, 67.1, 50.1, 23.7, 19.2, 17.4, 17.1.

HRMS (ESI-TOF): calc'd for $C_{14}H_{19}N_1O_1$ $[M]^+$: 217.1461, found: 217.1464.

Compound 16

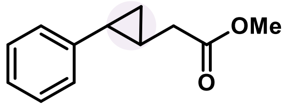

Following **General Procedure B** on 0.10 mmol scale by using the corresponding alkene (*E/Z > 20:1*) as the substrate. Purification by pTLC (20:1 hexanes:EtOAc) afforded 14.8 mg (78%) of the title compound **16**.

Physical State: colorless oil.

R_f = 0.25 (20:1 hexanes:EtOAc).

^{1}H NMR (300 MHz, CDCl$_3$) δ 7.25 – 6.94 (m, 5H), 3.63 (s, 3H), 2.39 (dd, *J* = 15.8, 6.9 Hz, 1H), 2.28 (dd, *J* = 15.8, 7.2 Hz, 1H), 1.69 (dt, *J* = 9.2, 4.9 Hz, 1H), 1.40 – 1.24 (m, 1H), 0.93 (dt, *J* = 8.5, 5.2 Hz, 1H), 0.79 (dt, *J* = 8.7, 5.3 Hz, 1H).

^{13}C NMR (75 MHz, CDCl$_3$) δ 173.3, 142.7, 128.4, 126.2, 125.8, 51.8, 38.9, 23.0, 18.6, 15.5.

HRMS (EI-TOF): calc'd for $C_{12}H_{14}O_2$ $[M]^+$: 190.0988, found: 190.0989.

Compound 17

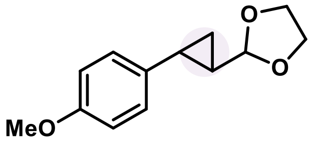

Following **General Procedure B** on 0.10 mmol scale by using the corresponding alkene (*E/Z = 1.8:1*) as the substrate. Purification by pTLC (20:1 hexanes:EtOAc) afforded 13.8 mg (63%) of the title compound **17**.

Physical State: colorless oil.

R_f = 0.50 (4:1 hexanes:EtOAc).

^{1}H NMR (300 MHz, $CDCl_3$) δ 7.09 – 6.98 (m, 2H), 6.85 – 6.75 (m, 2H), 4.69 (d, *J* = 5.4 Hz, 1H), 4.06 – 3.97 (m, 2H), 3.91 – 3.83 (m, 2H), 3.77 (s, 3H), 1.99 (dt, *J* = 9.4, 5.1 Hz, 1H), 1.44 – 1.32 (m, 1H), 1.05 (dt, *J* = 9.0, 5.3 Hz, 1H), 0.91 (ddd, *J* = 8.7, 5.6, 5.0 Hz, 1H).

^{13}C NMR (75 MHz, $CDCl_3$) δ 158.0, 133.9, 127.5, 114.0, 106.2, 65.2, 65.2, 55.5, 24.4, 18.9, 11.2.

HRMS (EI-TOF): calc'd for $C_{13}H_{16}O_3$ $[M]^+$: 220.1094, found: 220.1094.

Compound 18

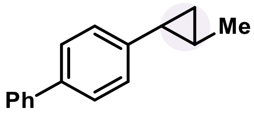

Following **General Procedure B** on 0.10 mmol scale by using the corresponding alkene (*E/Z = 1:3*) as the substrate. Purification by pTLC (20:1 hexanes:EtOAc) afforded 14.3 mg (69%) of the title compound **18**.

Physical State: colorless oil.

R_f = 0.53 (20:1 hexanes:EtOAc).

^{1}H NMR (300 MHz, $CDCl_3$) δ7.65 – 7.55 (m, 2H), 7.54 – 7.39 (m, 4H), 7.37 – 7.29 (m, 1H), 7.13 (d, *J* = 8.3 Hz, 2H), 1.68 – 1.58 (m, 1H), 1.23 (d, *J* = 5.7 Hz, 3H), 1.17 – 1.05 (m, 1H), 1.00 – 0.90 (m, 1H), 0.85 – 0.75 (m, 1H).

^{13}C NMR (75 MHz, $CDCl_3$) δ143.5, 141.3, 138.3, 128.8, 127.1, 127.1, 126.0, 24.2, 19.2, 18.3, 17.9. (*one carbon is missing due to overlapping*)

HRMS (EI-TOF): calc'd for $C_{16}H_{16}$ $[M]^+$: 208.1247, found: 208.1278.

Compound 19

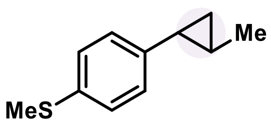

Following **General Procedure B** on 0.10 mmol scale by using the corresponding alkene *(E/Z > 20:1)* as the substrate. Purification by pTLC (20:1 hexanes:EtOAc) afforded 12.1 mg (68%) of the title compound **19**.

Physical State: colorless oil.

R_f = 0.38 (20:1 hexanes:EtOAc).

^{1}H NMR (300 MHz, $CDCl_3$) δ 7.22 – 7.13 (m, 2H), 7.01 – 6.92 (m, 2H), 2.46 (s, 3H), 1.58 – 1.49 (m, 1H), 1.18 (d, *J* = 5.8 Hz, 3H), 1.09 – 0.96 (m, 1H), 0.90 – 0.80 (m, 1H), 0.77 – 0.66 (m, 1H).

^{13}C NMR (75 MHz, $CDCl_3$) δ 141.6, 134.5, 127.7, 126.2, 24.1, 19.2, 18.1, 17.7, 16.9.

HRMS (EI-TOF): calc'd for $C_{11}H_{14}S_1$ $[M]^+$: 178.0811, found: 178.0813.

Compound 20

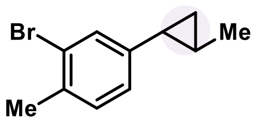

Following **General Procedure B** on 0.10 mmol scale by using the corresponding alkene (*E/Z = 3:5*) as the substrate. Purification by pTLC (20:1 hexanes:EtOAc) afforded 18.6 mg (83%) of the title compound **20**.

Physical State: colorless oil.

R_f = 0.52 (20:1 hexanes:EtOAc).

^{1}H NMR (300 MHz, CDCl$_3$) δ 7.21 (d, *J* = 1.9 Hz, 1H), 7.08 (d, *J* = 7.8 Hz, 1H), 6.87 (dd, *J* = 7.8, 1.9 Hz, 1H), 2.34 (s, 3H), 1.56 – 1.46 (m, 1H), 1.17 (d, *J* = 5.8 Hz, 3H), 1.07 – 0.95 (m, 1H), 0.90 – 0.79 (m, 1H), 0.77 – 0.66 (m, 1H).

^{13}C NMR (75 MHz, CDCl$_3$) δ 143.8, 134.5, 130.6, 129.5, 124.9, 124.7, 23.7, 22.5, 19.1, 18.1, 17.6.

HRMS (EI-TOF): calc'd for $C_{11}H_{13}Br_1$ $[M]^+$: 224.0195, found: 224.0197.

Compound 21

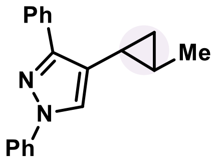

Following **General Procedure B** on 0.10 mmol scale by using the corresponding alkene (*E/Z = 1:4*) as the substrate. Purification by pTLC (20:1 hexanes:EtOAc) afforded 19.7 mg (72%) of the title compound **21**.

Physical State: colorless oil.

R_f = 0.35 (20:1 hexanes:EtOAc).

^{1}H NMR (300 MHz, CDCl$_3$) δ 7.92 – 7.86 (m, 2H), 7.67 – 7.59 (m, 2H), 7.51 (d, *J* = 0.8 Hz, 1H), 7.43 – 7.24 (m, 5H), 7.20 – 7.10 (m, 1H), 1.55 – 1.39 (m, 1H), 1.15 (d, *J* = 5.9 Hz, 3H), 0.98 – 0.85 (m, 1H), 0.74 (ddd, *J* = 8.3, 5.1, 4.3 Hz, 1H), 0.63 (ddd, *J* = 8.4, 5.3, 4.3 Hz, 1H).

^{13}C NMR (75 MHz, CDCl$_3$) δ 151.9, 140.3, 133.9, 129.5, 128.5, 127.8, 127.8, 126.0, 125.3, 124.2, 118.8, 19.0, 16.6, 16.1, 15.1.

HRMS (EI-TOF): calc'd for $C_{19}H_{18}N_2$ $[M]^+$: 274.1464, found: 274.1469.

Compound 22

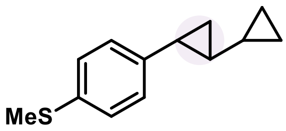

Following **General Procedure B** on 0.10 mmol scale by using the corresponding alkene (*E/Z > 20:1*) as the substrate. Purification by pTLC (20:1 hexanes:EtOAc) afforded 11.2 mg (55%) of the title compound **22**.

Physical State: colorless oil.

R_f = 0.53 (20:1 hexanes:EtOAc).

^{1}H NMR (300 MHz, CDCl$_3$) δ 7.22 – 7.12 (m, 2H), 7.02 – 6.90 (m, 2H), 2.45 (s, 3H), 1.69 – 1.57 (m, 1H), 1.15 – 1.05 (m, 1H), 1.00 – 0.86 (m, 1H), 0.82 – 0.68 (m, 2H), 0.48 – 0.32 (m, 2H), 0.21 – 0.08 (m, 2H).

^{13}C NMR (75 MHz, CDCl$_3$) δ 141.3, 134.6, 127.6, 126.4, 25.5, 21.5, 16.8, 13.9, 12.5, 3.5, 2.7.

HRMS (EI-TOF): calc'd for $C_{13}H_{16}S_1$ $[M]^+$: 204.0967, found: 204.0968

Compound 23

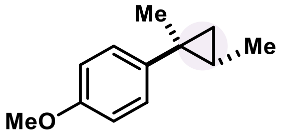

Following **General Procedure B** on 0.10 mmol scale by using the corresponding alkene (*E/Z = 1:1*) as the substrate. Purification by pTLC (20:1 hexanes:EtOAc) afforded 8.1 mg (46%, d.r. = 9:1) of the title compound **23**.

Physical State: colorless oil.

R_f = 0.35 (20:1 hexanes:EtOAc).

^{1}H NMR (600 MHz, CDCl$_3$) δ 7.19 – 7.12 (m, 2H), 6.85 – 6.75 (m, 2H), 3.78 (s, 3H), 1.34 (s, 3H), 1.23 – 1.18 (m, 3H), 1.04 – 0.97 (m, 2H), 0.28 (q, *J* = 1.9 Hz, 1H).

^{13}C NMR (151 MHz, CDCl$_3$) δ 157.5, 141.4, 128.0, 113.7, 55.4, 23.2, 21.8, 20.5, 20.0, 14.2.

HRMS (EI-TOF): calc'd for $C_{12}H_{16}O_1$ $[M]^+$: 176.1196, found: 176.1196.

Compound 29

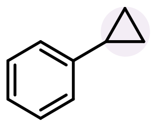

Following **General Procedure B** on 0.10 mmol scale. Due to the compound being volatile, the yield (88 %) was determined by crude ^{1}H NMR using 1,3,5-trimethoxybenzene as the internal standard.

^{1}H NMR (300 MHz, CDCl$_3$) δ 7.36 – 7.24 (m, 2H), 7.23 – 7.16 (m, 1H), 7.15 – 7.09 (m, 2H), 2.05 – 1.85 (m, 1H), 1.08 – 0.90 (m, 2H), 0.86 – 0.67 (m, 2H).

Spectral data is in accordance with previous report.[7]

Compound 30

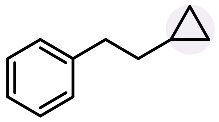

Following **General Procedure B** on 0.10 mmol scale. Purification by pTLC (20:1 hexanes:EtOAc) afforded 13.6 mg (93%) of the title compound **24**.

Physical State: colorless oil.

R_f = 0.48 (hexanes).

^{1}H NMR (300 MHz, CDCl$_3$) δ 7.27 – 7.19 (m, 2H), 7.17 – 7.07 (m, 3H), 2.74 – 2.58 (m, 2H), 1.53 – 1.38 (m, 2H), 0.72 – 0.57 (m, 1H), 0.45 – 0.29 (m, 2H), 0.09 – -0.09 (m, 2H).

^{13}C NMR (75 MHz, CDCl$_3$) δ 142.8, 128.6, 128.4, 125.7, 36.9, 36.2, 10.9, 4.6.

HRMS (EI-TOF): calc'd for $C_{11}H_{14}$ $[M]^+$: 146.1090, found: 146.1092.

Compound 31

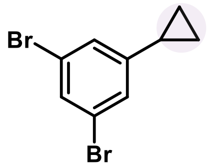

Following **General Procedure B** on 0.10 mmol scale. Purification by pTLC (20:1 hexanes:EtOAc) afforded 22.1 mg (81%) of the title compound **25**.

Physical State: colorless oil.

R_f = 0.45 (hexanes).

^{1}H NMR (300 MHz, CDCl$_3$) δ 7.43 (t, *J* = 1.8 Hz, 1H), 7.13 (dd, *J* = 1.8, 0.5 Hz, 2H), 1.90 – 1.74 (m, 1H), 1.05 – 0.95 (m, 2H), 0.73 – 0.65 (m, 2H).

^{13}C NMR (75 MHz, CDCl$_3$) δ 148.5, 131.1, 127.8, 122.9, 15.2, 9.8.

HRMS (EI-TOF): calc'd for $C_9H_8Br_2$ $[M]^+$: 273.8988, found: 273.8989.

Compound 32

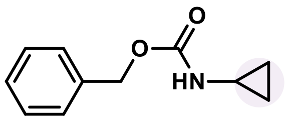

Following **General Procedure B** on 0.10 mmol scale. Purification by pTLC (20:1 hexanes:EtOAc) afforded 17.0 mg (89%) of the title compound **26**.

Physical State: white solid.

m.p.: 51 – 53 °C.

R_f = 0.43 (4:1 hexanes:EtOAc).

^{1}H NMR (300 MHz, CDCl$_3$) δ 7.45 – 7.27 (m, 5H), 5.10 (s, 2H), 4.98 (br s, 1H), 2.72 – 2.51 (m, 1H), 0.76 – 0.67 (m, 2H), 0.56 – 0.47 (m, 2H).

^{13}C NMR (75 MHz, CDCl$_3$) δ 157.2, 136.7, 128.6, 128.2, 127.1, 66.8, 23.3, 7.0.

HRMS (ESI-TOF): calc'd for $C_{11}H_{13}O_2N_1$ $[M+Na]^+$: 214.0838, found: 214.0835.

Compound 33

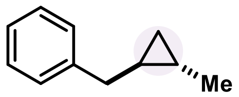

Following **General Procedure B** on 0.10 mmol scale by using MeCN-d_3 as the slovent. Due to the compound being volatile, the yield (81 %, d.r.= 5:1) was determined by crude ^{1}H NMR using 1,3,5-trimethoxybenzene (0.03 mmol) as the internal standard.

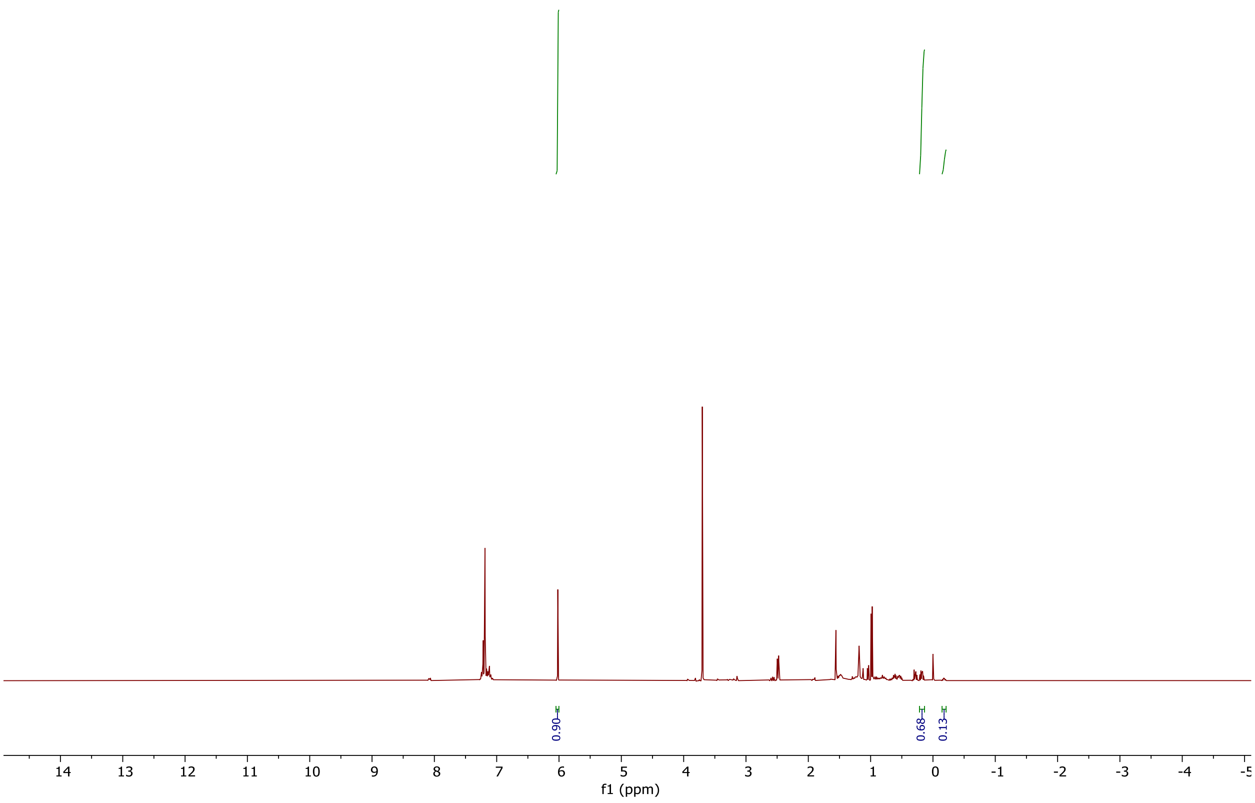

Spectral data is in accordance with previous report.[8]

9 References

[1] a) del Hoyo, A. M.; Herraiz, A. G.; Suero, M. G. A Stereoconvergent Cyclopropanation Reaction of Styrenes. *Angew. Chem., Int. Ed.* **2017,** *56*, 1610–1613; b) Sanford, A. B.; Thane, T. A.; McGinnis, T. M.; Chen, P.-P.; Hong, X.; Jarvo, E. R. Nickel-Catalyzed Alkyl–Alkyl Cross-Electrophile Coupling Reaction of 1,3-Dimesylates for the Synthesis of Alkylcyclopropanes. *J. Am. Chem. Soc.* **2020,** *142*, 5017–5023; c) Gore, V.; Gravel, S.; Cossette, C.; Patel, P.; Chourey, S.; Ye, Q.; Rokach, J.; Powell, W. S. Inhibition of 5-Oxo-6,8,11,14-eicosatetraenoic Acid-Induced Activation of Neutrophils and Eosinophils by Novel Indole OXE Receptor Antagonists. *J. Med. Chem.* **2014,** *57*, 364–377; d) Chen, P.-P.; McGinnis, T. M.; Lin, P. C.; Hong, X.; Jarvo, E. R. A Nickel-Catalyzed Cross-Electrophile Coupling Reaction of 1,3-Dimesylates for Alkylcyclopropane Synthesis: Investigation of Stereochemical Outcomes and Radical Lifetimes. *ACS Catal.* **2023,** *13*, 5472–5481.

[2] Vránová, I.; Jambor, R.; Růžička, A.; Jirásko, R.; Dostál, L. Reactivity of N,C,N-Chelated Antimony(III) and Bismuth(III) Chlorides with Lithium Reagents: Addition vs Substitution. *Organometallics* **2015,** *34*, 534–541.

[3] a) Tsuruta, T.; Spinnato, D.; Moon, H. W.; Leutzsch, M.; Cornella, J. Bi-Catalyzed Trifluoromethylation of C(sp^2)–H Bonds under Light. *J. Am. Chem. Soc.* **2023,** *145*, 25538–25544; b) Lehnherr, D.; Ji, Y.; Neel, A. J.; Cohen, R. D.; Brunskill, A. P. J.; Yang, J.; Reibarkh, M. Discovery of a Photoinduced Dark Catalytic Cycle Using in Situ LED-NMR Spectroscopy. *J. Am. Chem. Soc.* **2018**, *140*, 13843–13853.

[4] del Hoyo, A. M.; Herraiz, A. G.; Suero, M. G. A Stereoconvergent Cyclopropanation Reaction of Styrenes. *Angew. Chem., Int. Ed.* **2017,** *56*, 1610–1613.

[5] Sanford, A. B.; Thane, T. A.; McGinnis, T. M.; Chen, P.-P.; Hong, X.; Jarvo, E. R. Nickel-Catalyzed Alkyl–Alkyl Cross-Electrophile Coupling Reaction of 1,3-Dimesylates for the Synthesis of Alkylcyclopropanes. *J. Am. Chem. Soc.* **2020,** *142*, 5017–5023.

[6] Gore, V.; Gravel, S.; Cossette, C.; Patel, P.; Chourey, S.; Ye, Q.; Rokach, J.; Powell, W. S., Inhibition of 5-Oxo-6,8,11,14-eicosatetraenoic Acid-Induced Activation of Neutrophils and Eosinophils by Novel Indole OXE Receptor Antagonists. *J. Med. Chem.* **2014,** *57*, 364–377.

[7] Li, H.; Breen, C. P.; Seo, H.; Jamison, T. F.; Fang, Y.-Q.; Bio, M. M., Ni-Catalyzed Electrochemical Decarboxylative C–C Couplings in Batch and Continuous Flow. *Org. Lett.* **2018,** *20*, 1338–1341.

[8] Guijarro, D.; Yus, M. J. T., Synthesis of substituted cyclopropanes from 1, 3-diols through the corresponding cyclic sulfates. *Tetrahedron* **1995,** *51*, 11445–11456.

10 NMR Spectra

Compound 24 ^{1}H NMR in $CDCl_3$, 298 K, 300 MHz

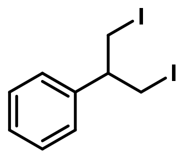

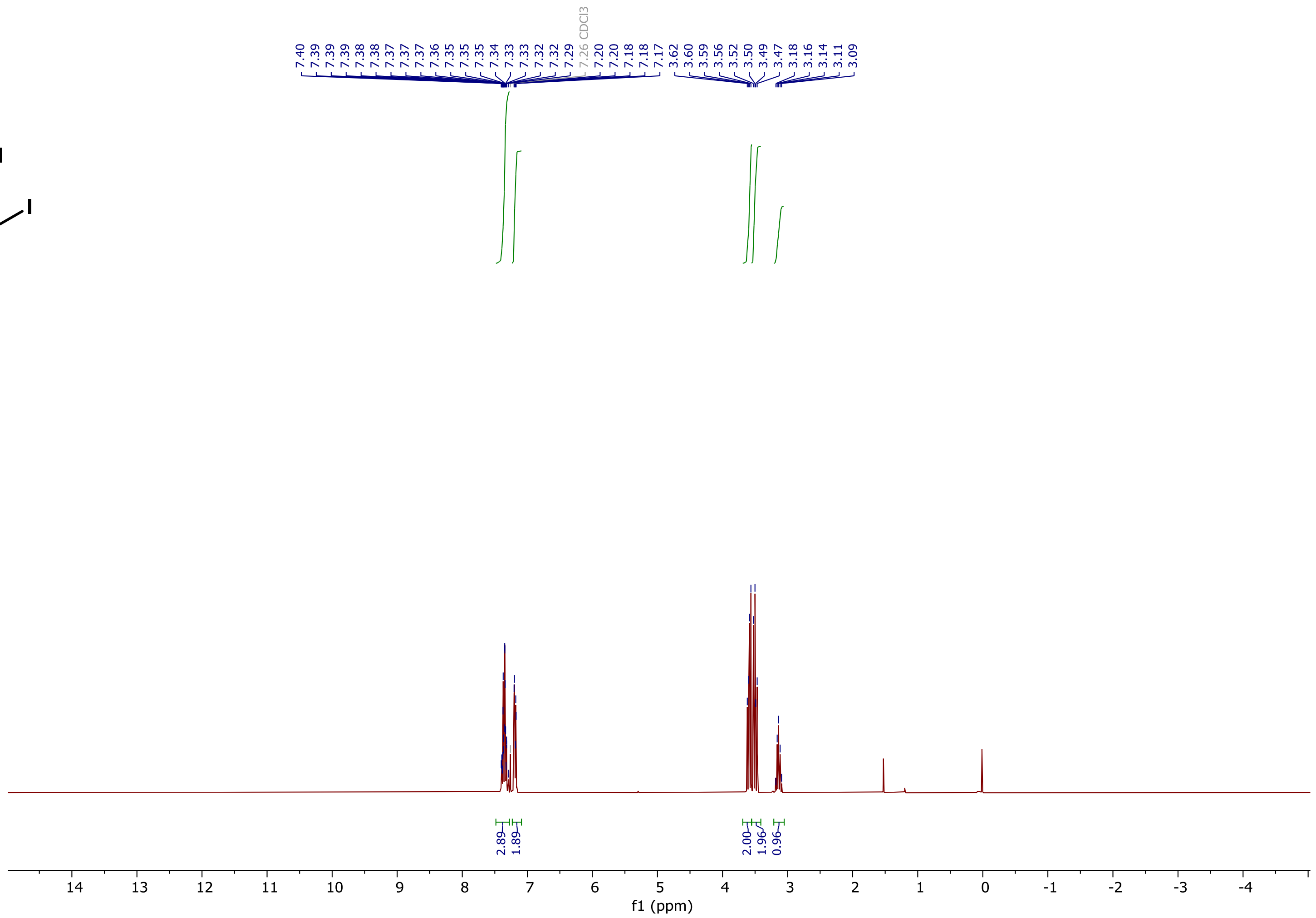

Compound 24 ^{13}C NMR in $CDCl_3$, 298 K, 75 MHz

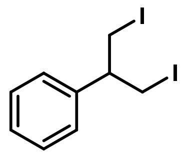

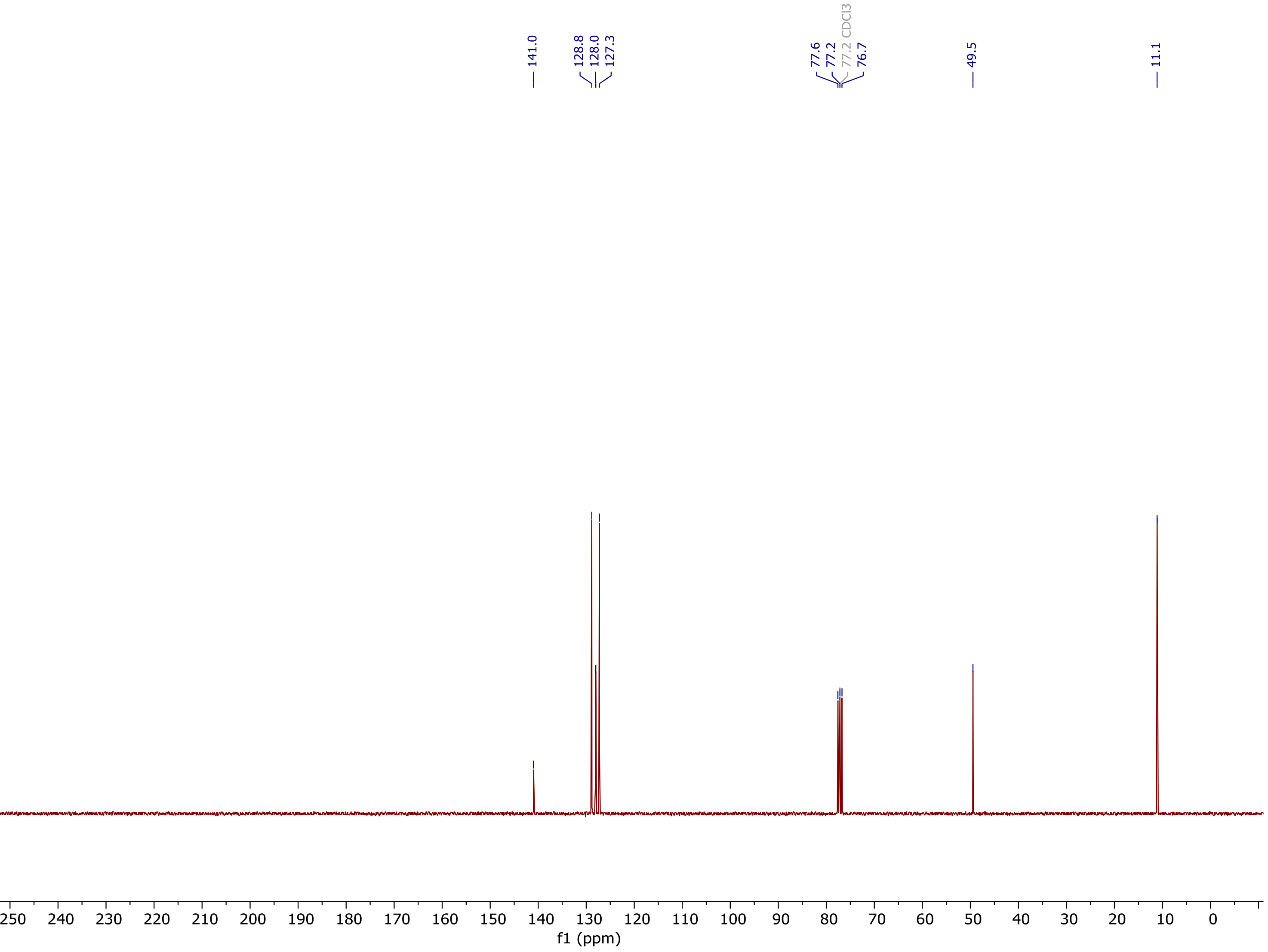

Compound 25 ^{1}H NMR in $CDCl_3$, 298 K, 300 MHz

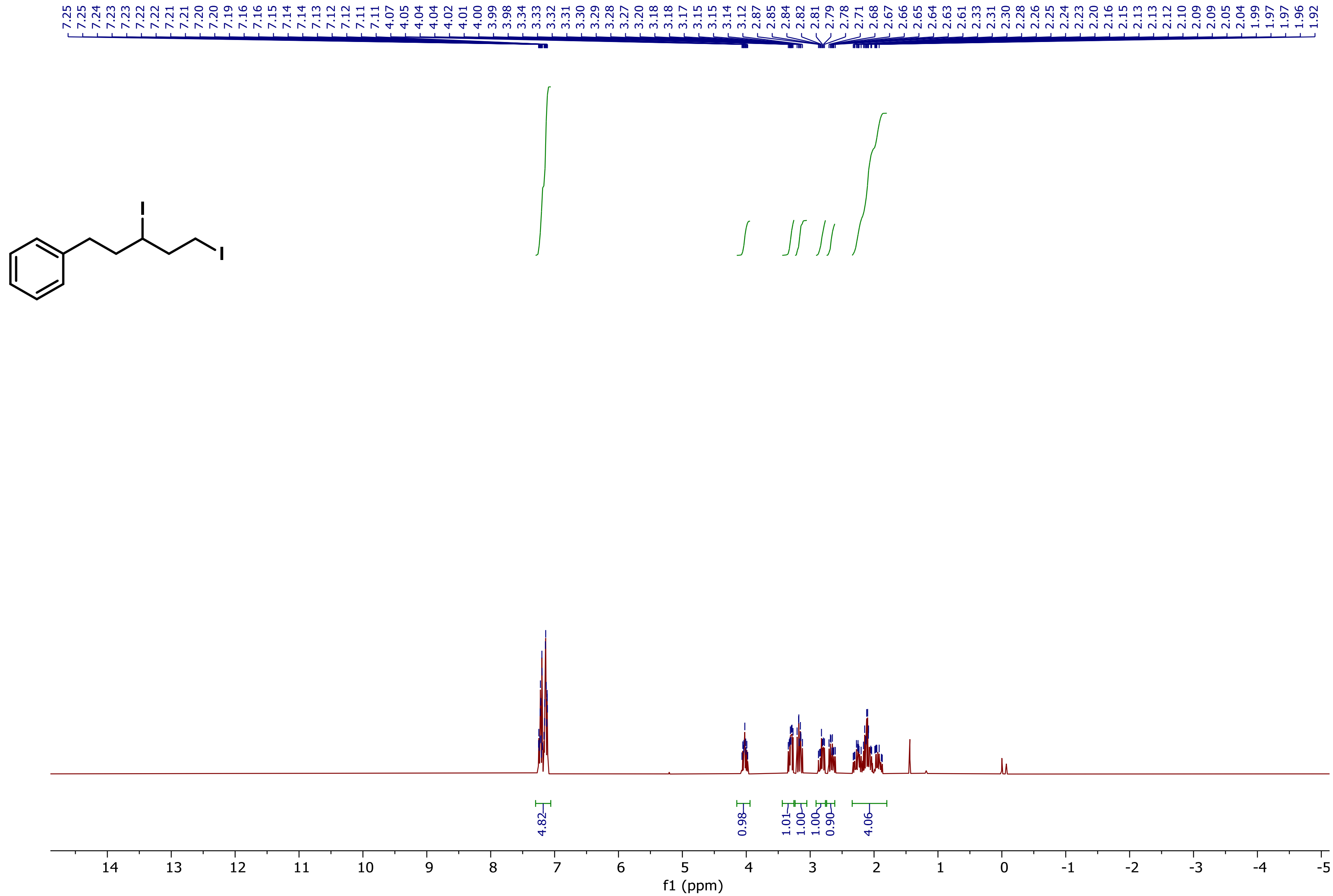

Compound 25 ^{13}C NMR in $CDCl_3$, 298 K, 75 MHz

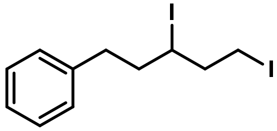

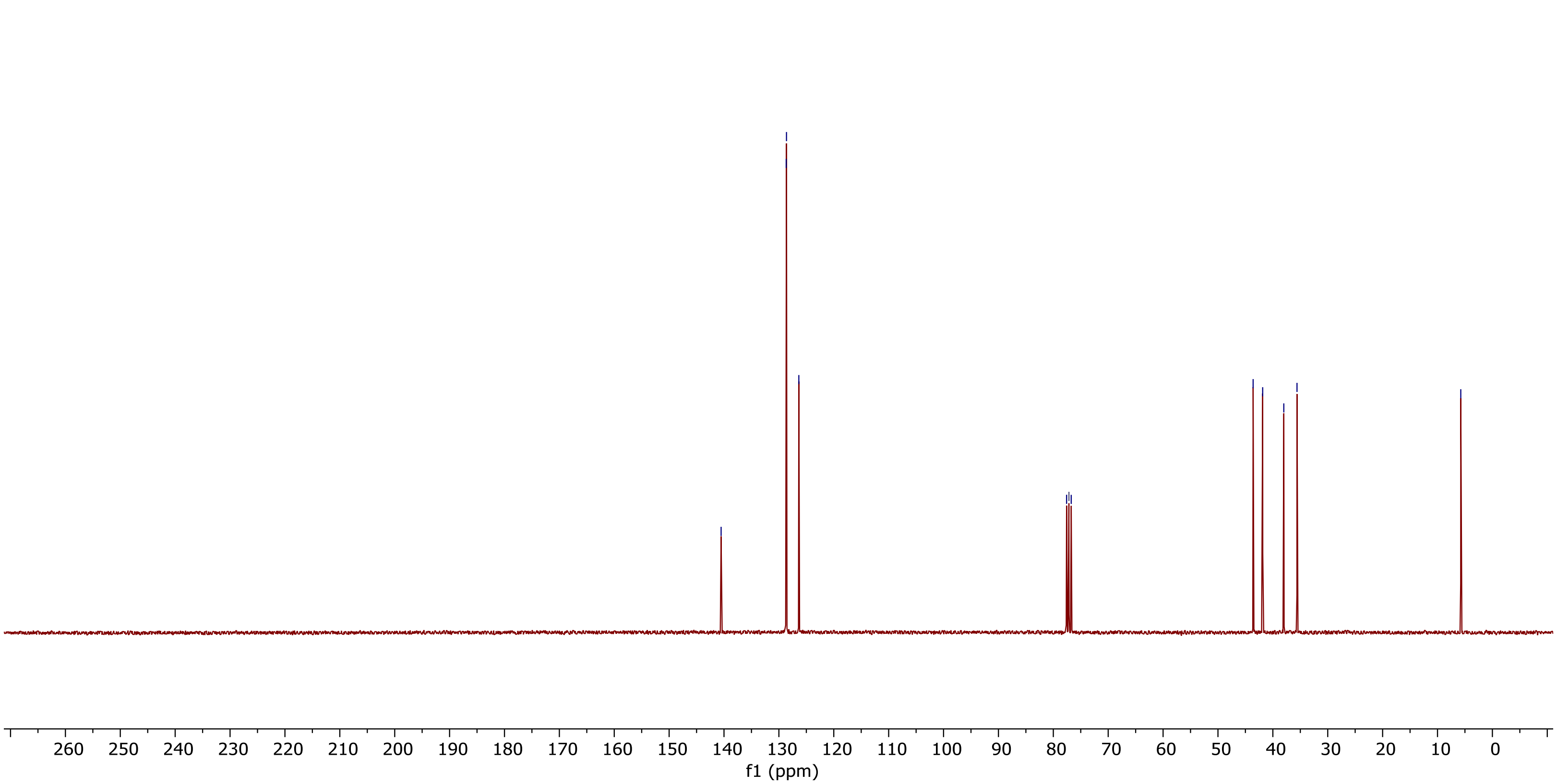

Compound 26 ^{1}H NMR in $CDCl_3$, 298 K, 300 MHz

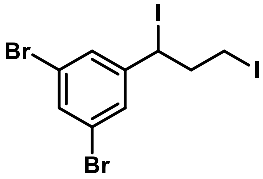

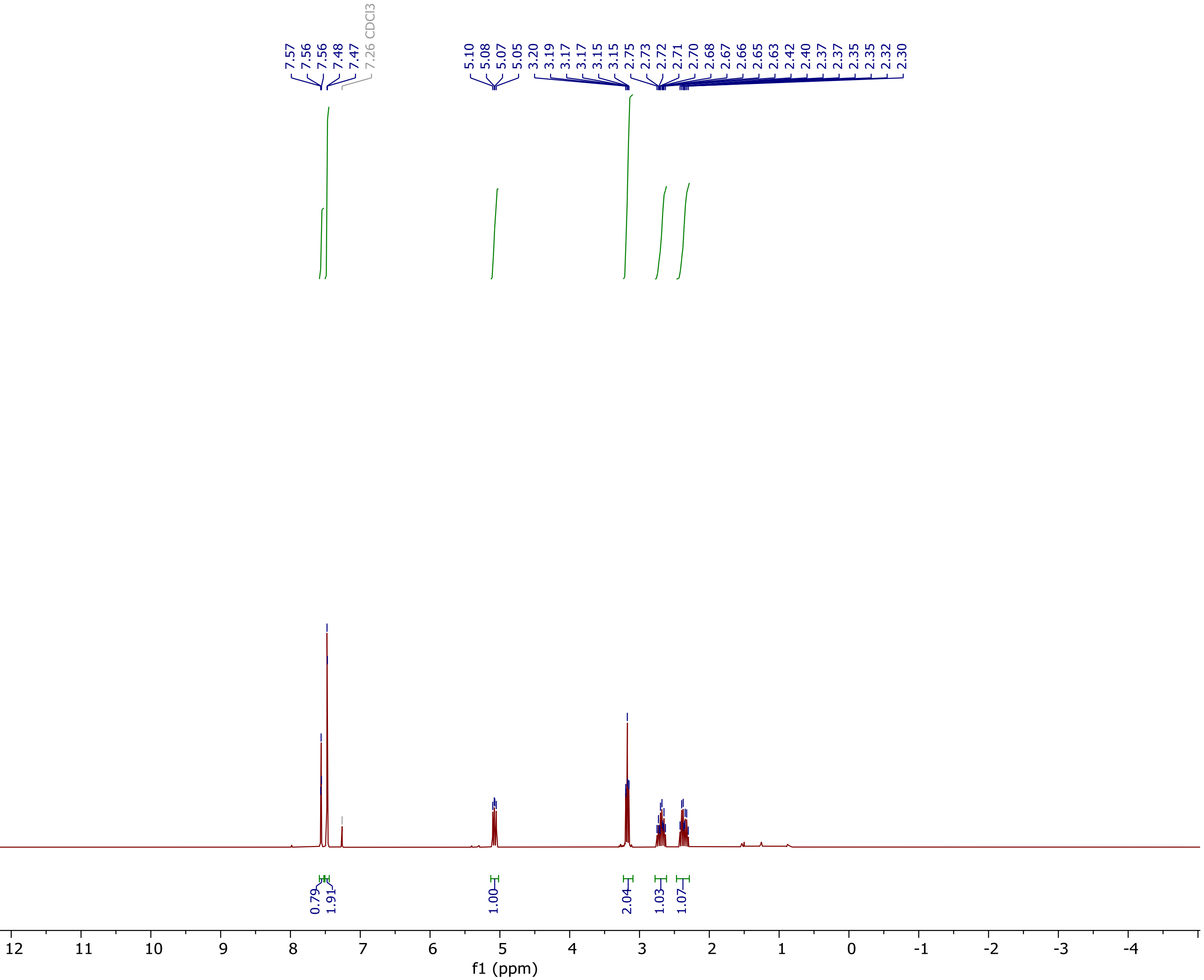

Compound 26 ^{13}C NMR in $CDCl_3$, 298 K, 75 MHz

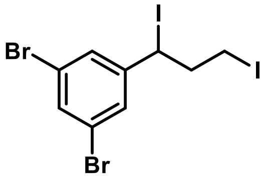

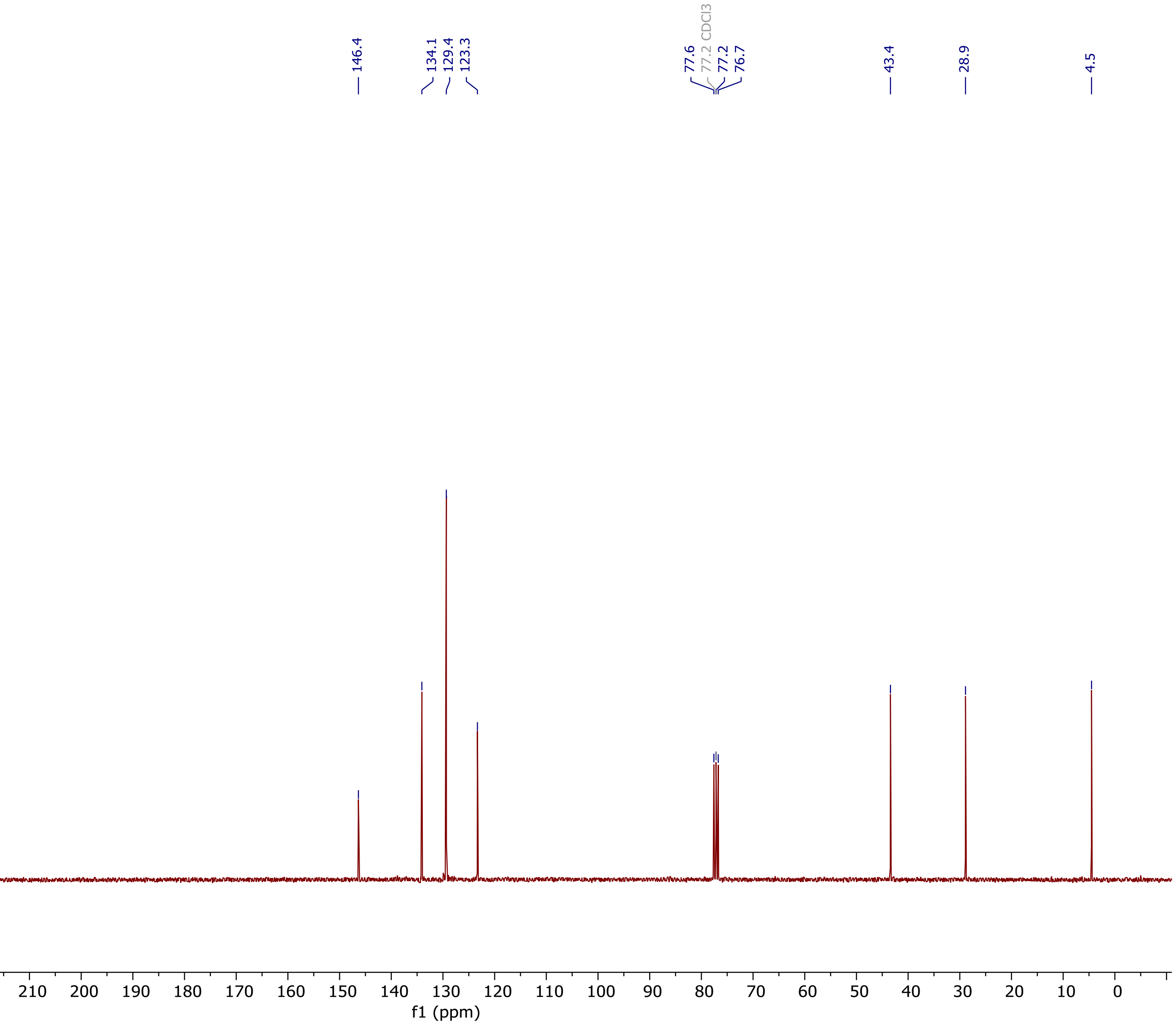

Compound 27 ^{1}H NMR in $CDCl_3$, 298 K, 300 MHz

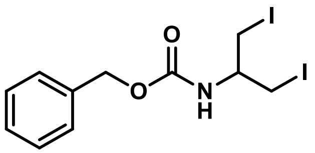

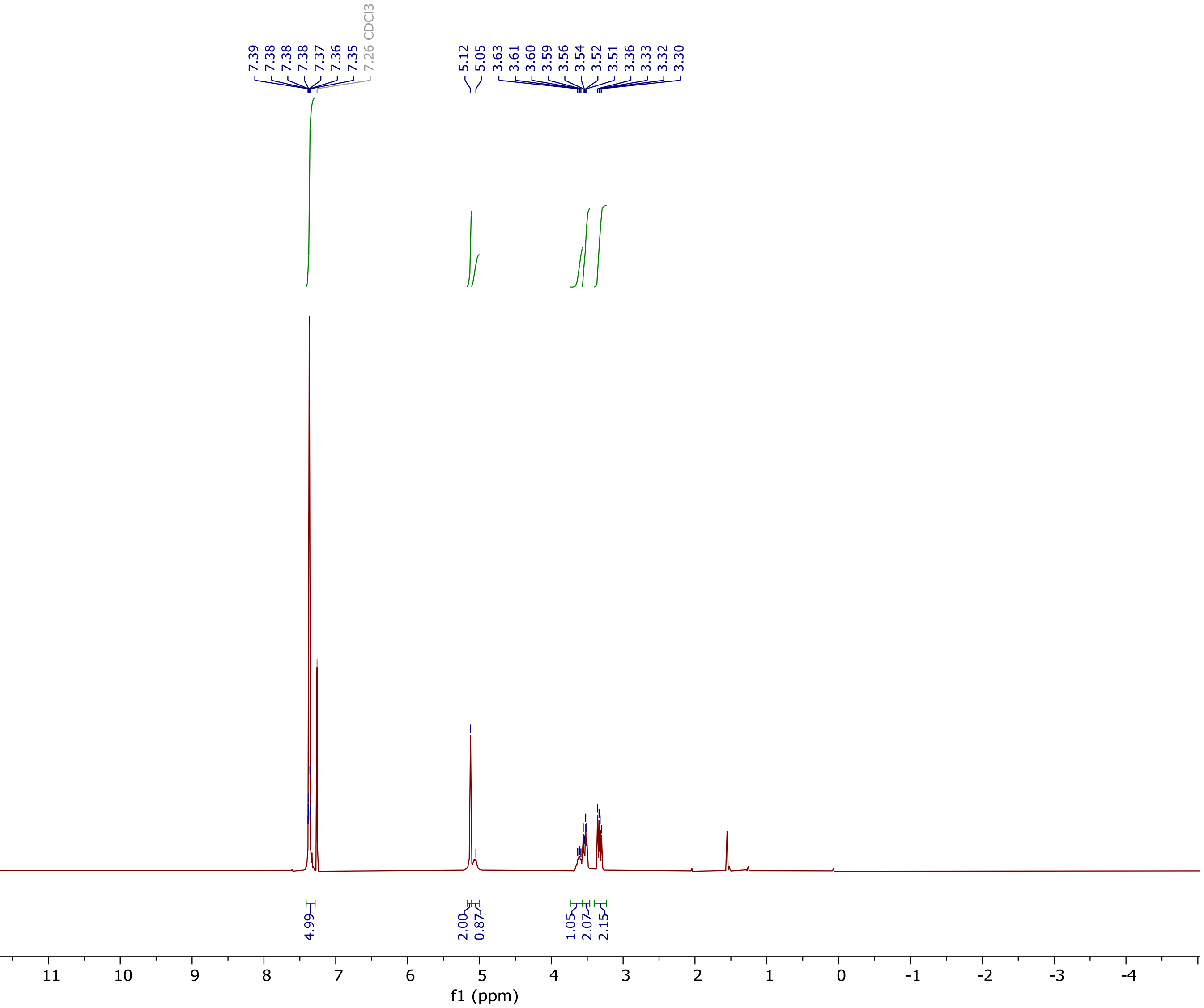

Compound 27 ^{13}C NMR in $CDCl_3$, 298 K, 151 MHz

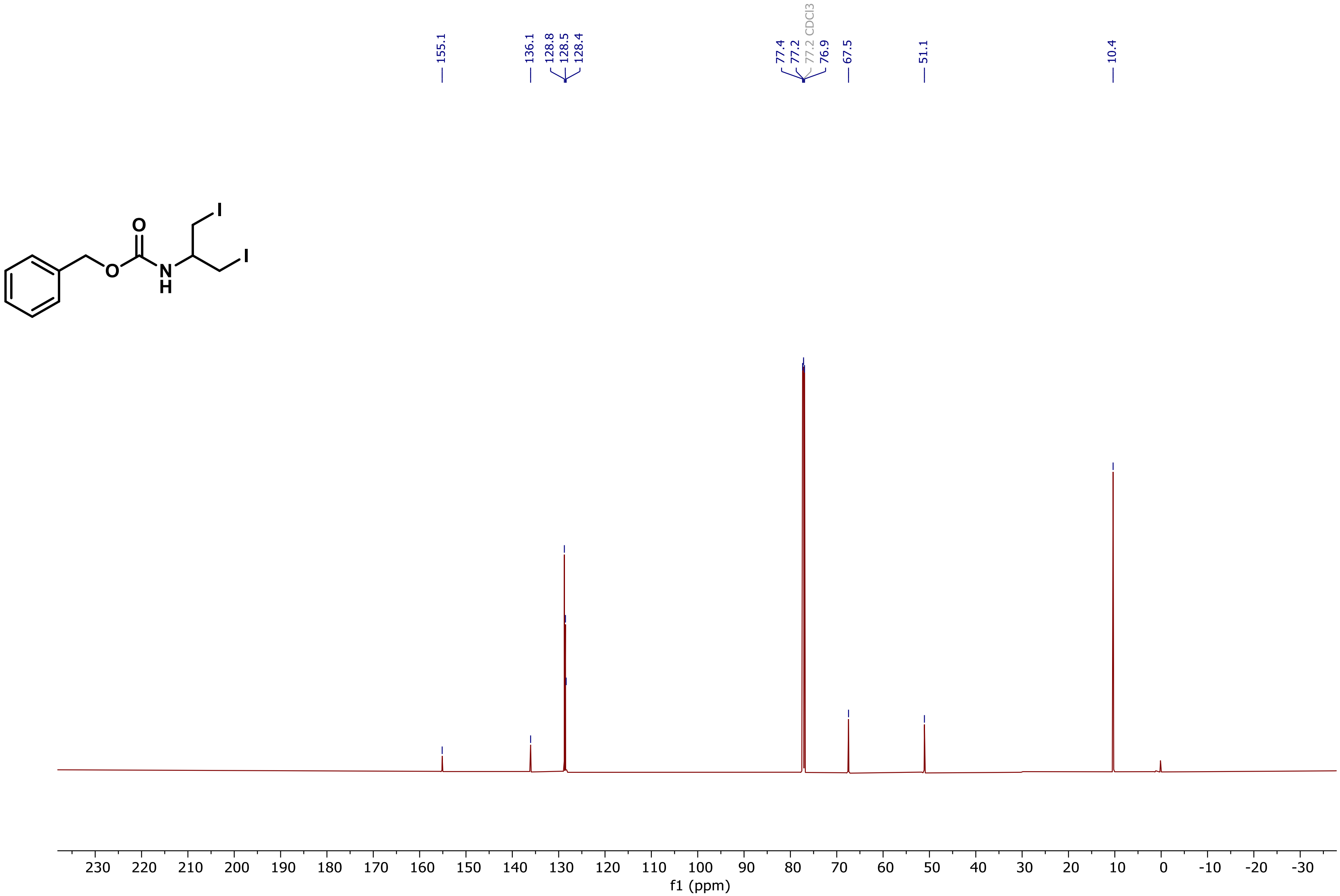

Compound 28 ^{1}H NMR in $CDCl_3$, 298 K, 400 MHz

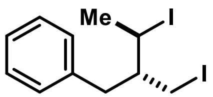

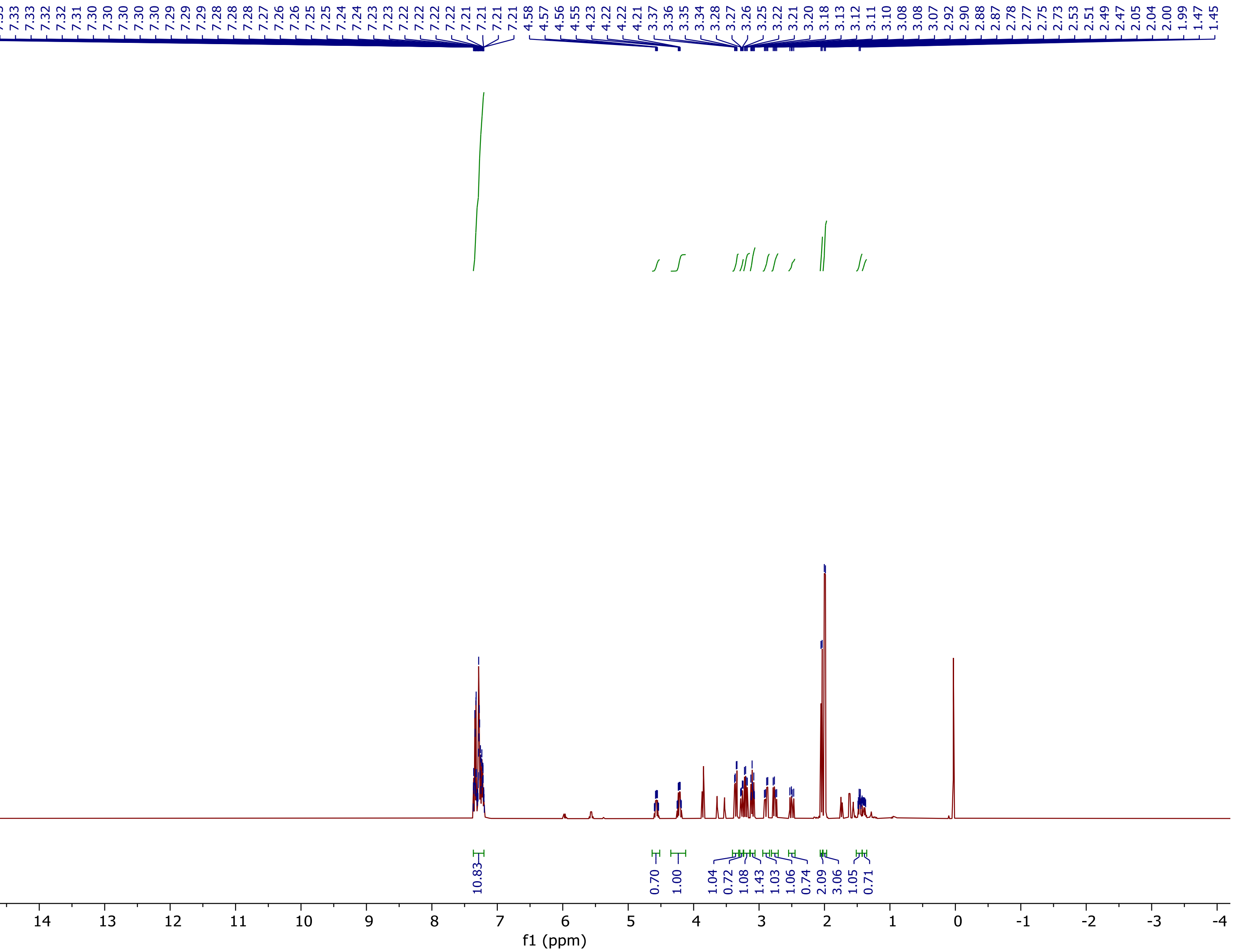

Compound SI-3 ^{13}C NMR in $CDCl_3$, 298 K, 101 MHz

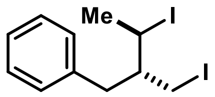

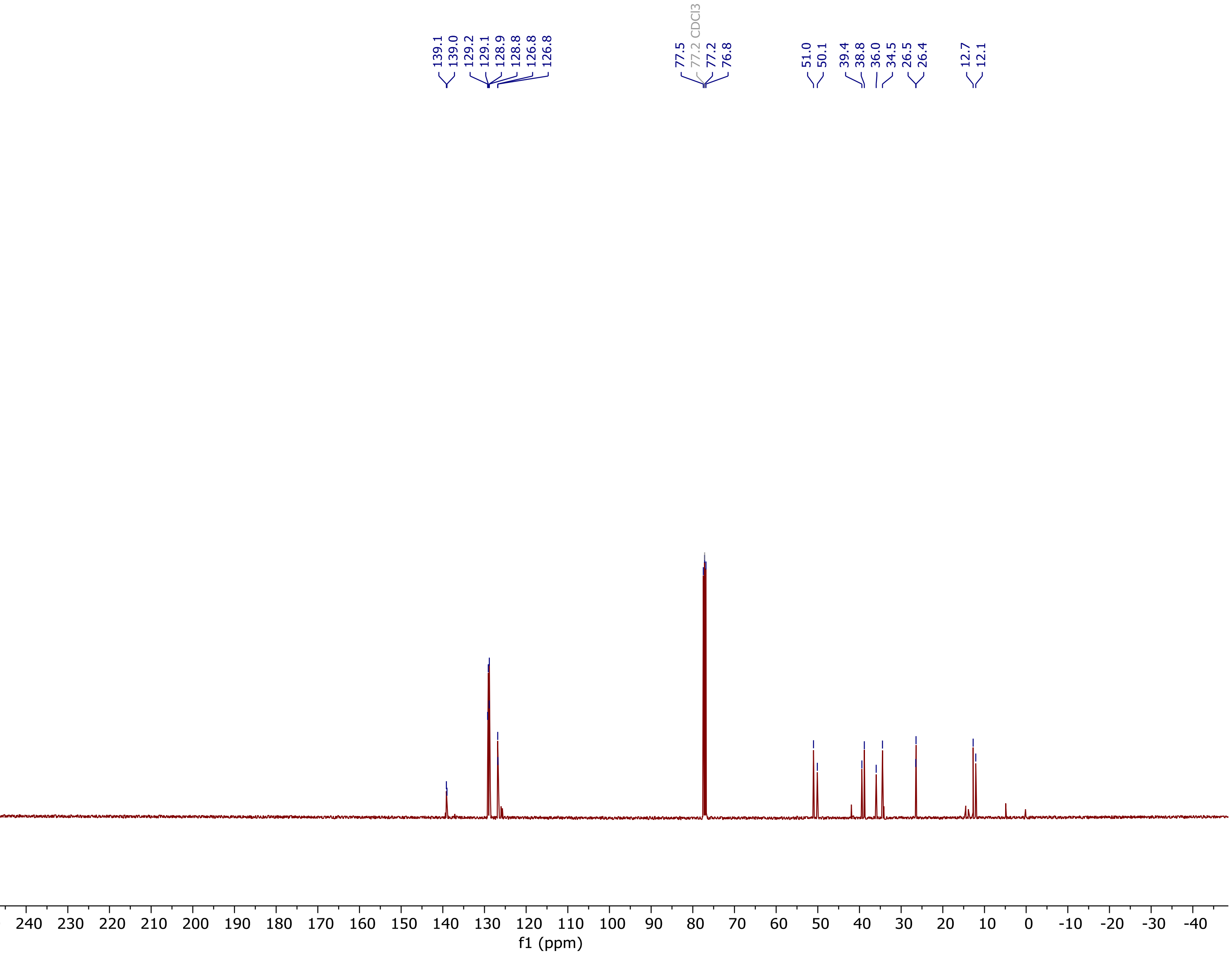

Compound SI-3 ^{1}H NMR in $CDCl_3$, 298 K, 300 MHz

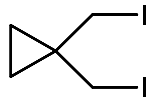

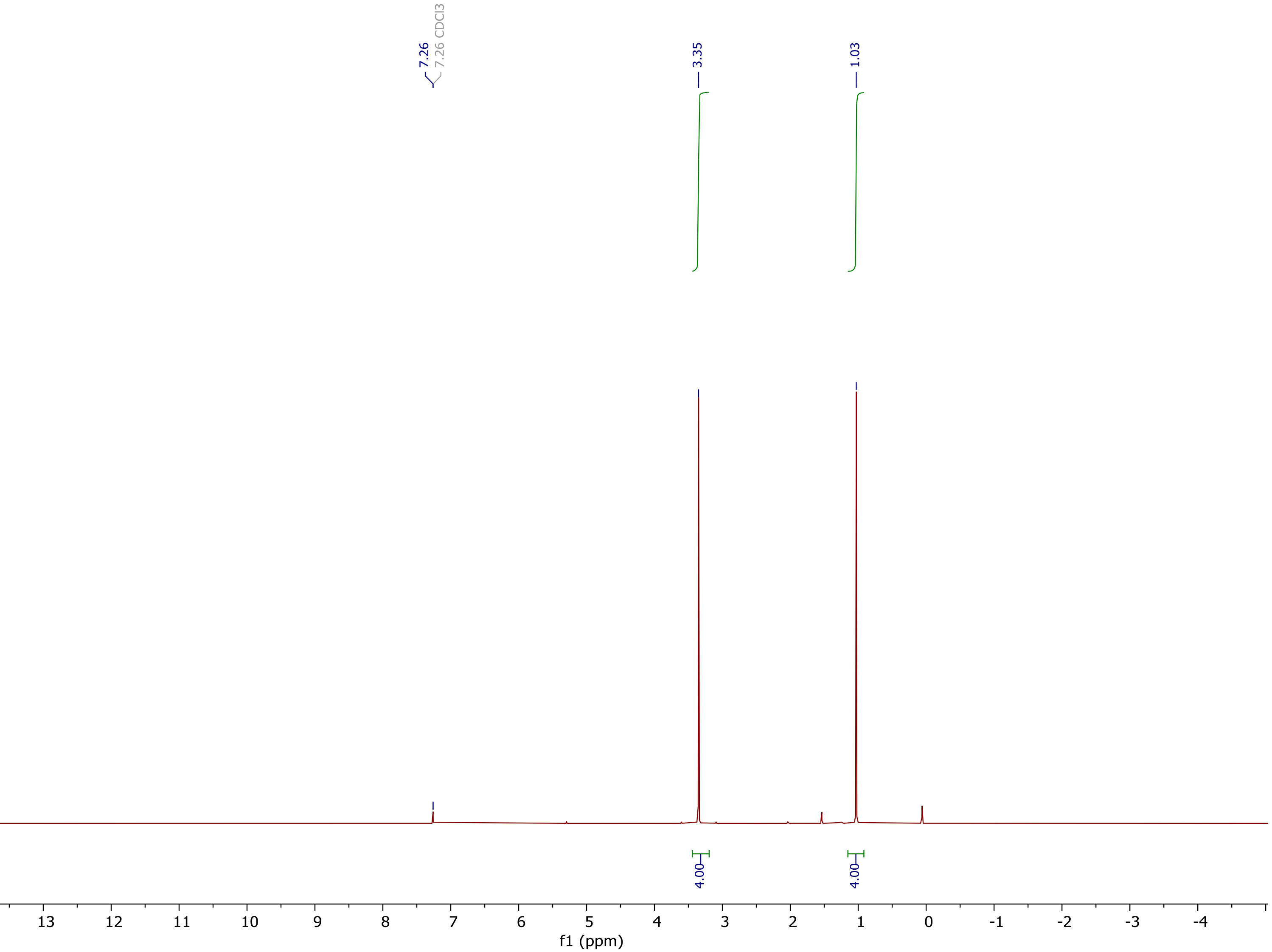

Compound SI-3 ^{13}C NMR in $CDCl_3$, 298 K, 75 MHz

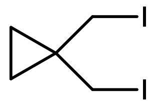

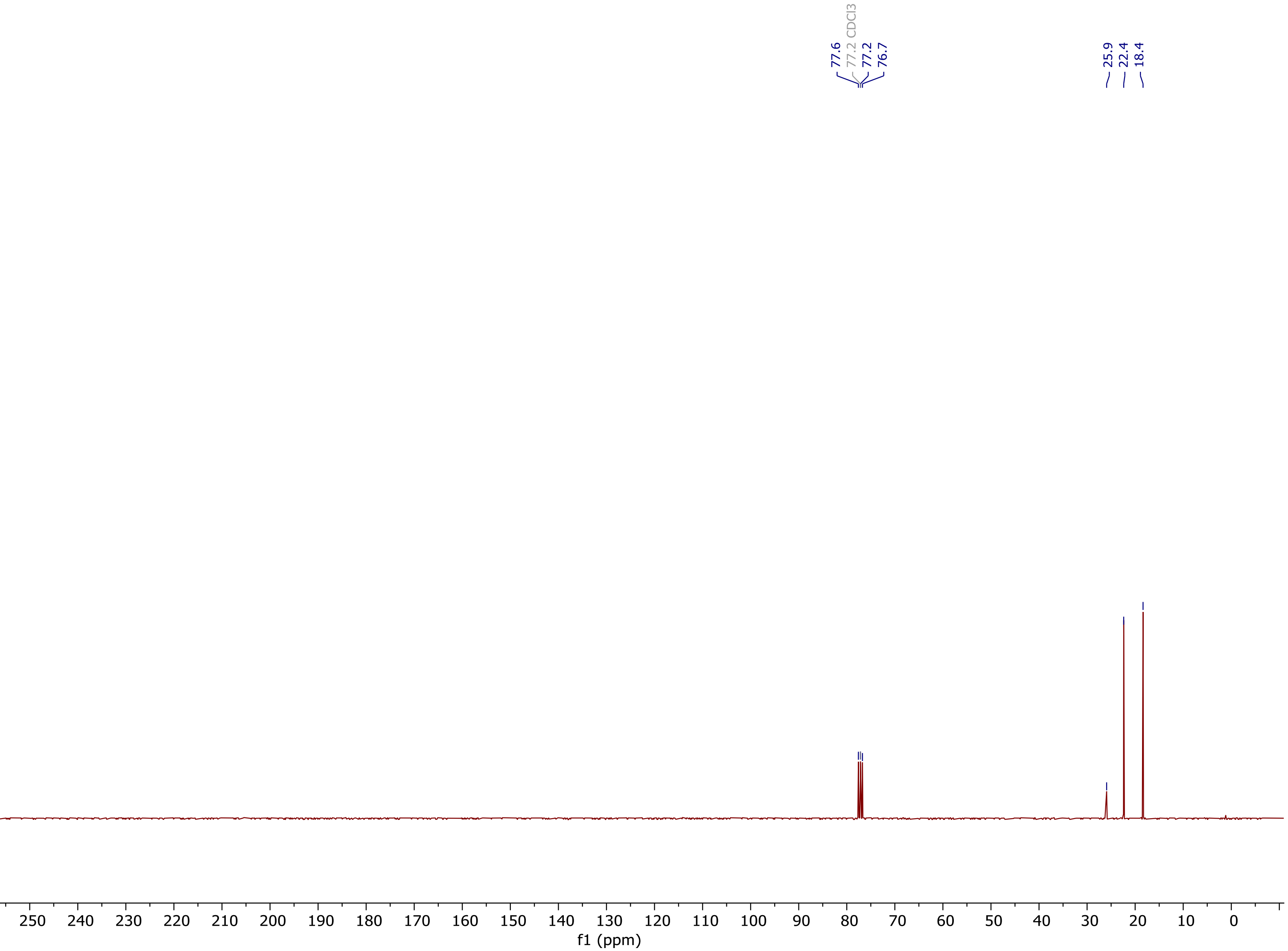

Compound 3 ^{1}H NMR in $CDCl_3$, 298 K, 300 MHz

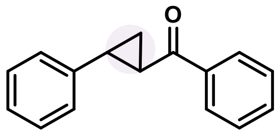

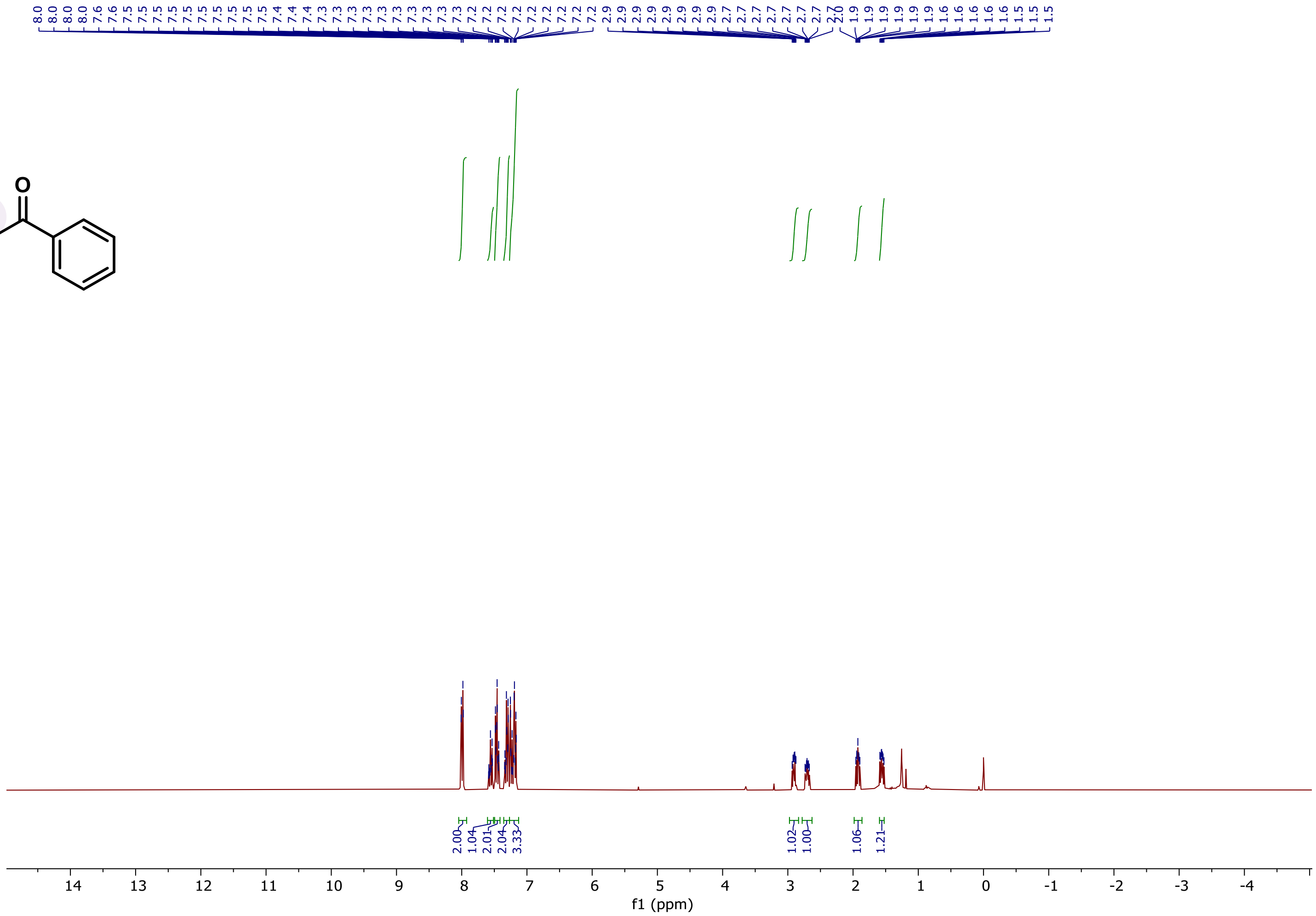

Compound 3 ^{13}C NMR in $CDCl_3$, 298 K, 75 MHz

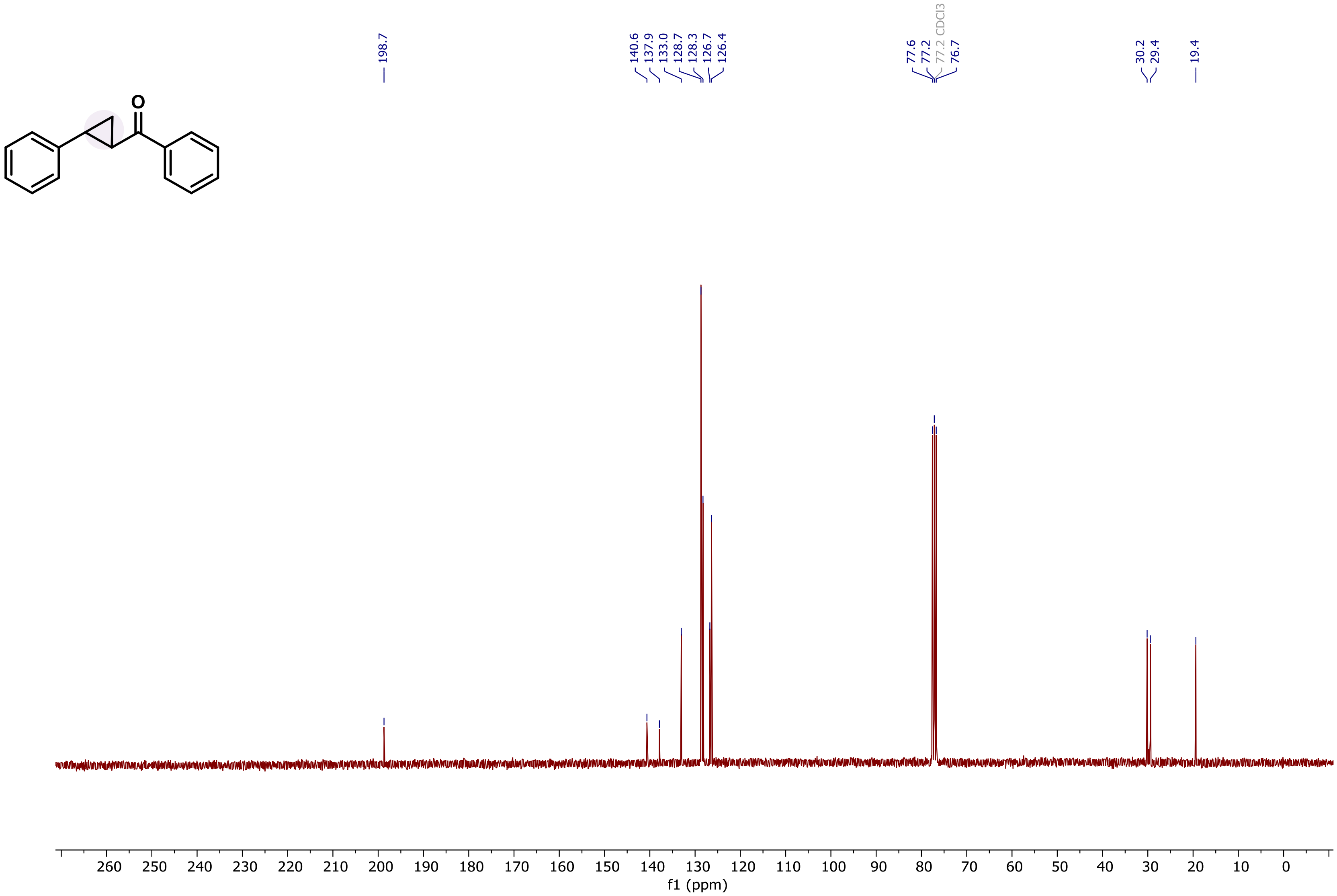

Compound 4 ^{1}H NMR in $CDCl_3$, 298 K, 300 MHz

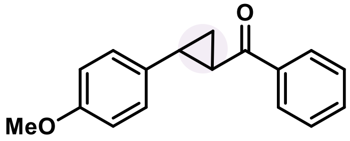

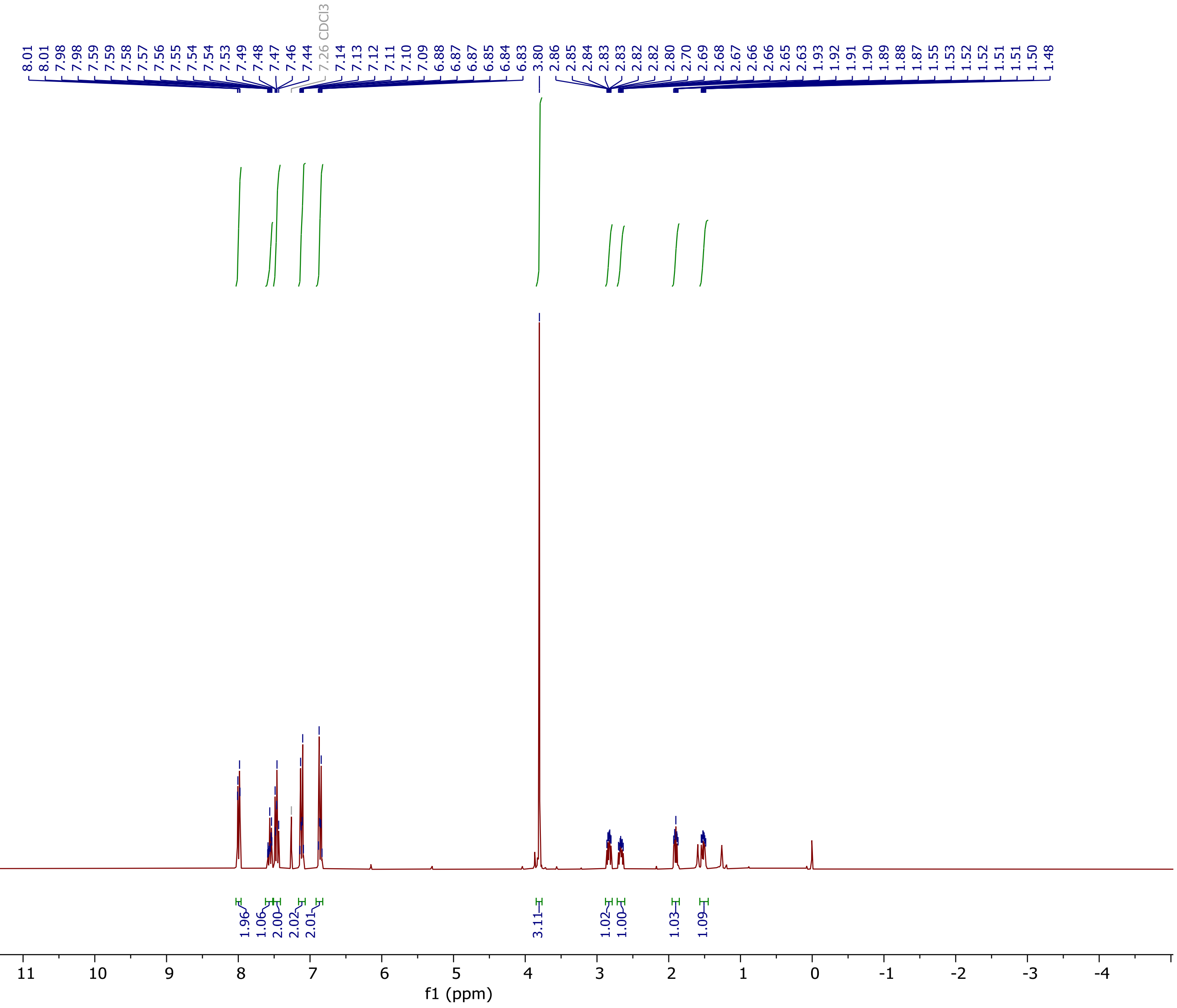

Compound 4 ^{13}C NMR in $CDCl_3$, 298 K, 75 MHz

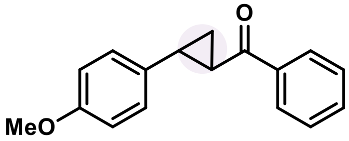

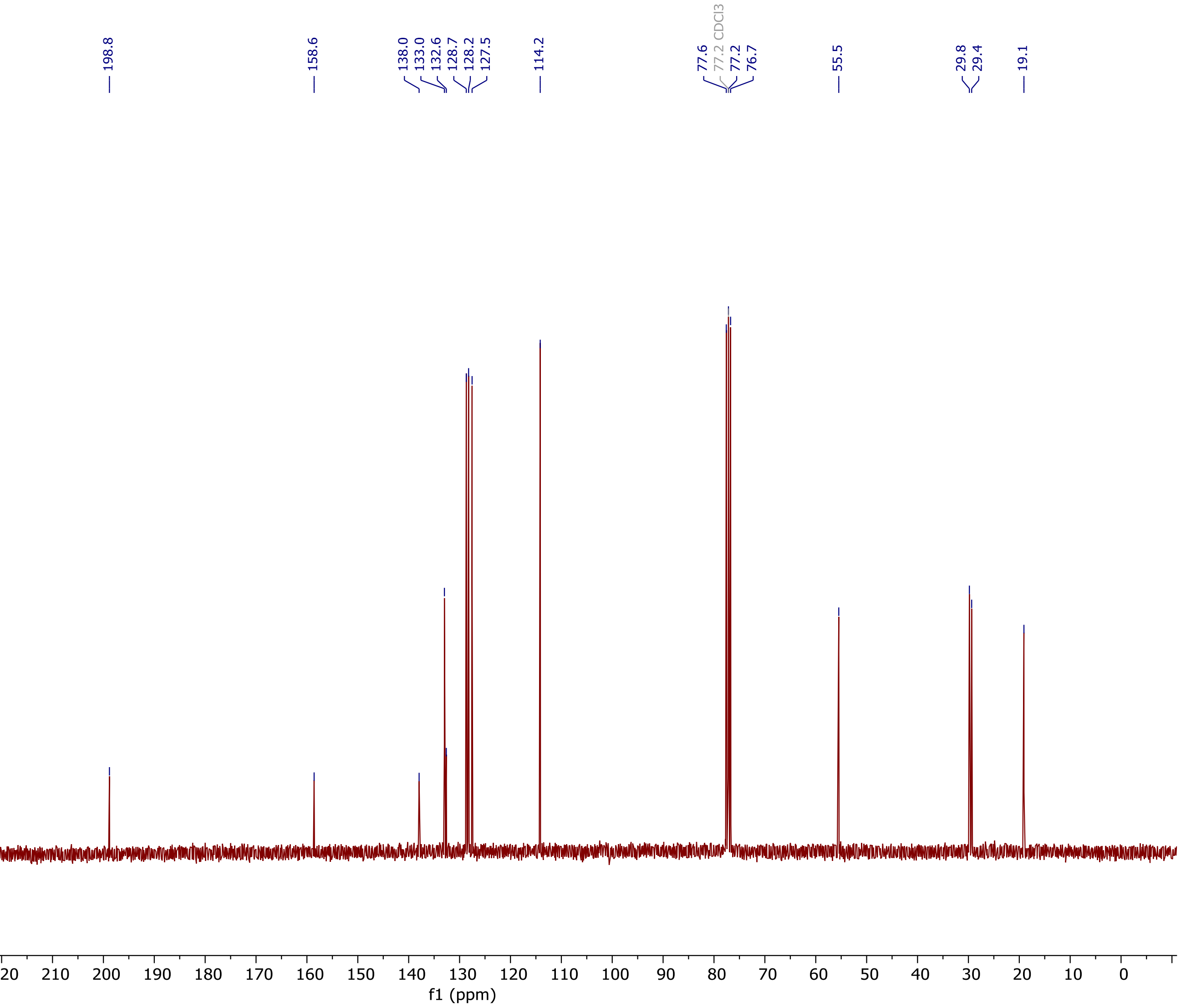

Compound 5 ^{1}H NMR in $CDCl_3$, 298 K, 300 MHz

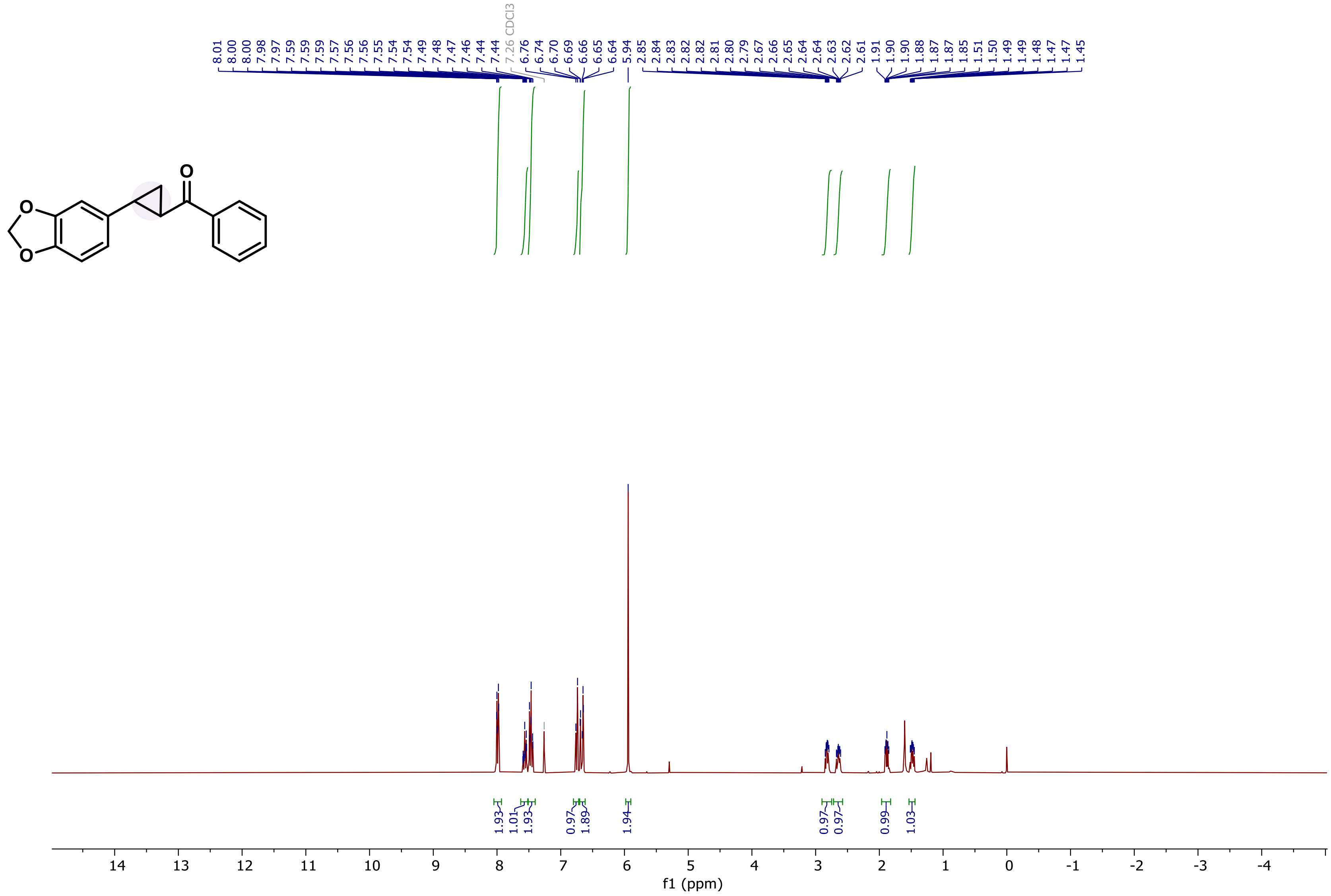

Compound 5 ^{13}C NMR in $CDCl_3$, 298 K, 75 MHz

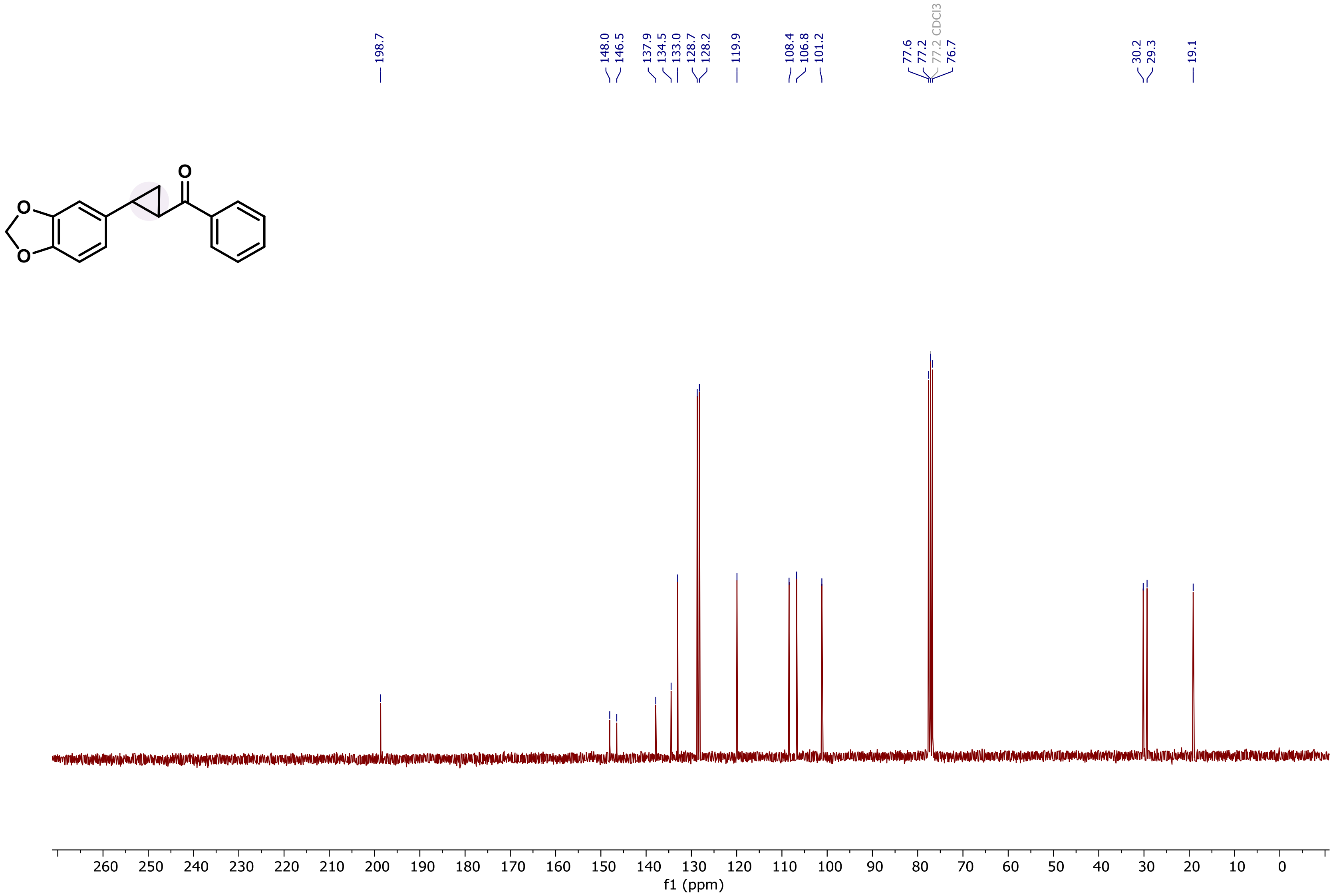

Compound 6 ^{1}H NMR in $CDCl_3$, 298 K, 300 MHz

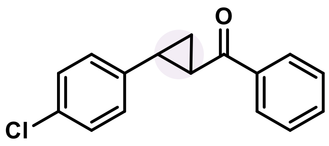

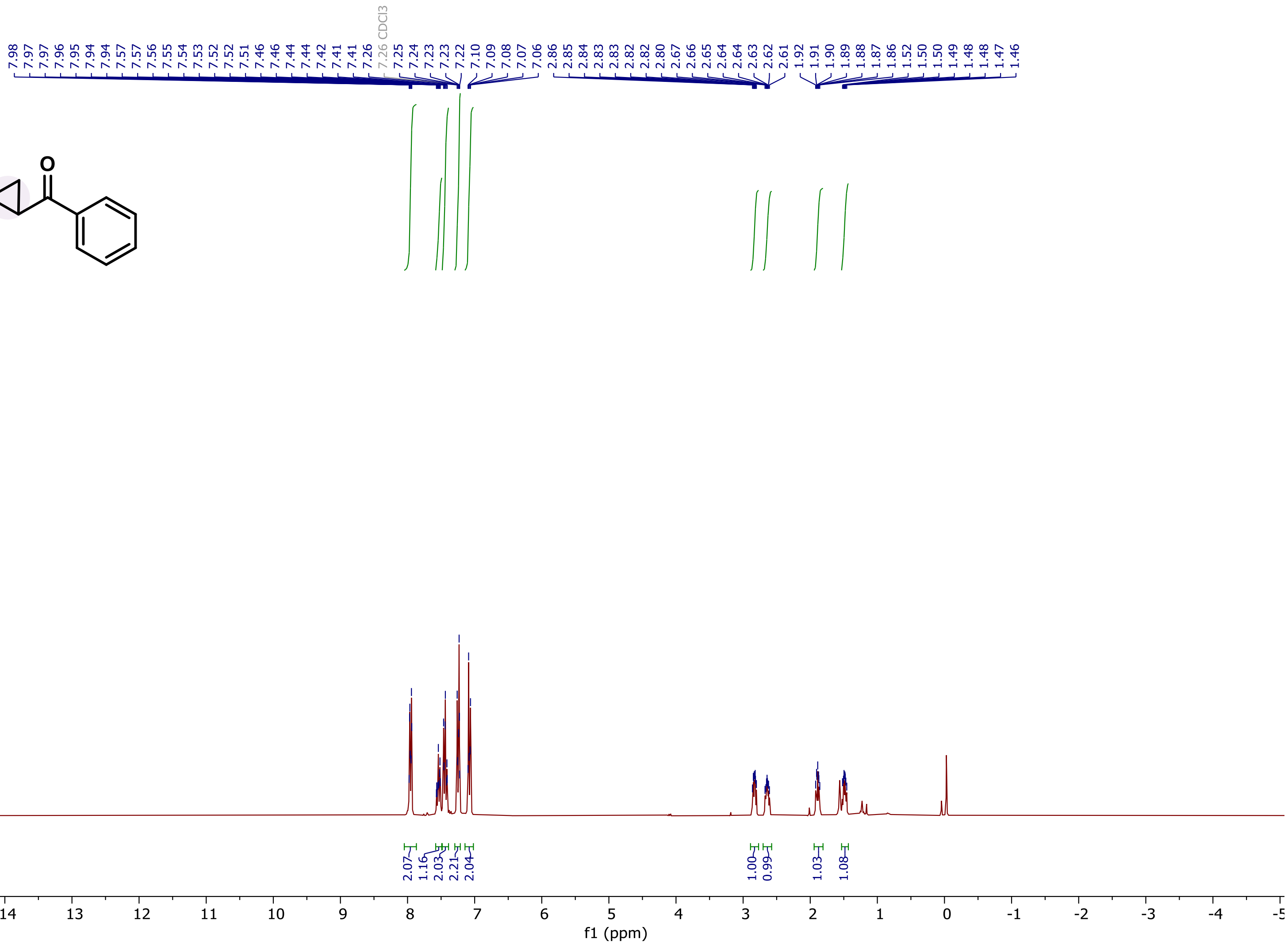

Compound 6 ^{13}C NMR in $CDCl_3$, 298 K, 75 MHz

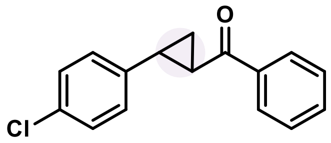

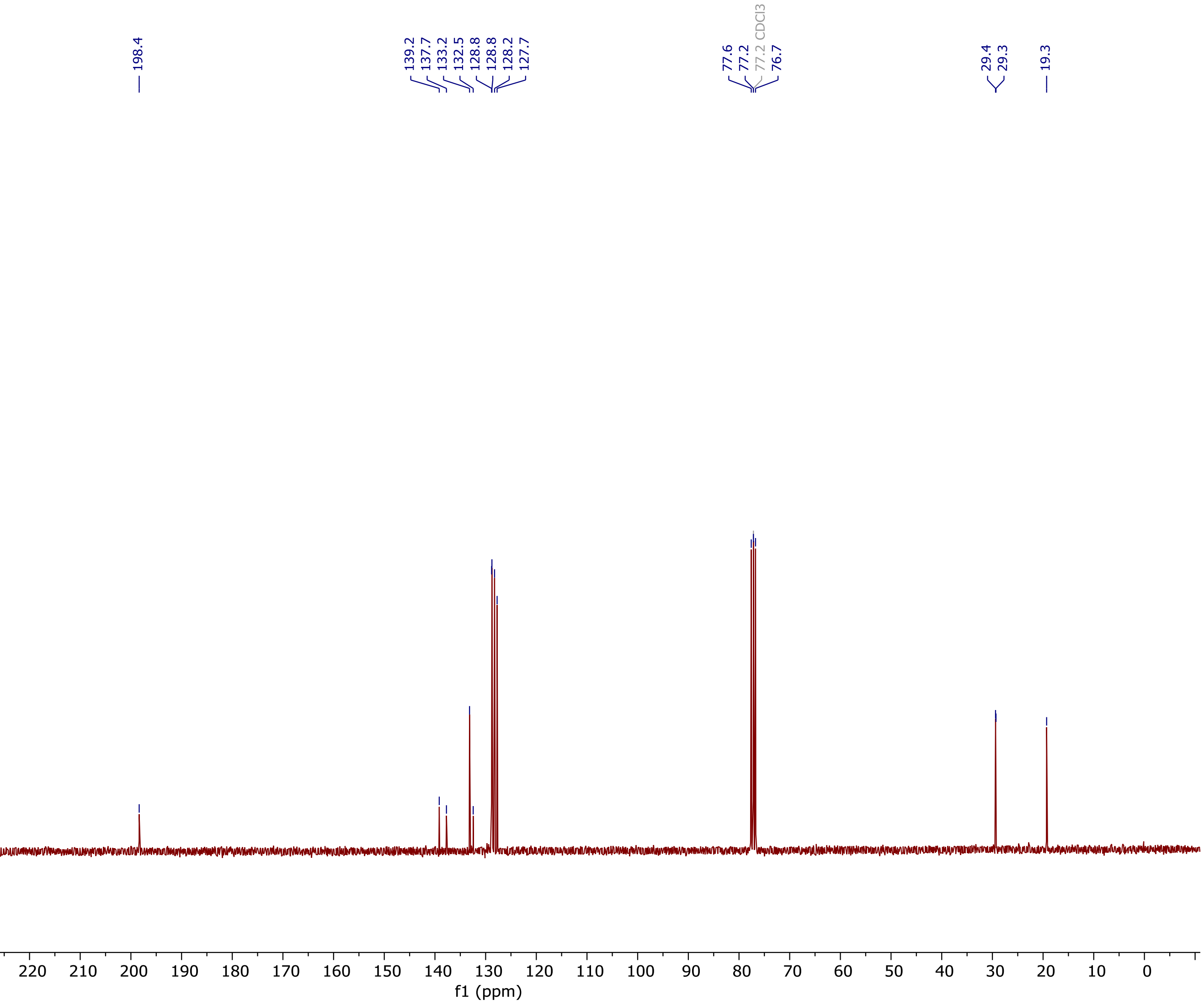

Compound 7 ^{1}H NMR in $CDCl_3$, 298 K, 300 MHz

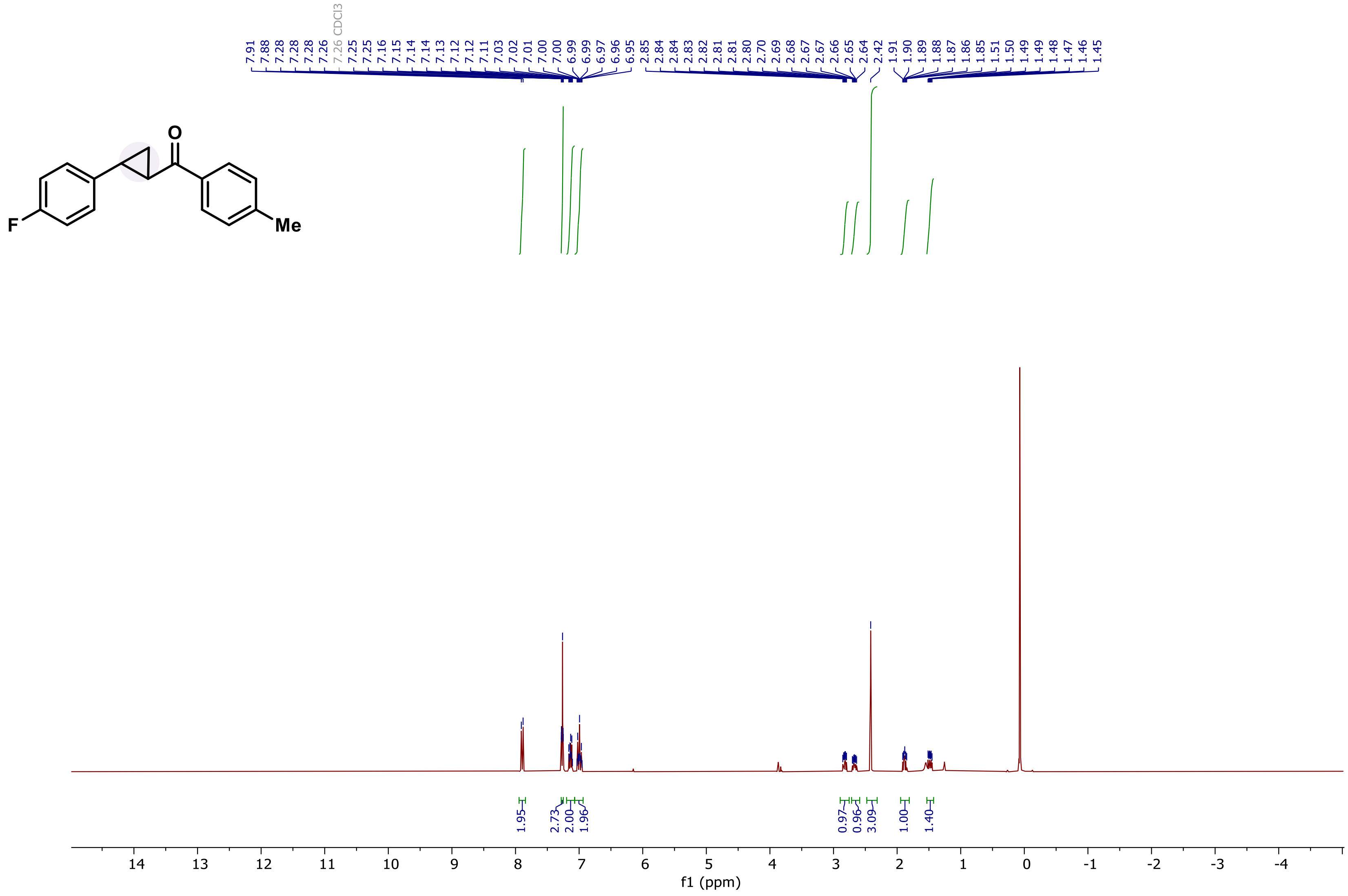

Compound 7 ^{13}C NMR in $CDCl_3$, 298 K, 75 MHz

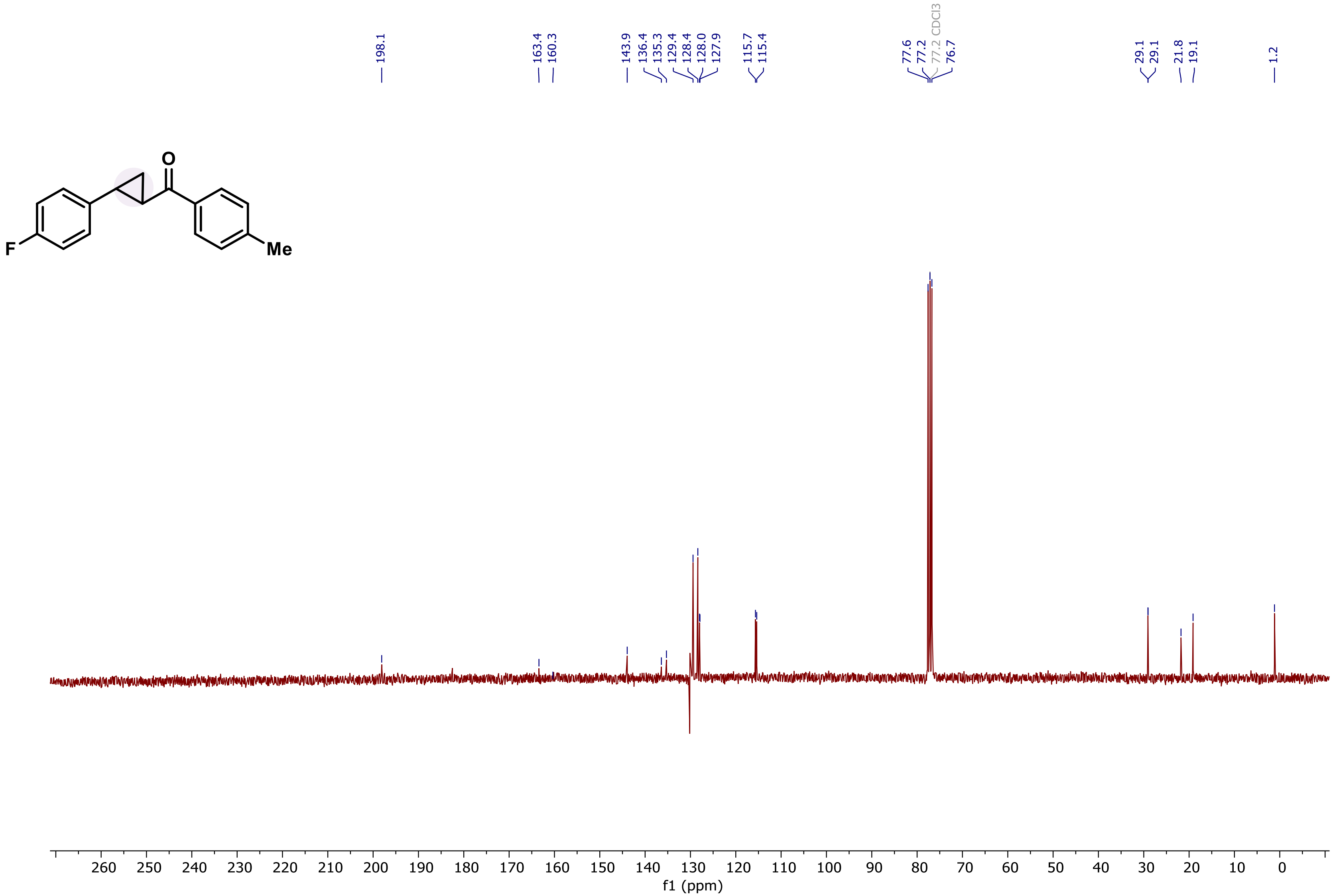

Compound 7 ^{19}F NMR in $CDCl_3$, 298 K, 282 MHz

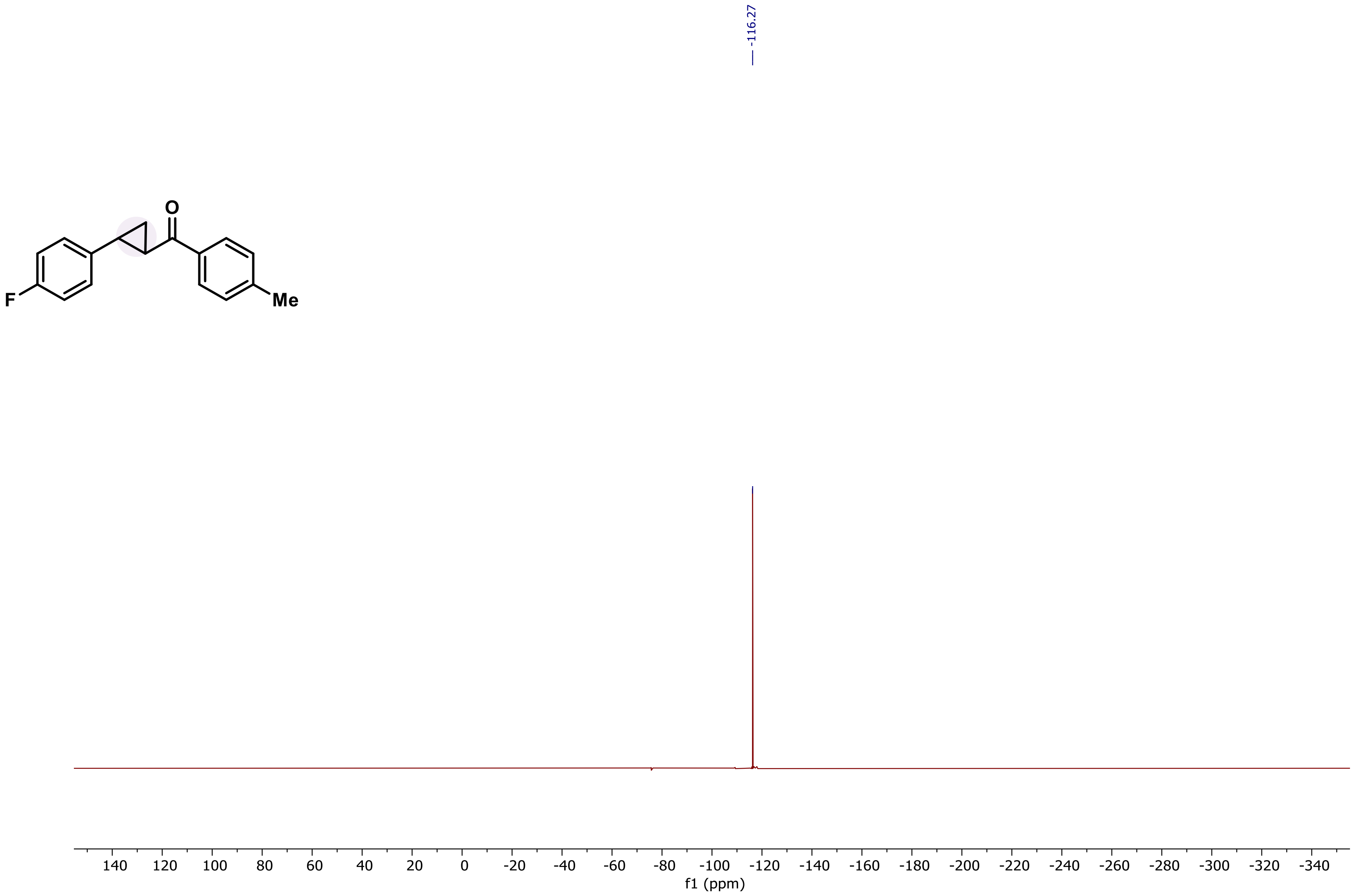

Compound 8 ^{1}H NMR in $CDCl_3$, 298 K, 300 MHz

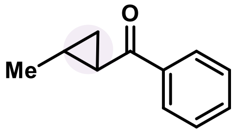

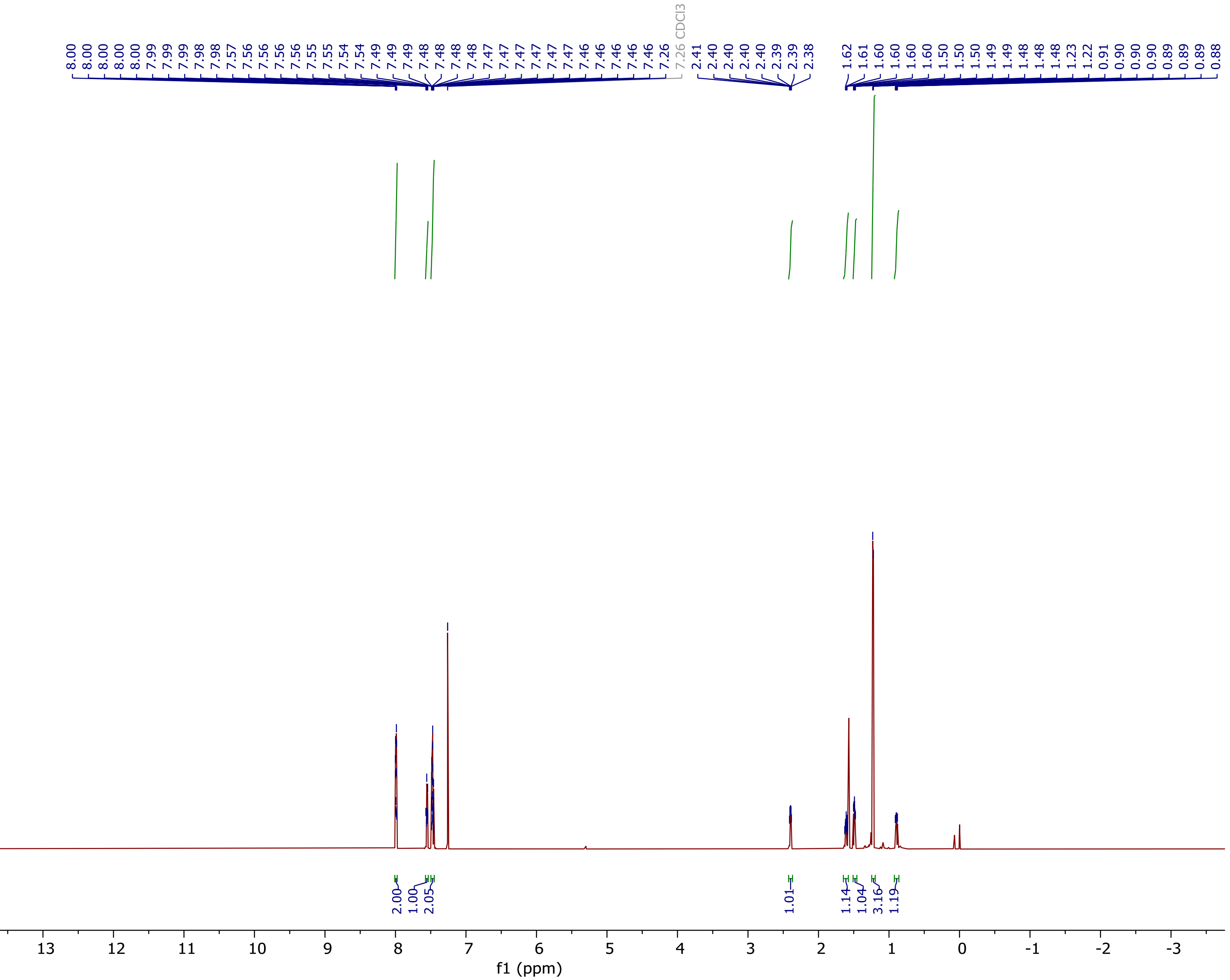

Compound 8 ^{13}C NMR in $CDCl_3$, 298 K, 75 MHz

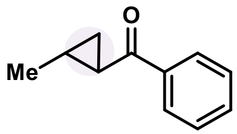

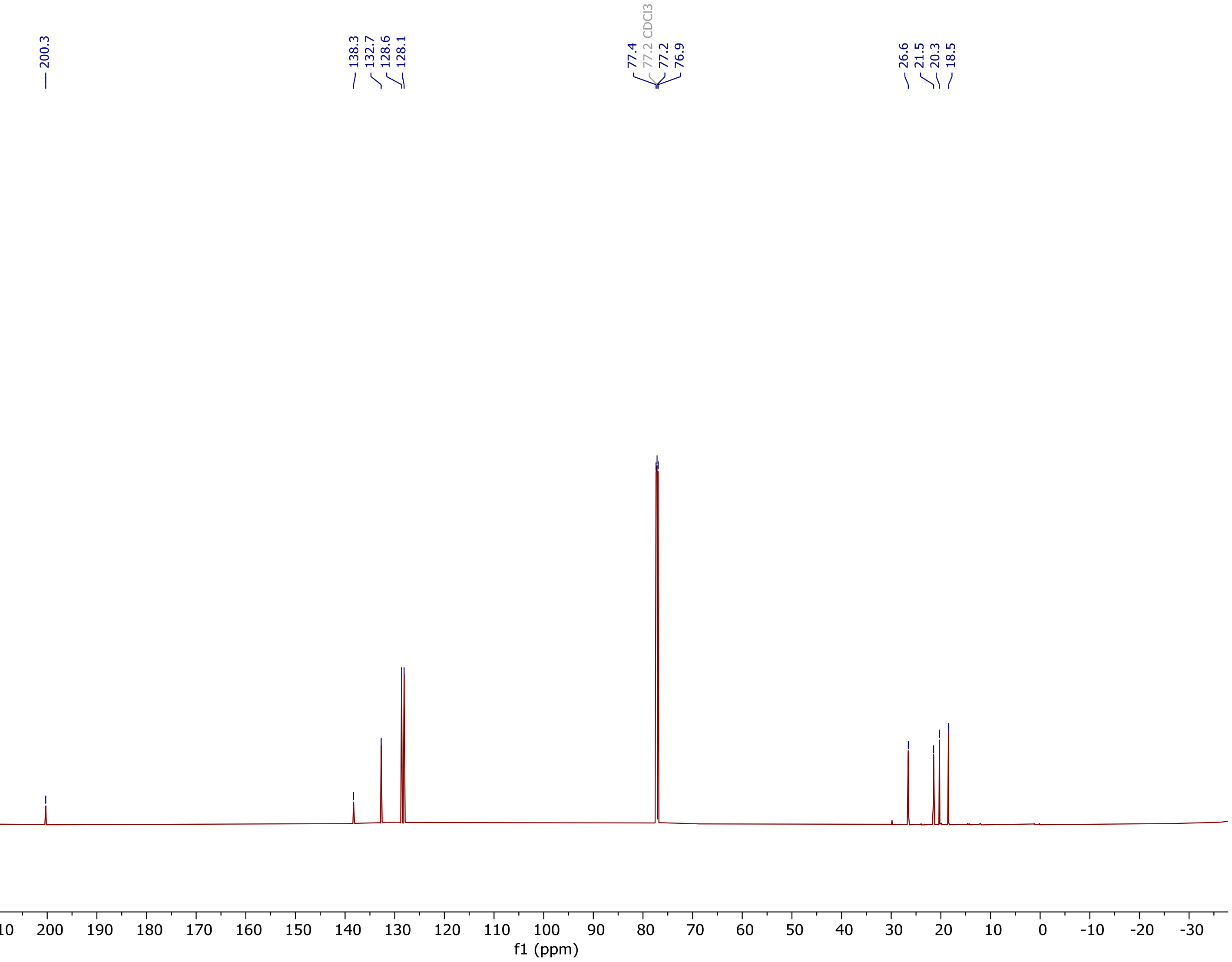

Compound 9 ^{1}H NMR in $CDCl_3$, 298 K, 300 MHz

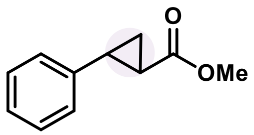

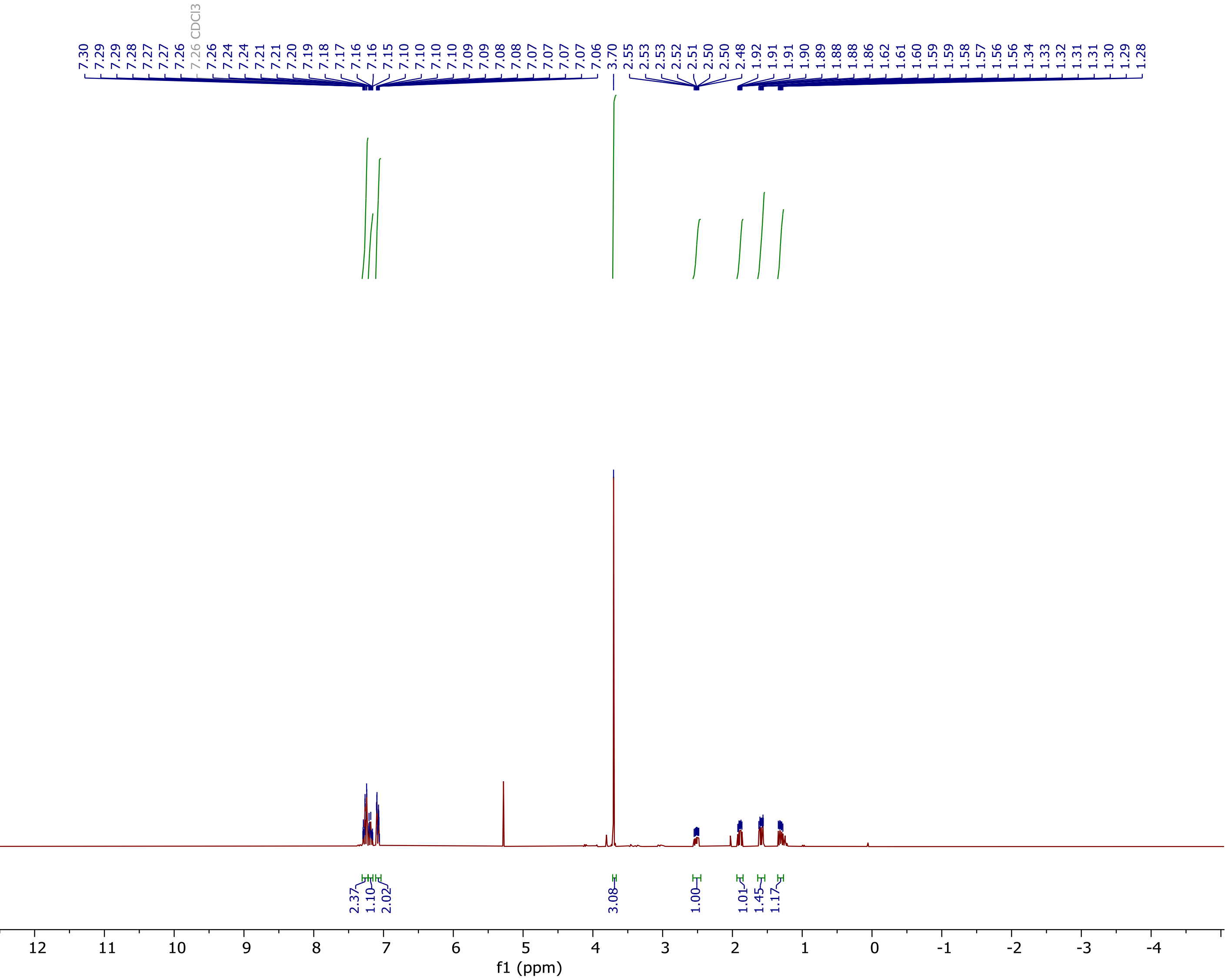

Compound 9 ^{13}C NMR in $CDCl_3$, 298 K, 75 MHz

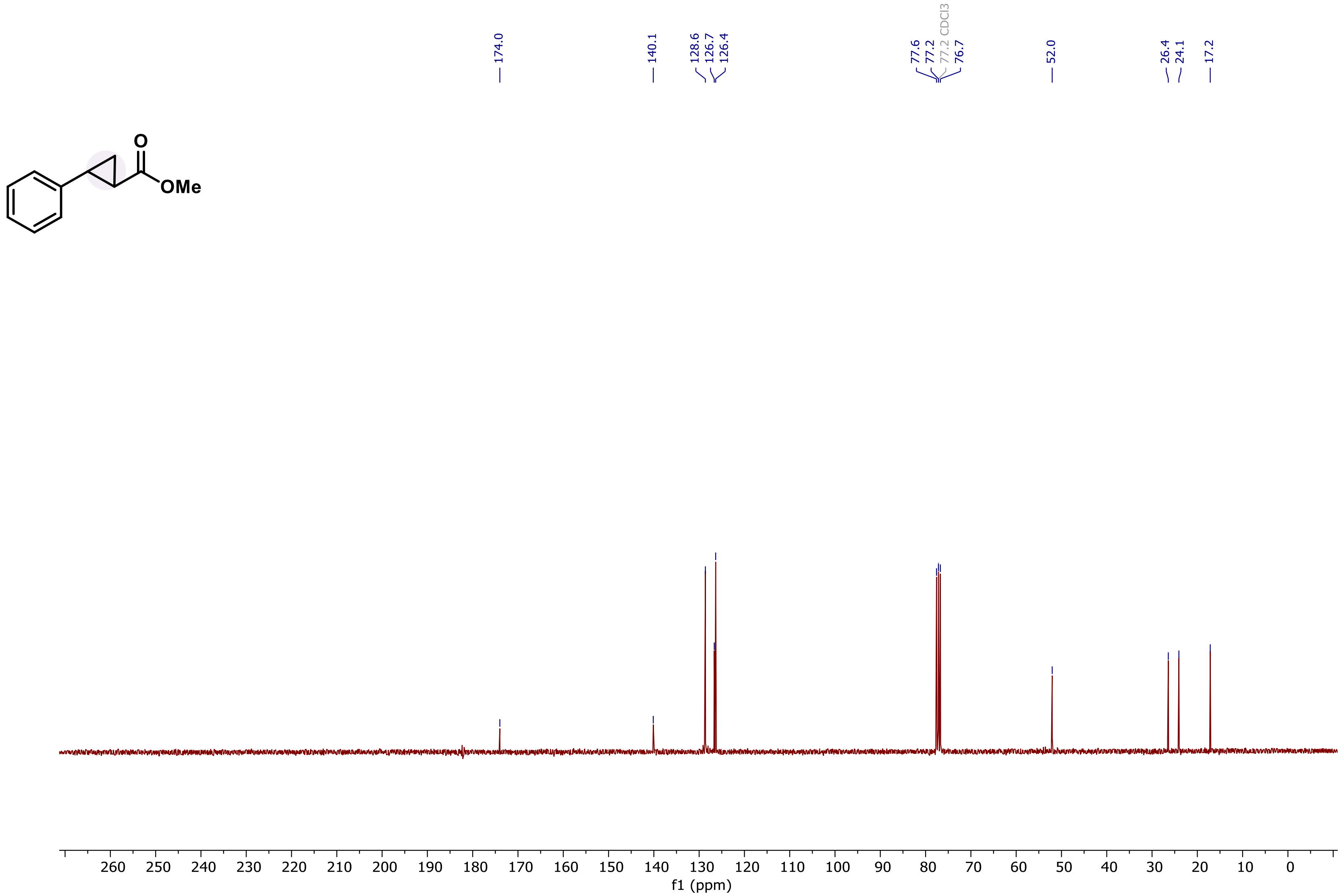

Compound 10 ^{1}H NMR in $CDCl_3$, 298 K, 300 MHz

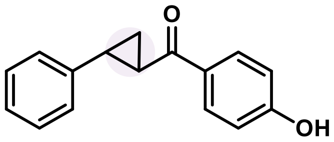

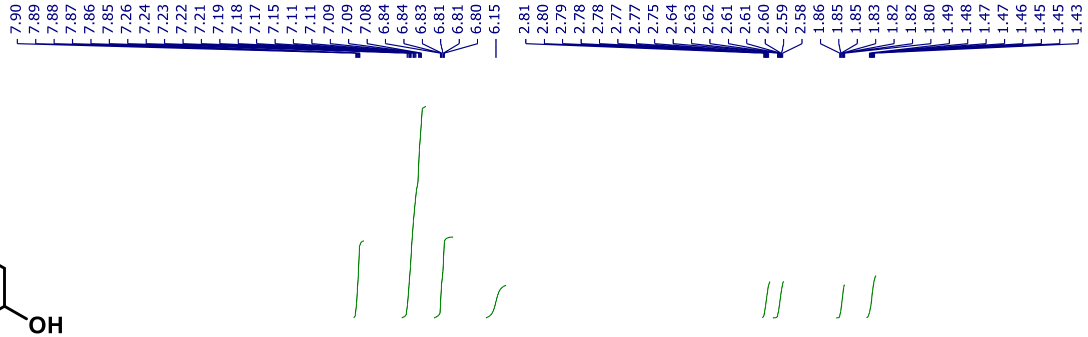

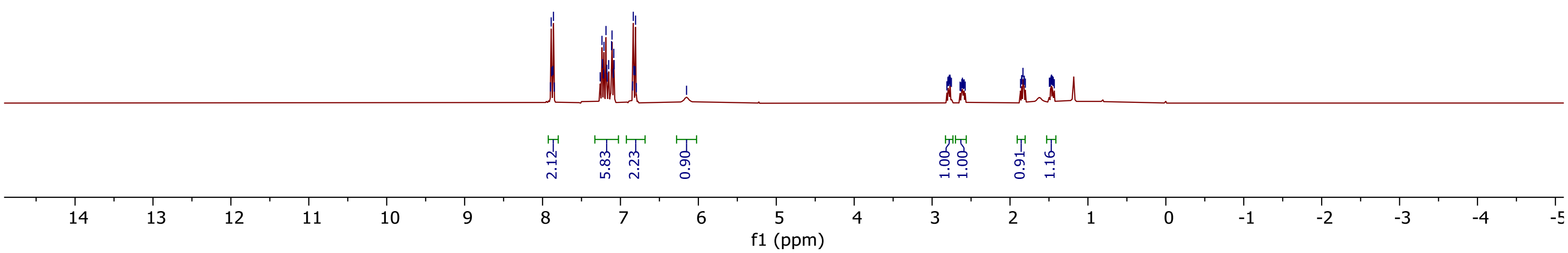

Compound 10 ^{13}C NMR in $CDCl_3$, 298 K, 75 MHz

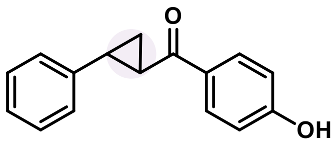

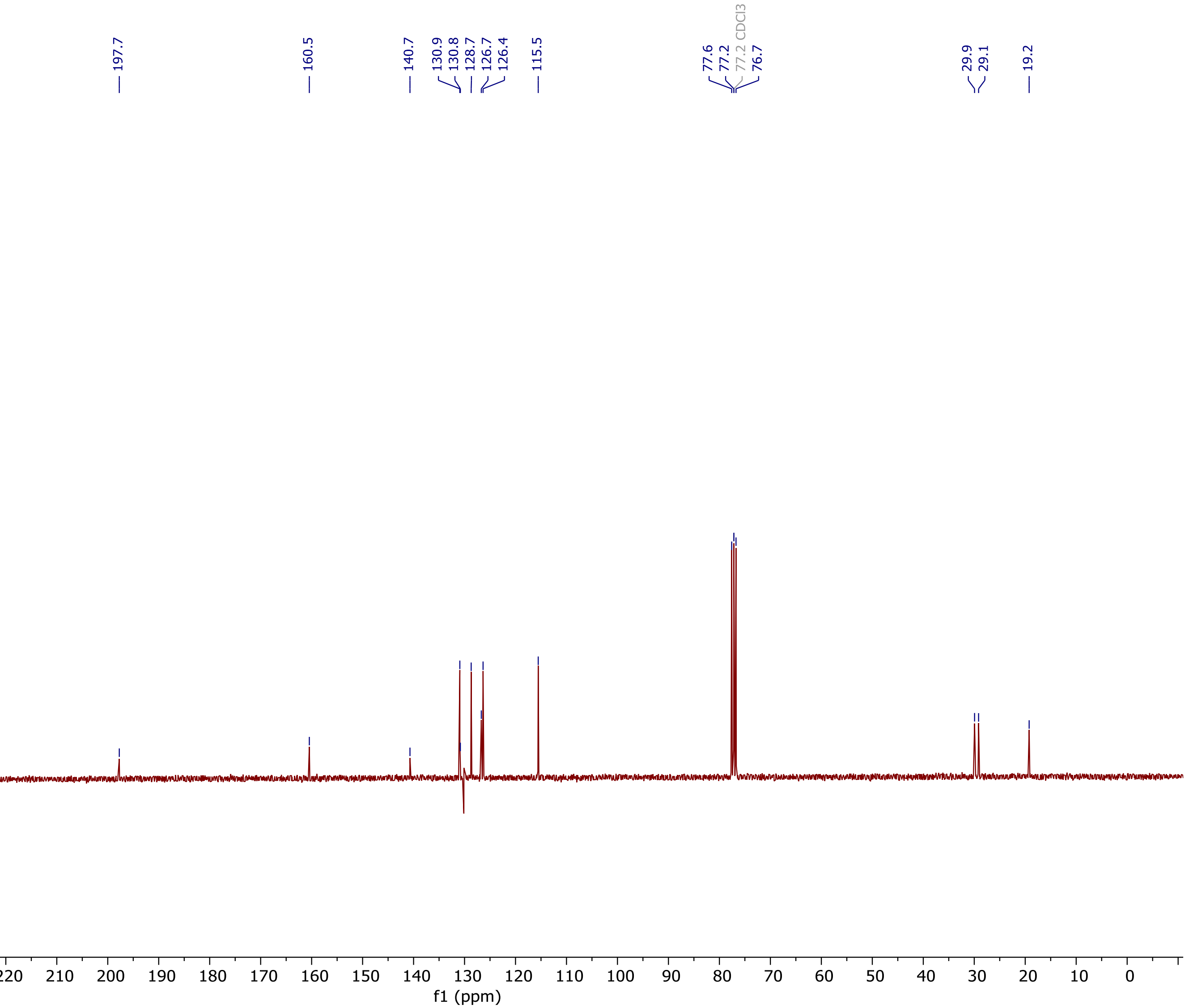

Compound 11 ^{1}H NMR in $CDCl_3$, 298 K, 300 MHz

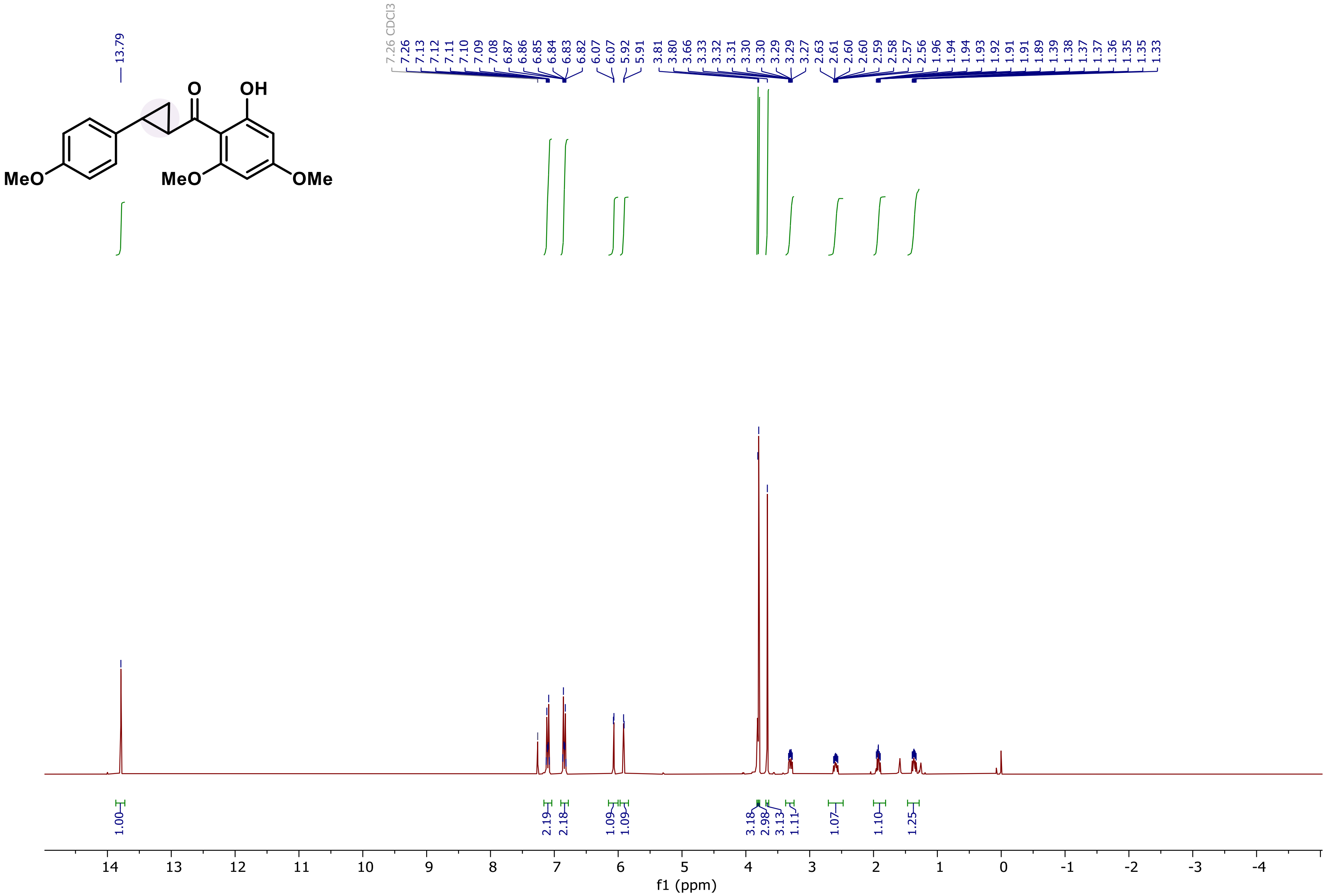

Compound 11 ^{13}C NMR in $CDCl_3$, 298 K, 75 MHz

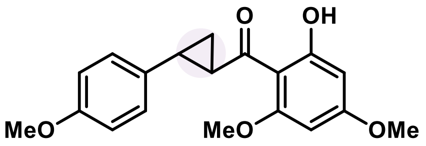

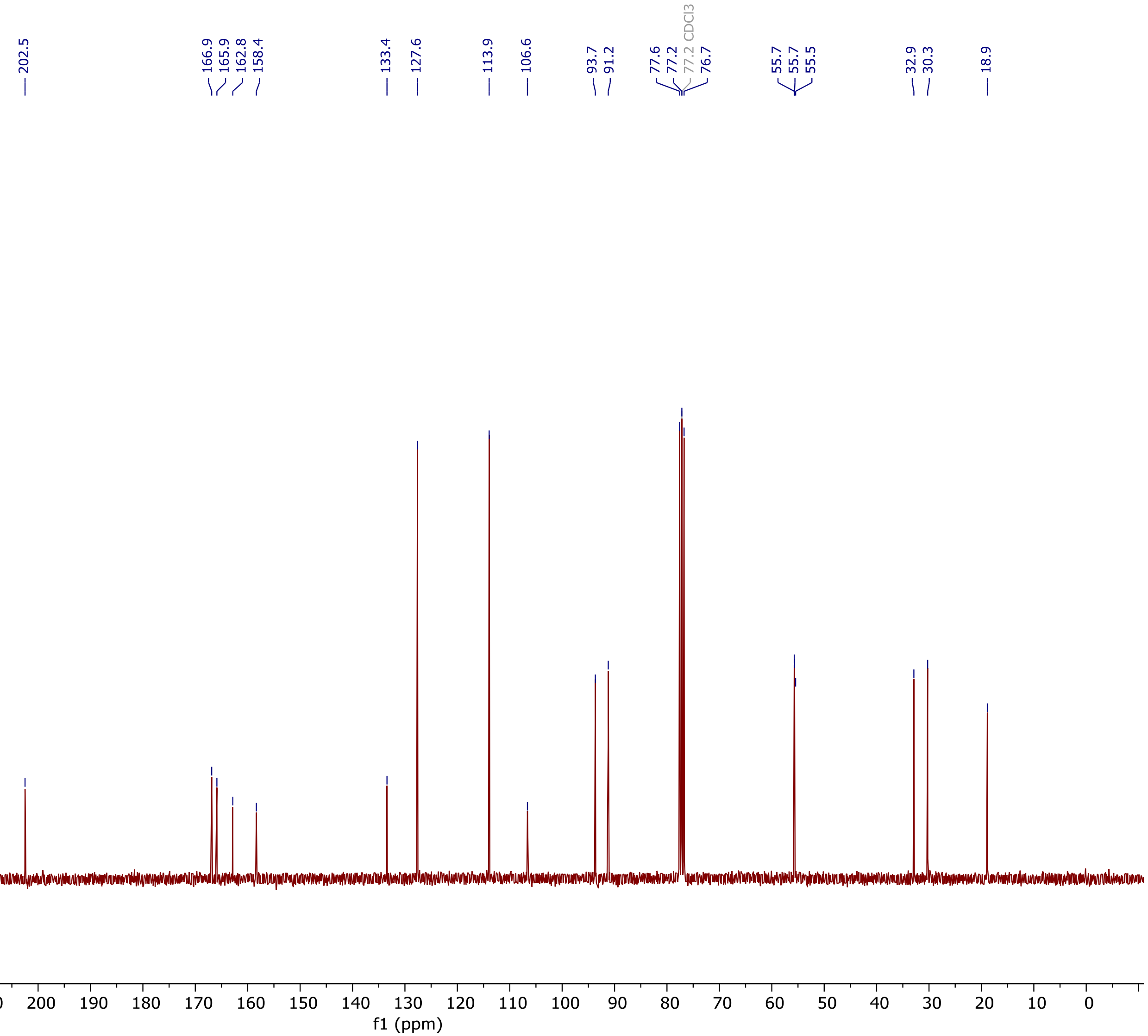

Compound 12 ^{1}H NMR in $CDCl_3$, 298 K, 300 MHz

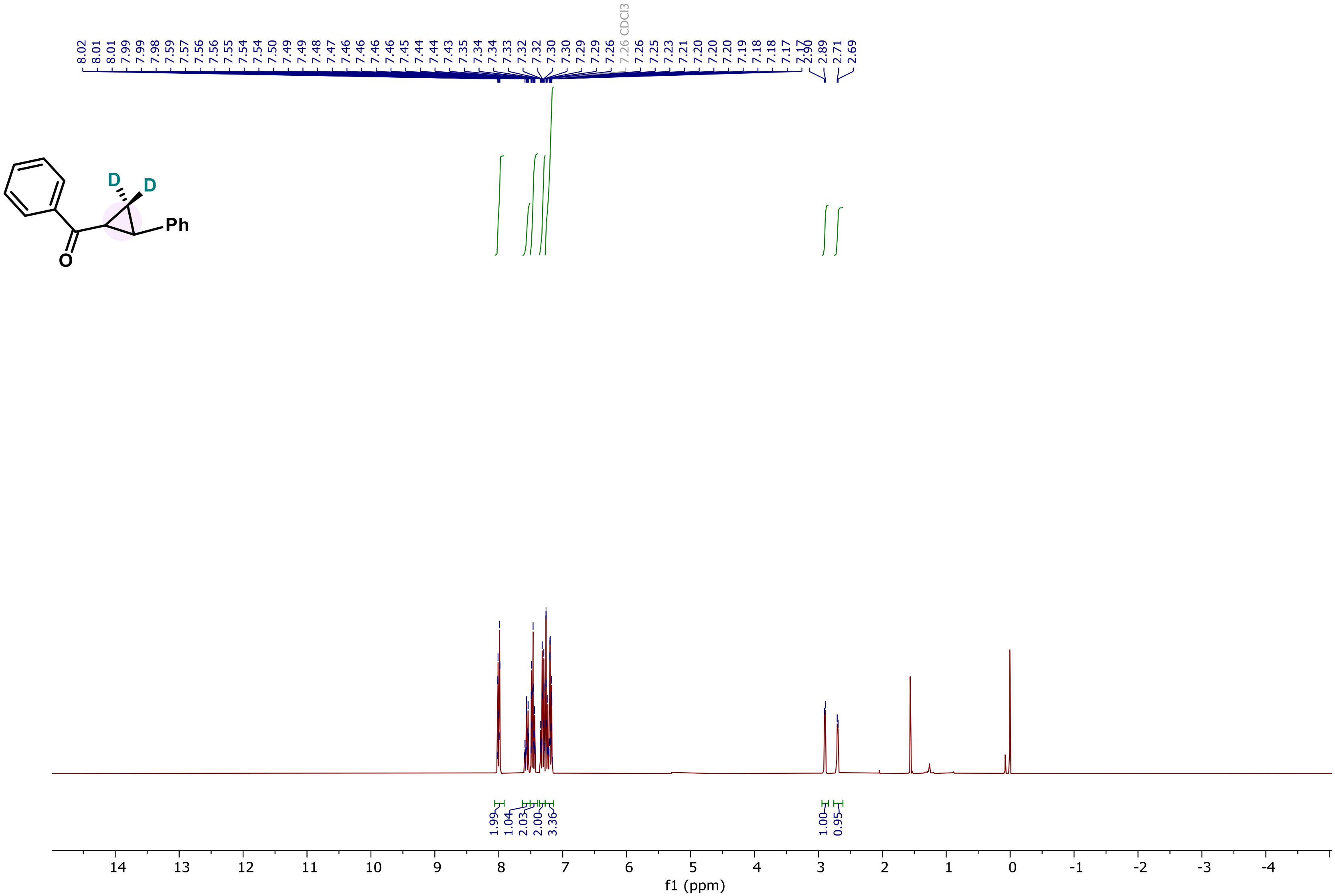

Compound 12 ^{13}C NMR in $CDCl_3$, 298 K, 151 MHz

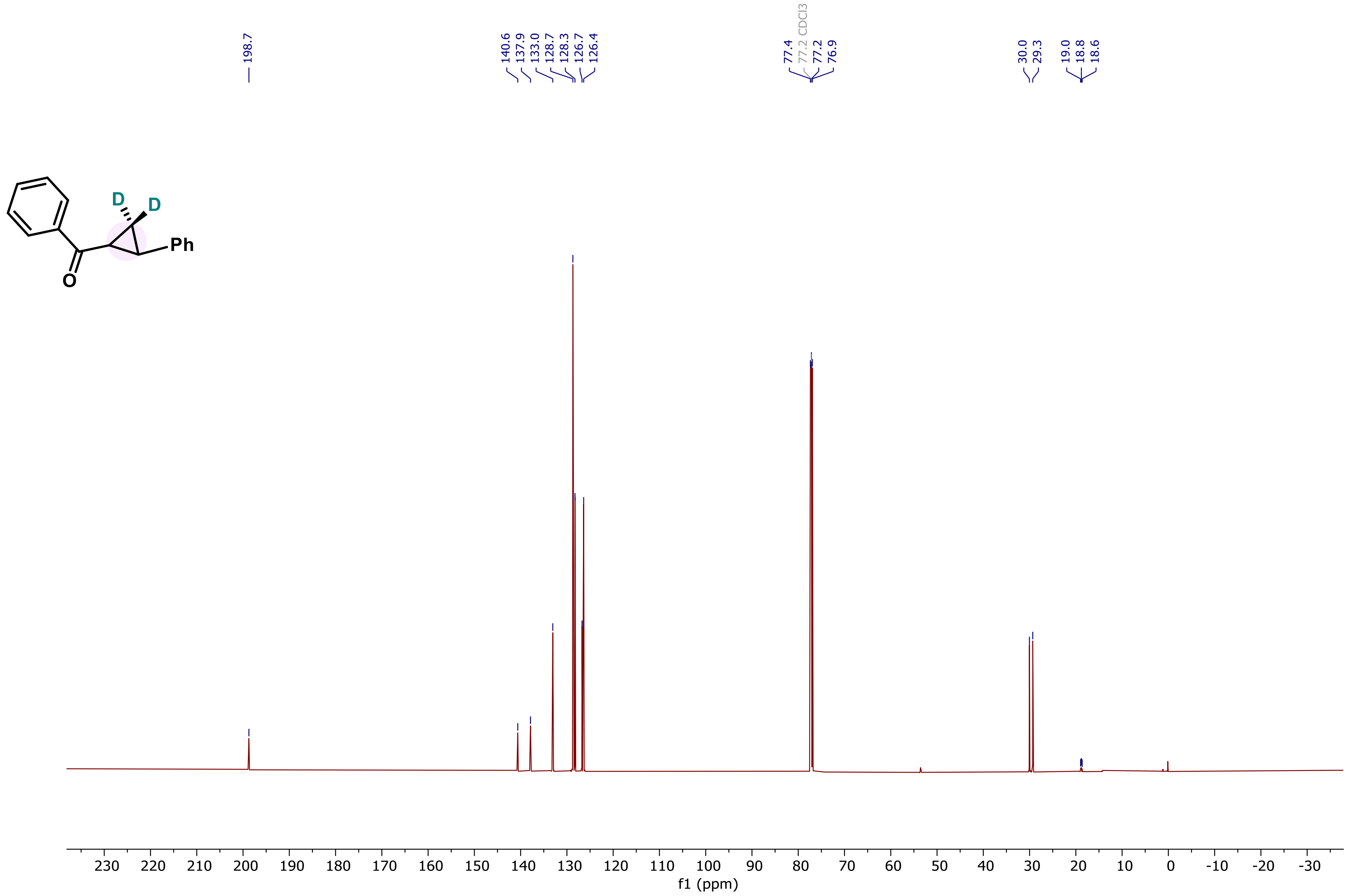

Compound 13 ^{1}H NMR in $CDCl_3$, 298 K, 300 MHz

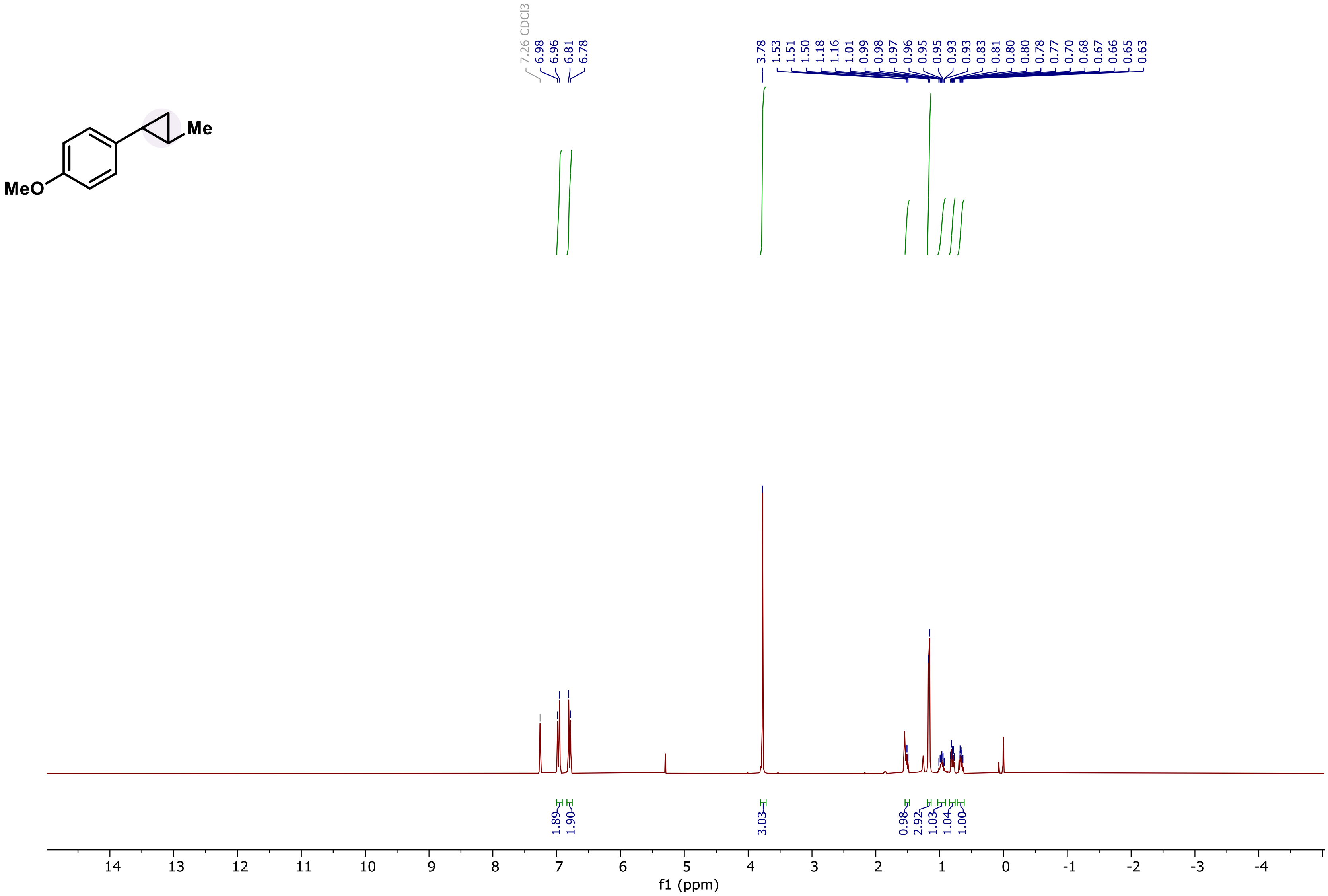

Compound 13 ^{13}C NMR in $CDCl_3$, 298 K, 75 MHz

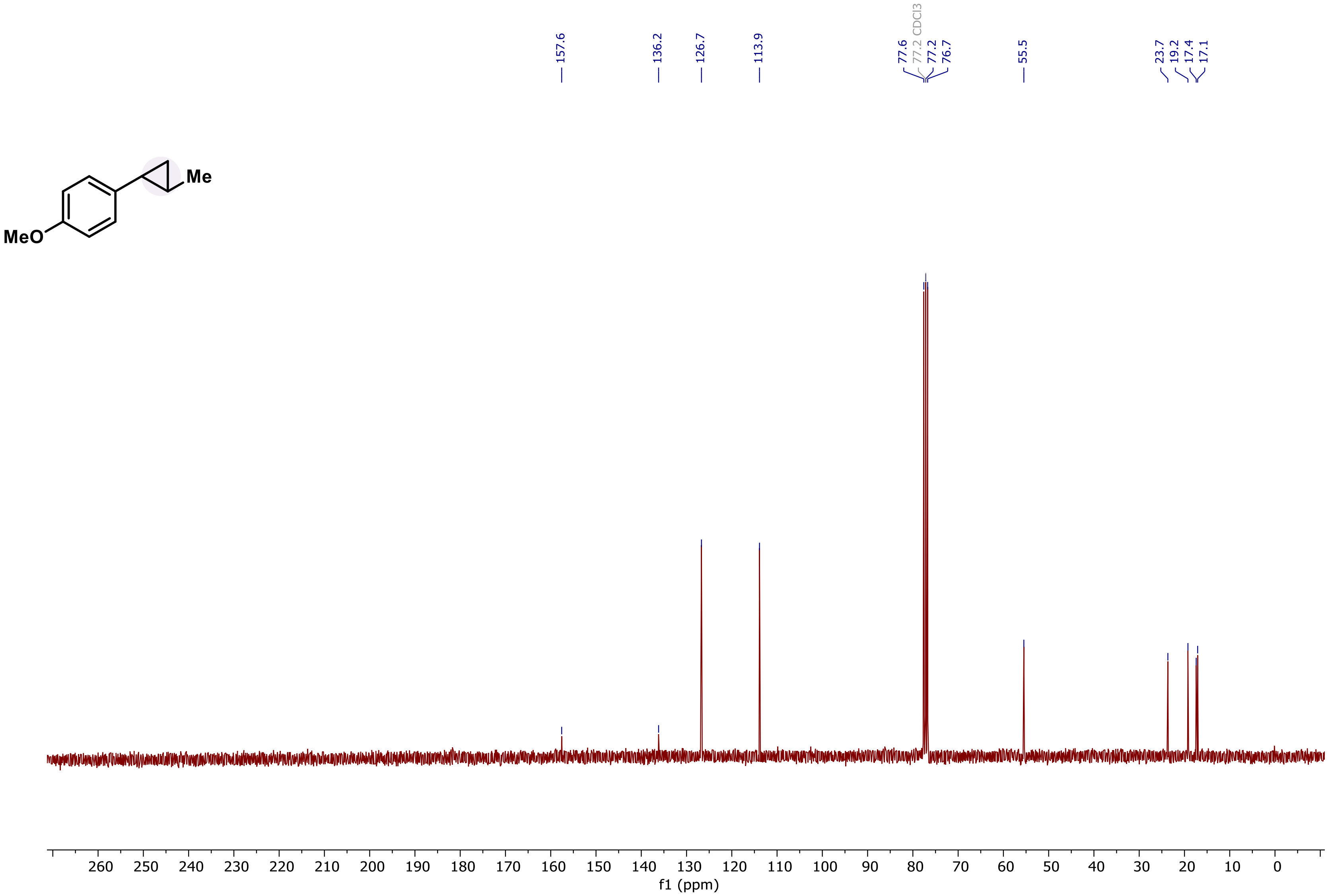

Compound 14 ^{1}H NMR in $CDCl_3$, 298 K, 300 MHz

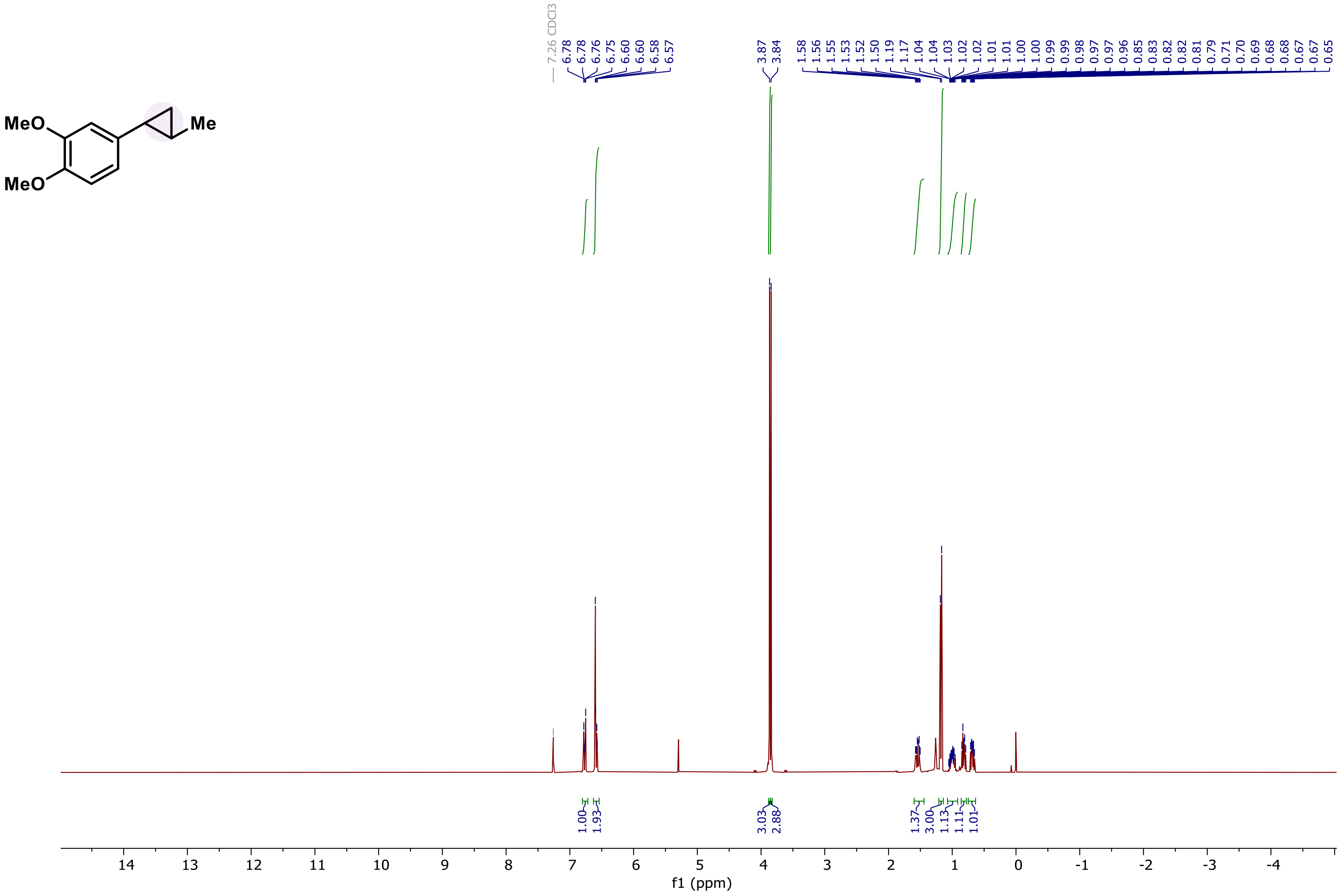

Compound 14 ^{13}C NMR in $CDCl_3$, 298 K, 75 MHz

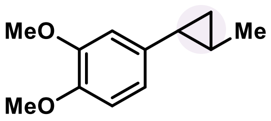

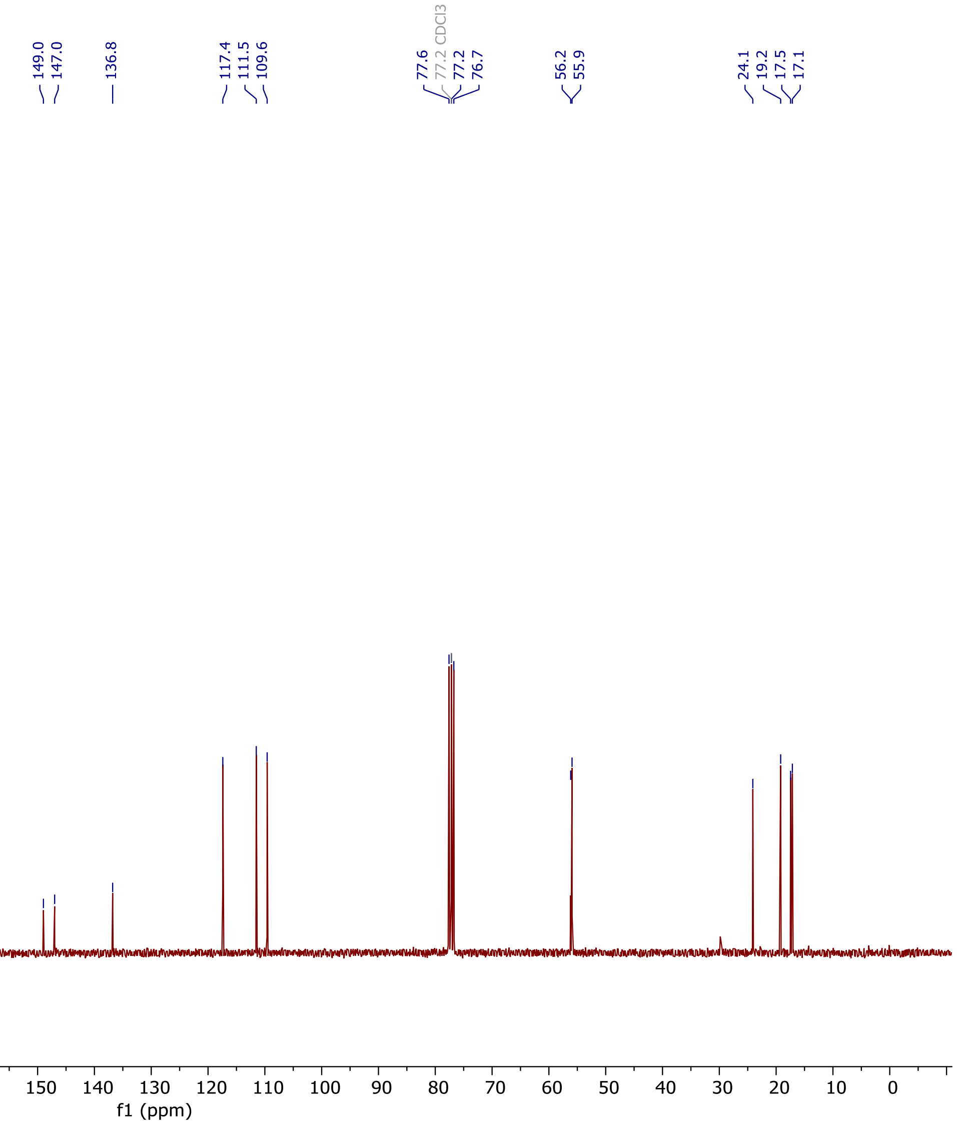

Compound 15 ^{1}H NMR in $CDCl_3$, 298 K, 300 MHz

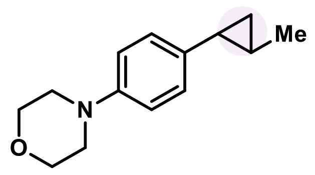

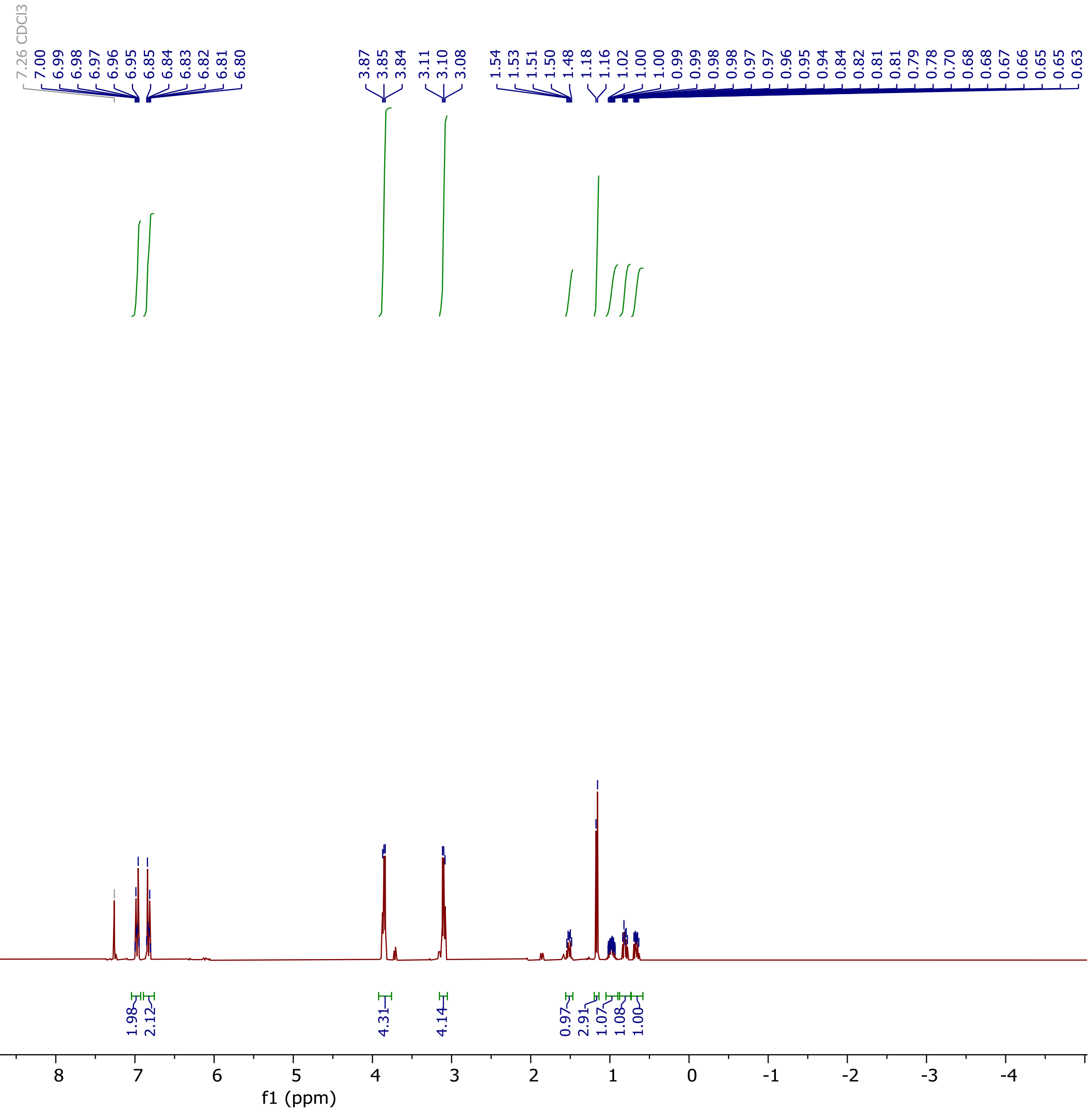

Compound 15 ^{13}C NMR in $CDCl_3$, 298 K, 75 MHz

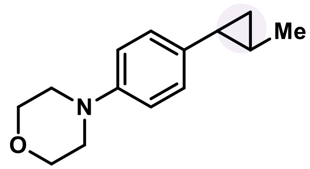

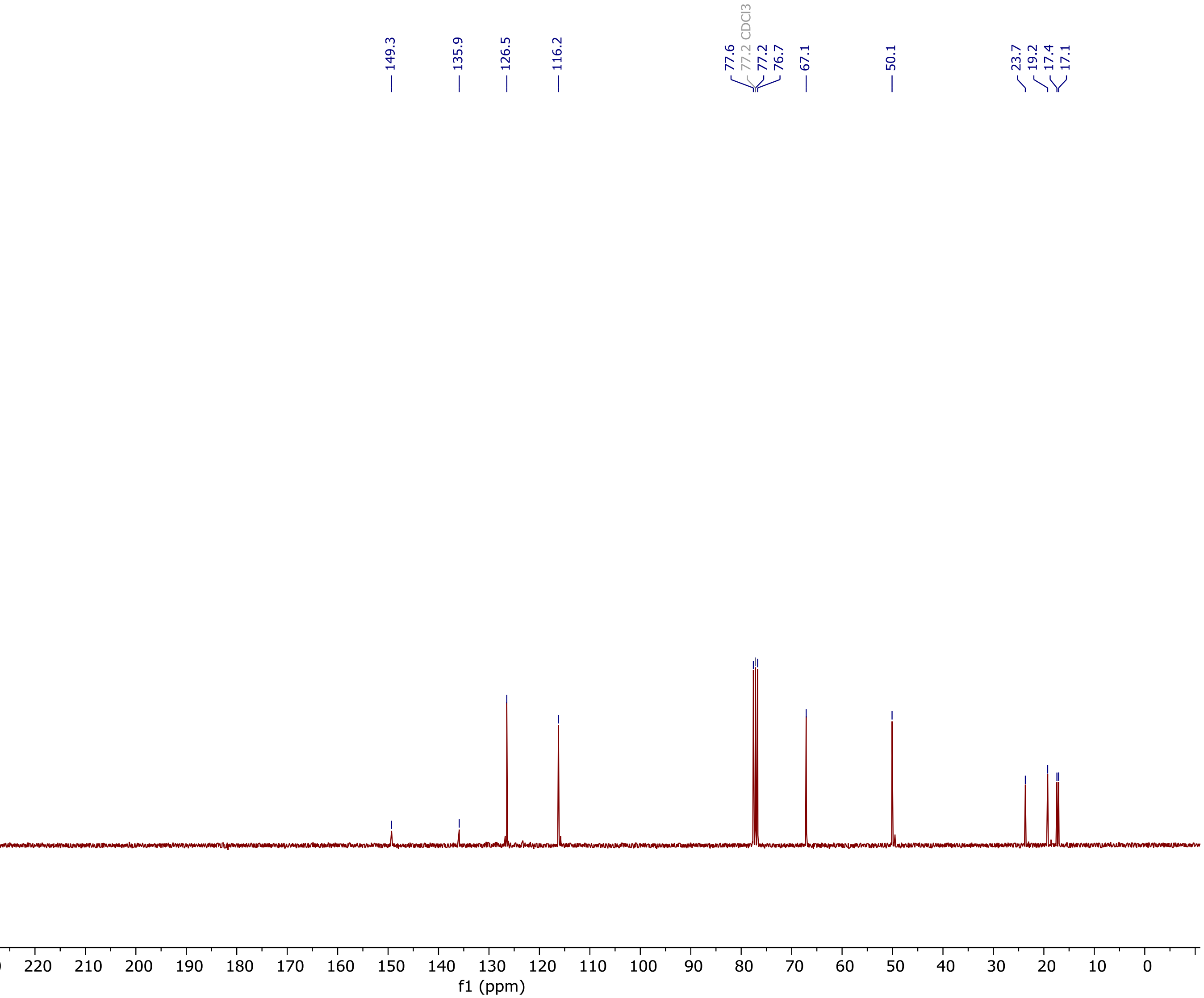

Compound 16 ^{1}H NMR in $CDCl_3$, 298 K, 300 MHz

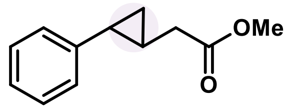

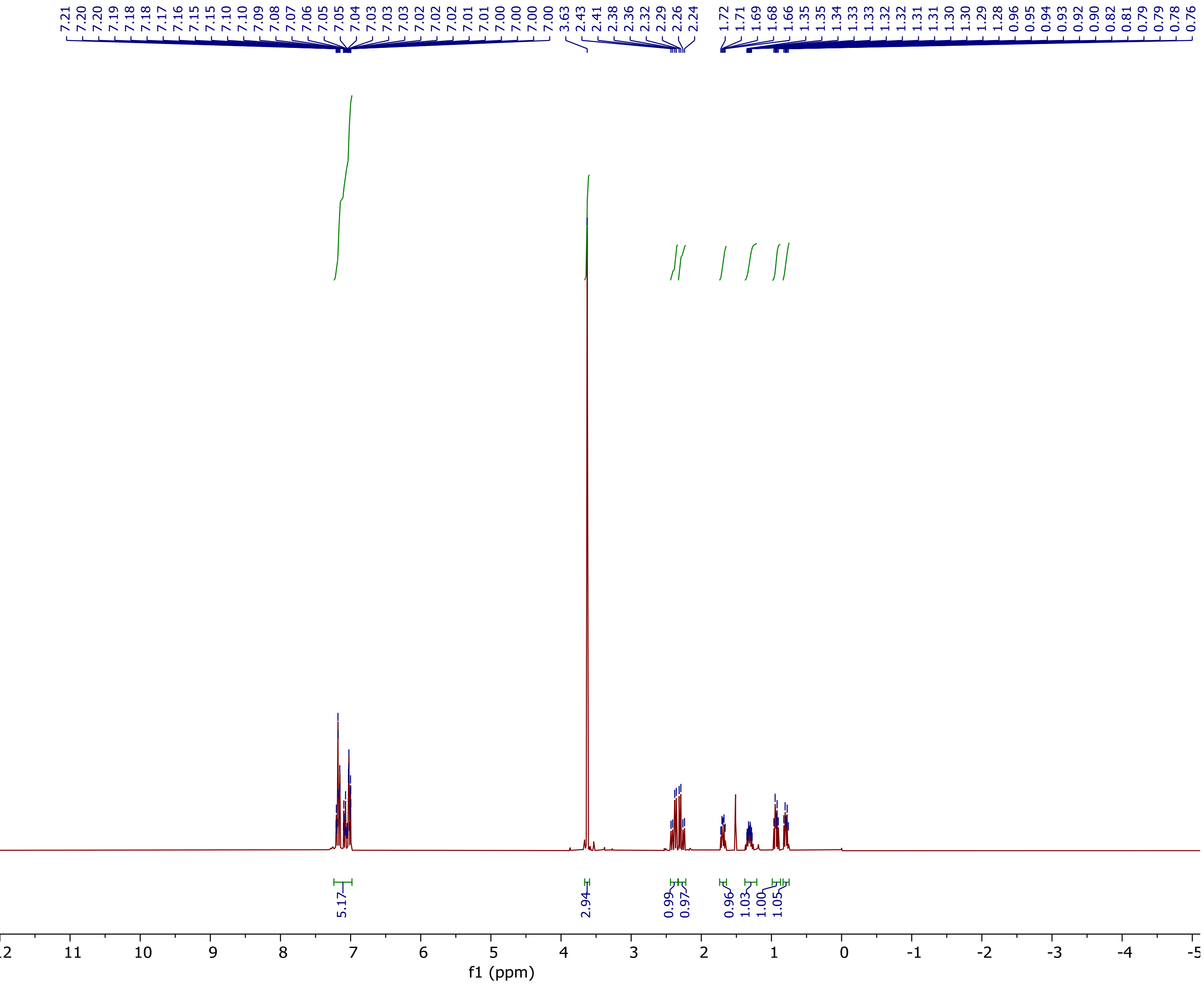

Compound 16 ^{13}C NMR in $CDCl_3$, 298 K, 75 MHz

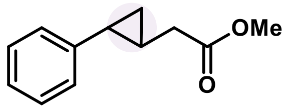

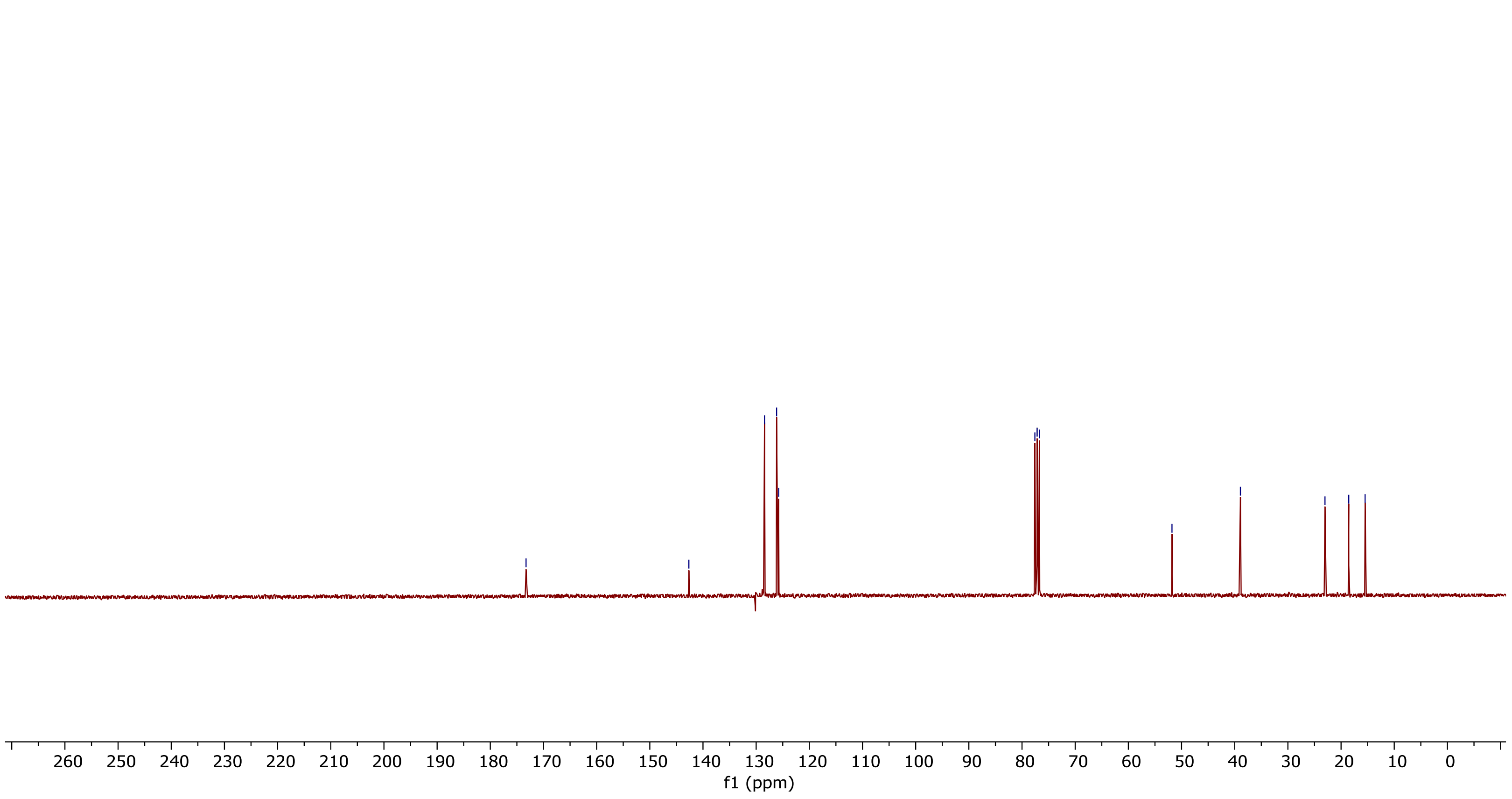

Compound 17 ^{1}H NMR in $CDCl_3$, 298 K, 300 MHz

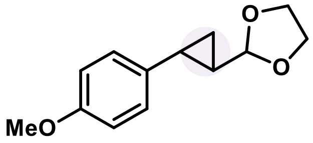

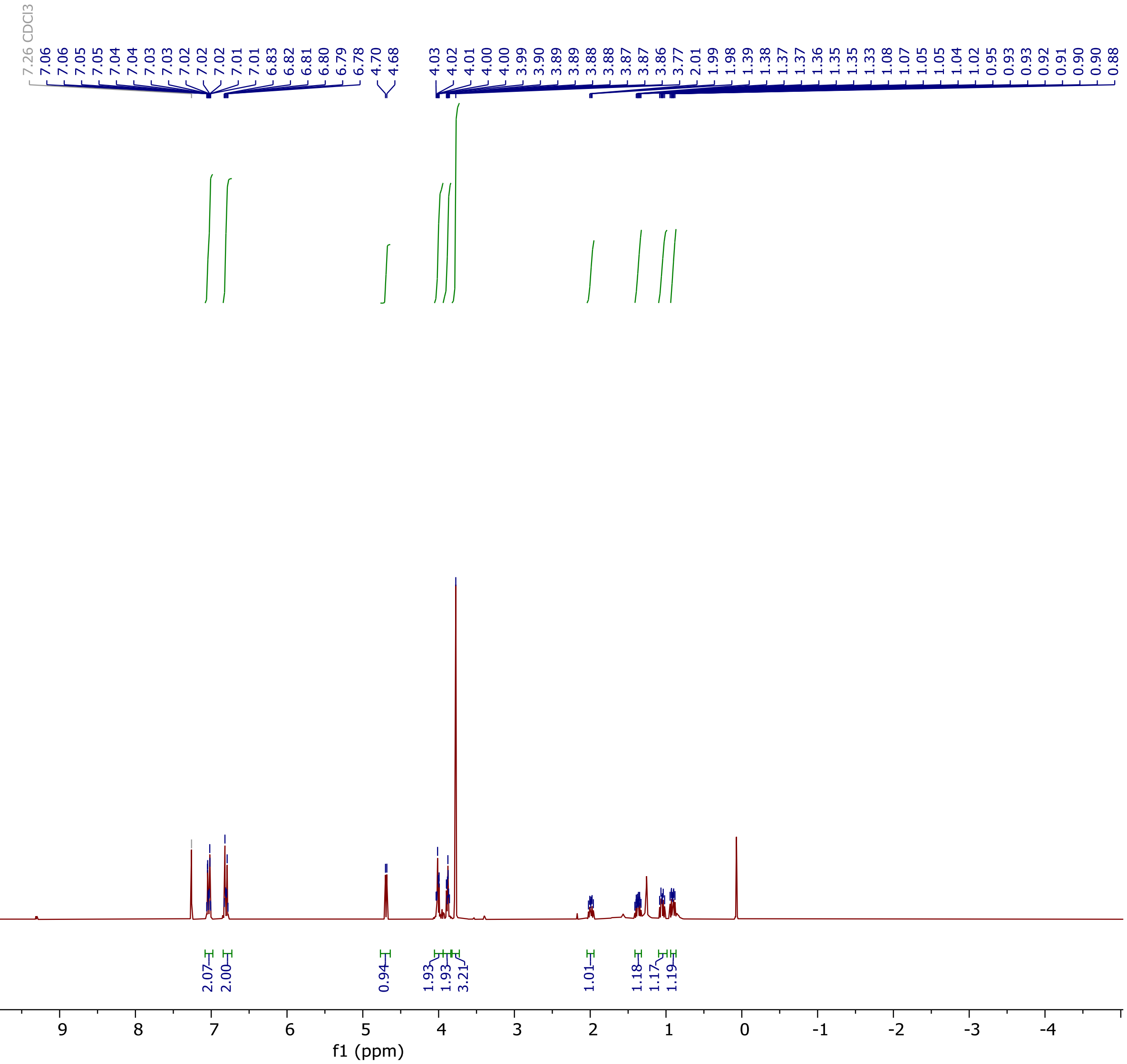

Compound 17 ^{13}C NMR in $CDCl_3$, 298 K, 75 MHz

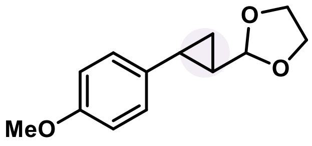

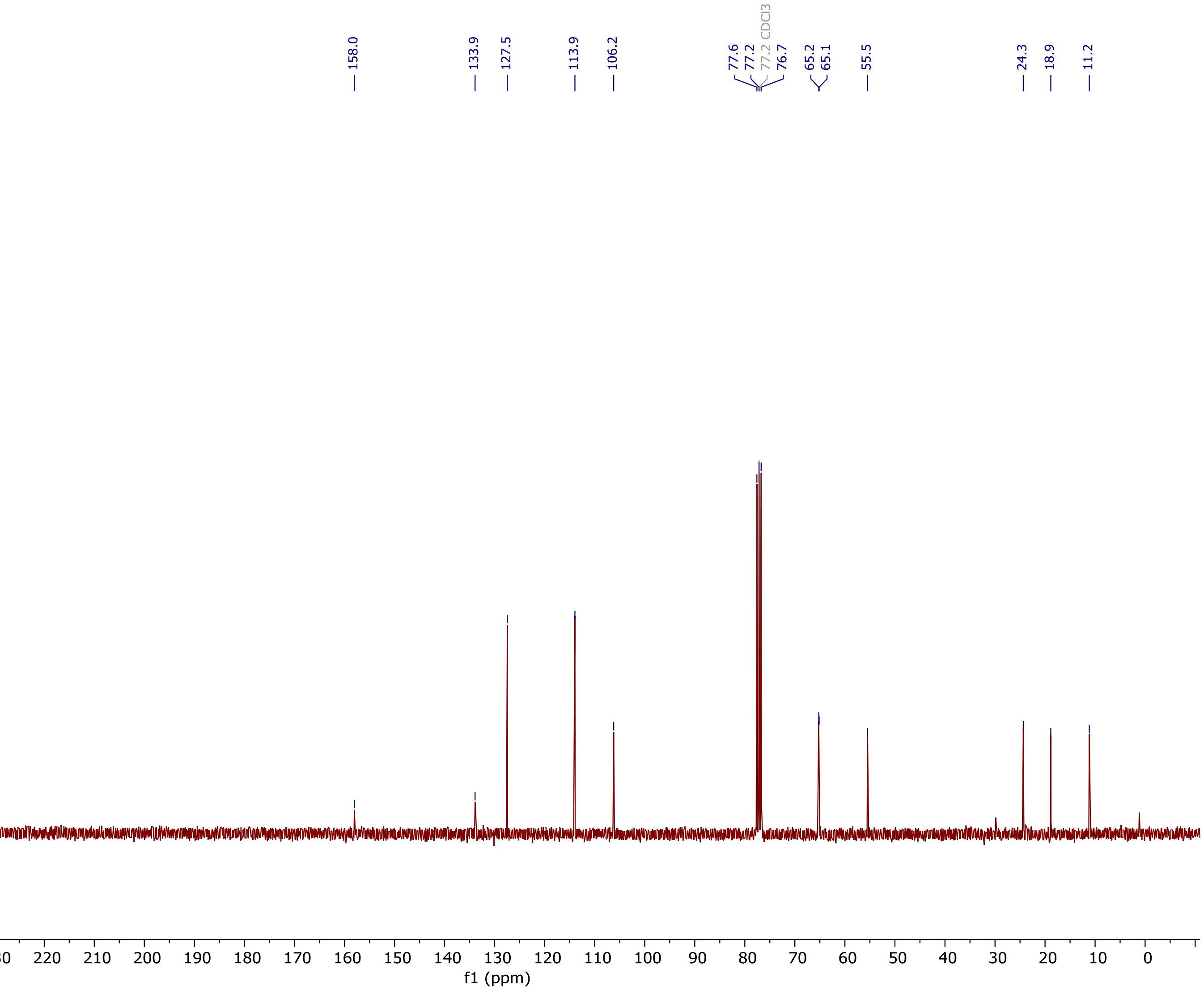

Compound 18 ¹H NMR in $CDCl_3$, 298 K, 300 MHz

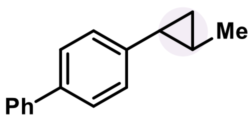

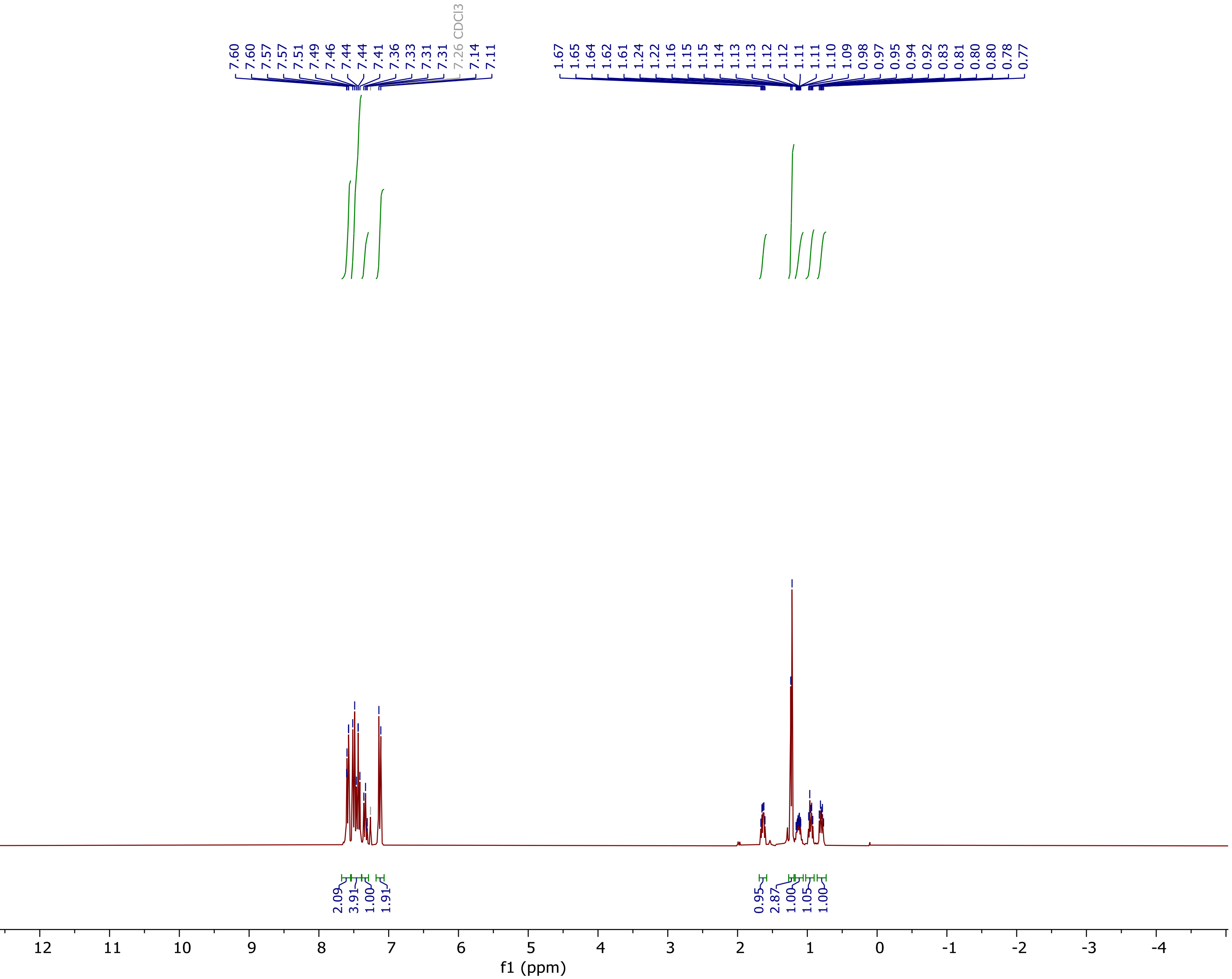

Compound 18 ^{13}C NMR in $CDCl_3$, 298 K, 75 MHz

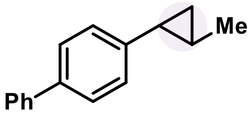

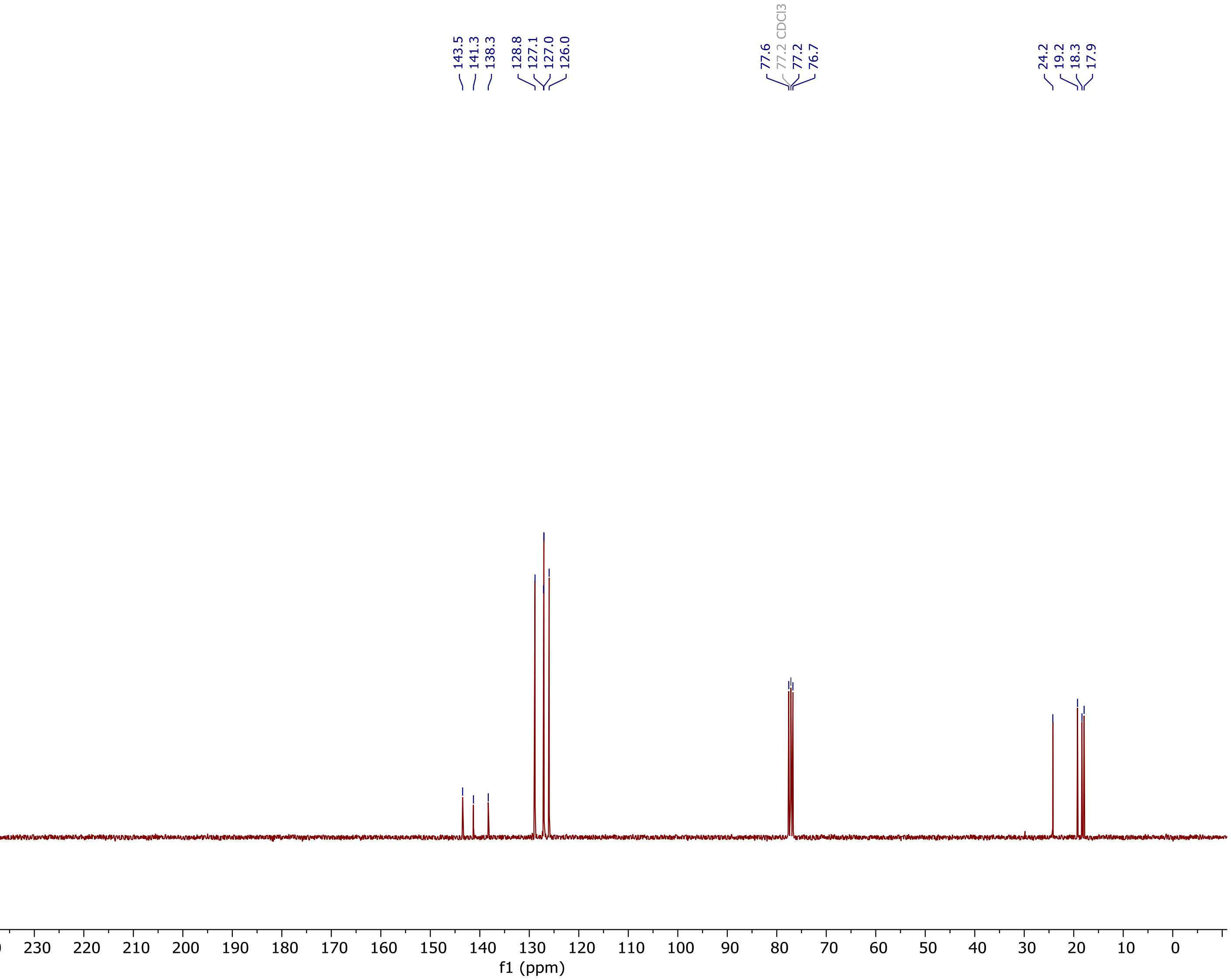

Compound 19 ^{1}H NMR in $CDCl_3$, 298 K, 300 MHz

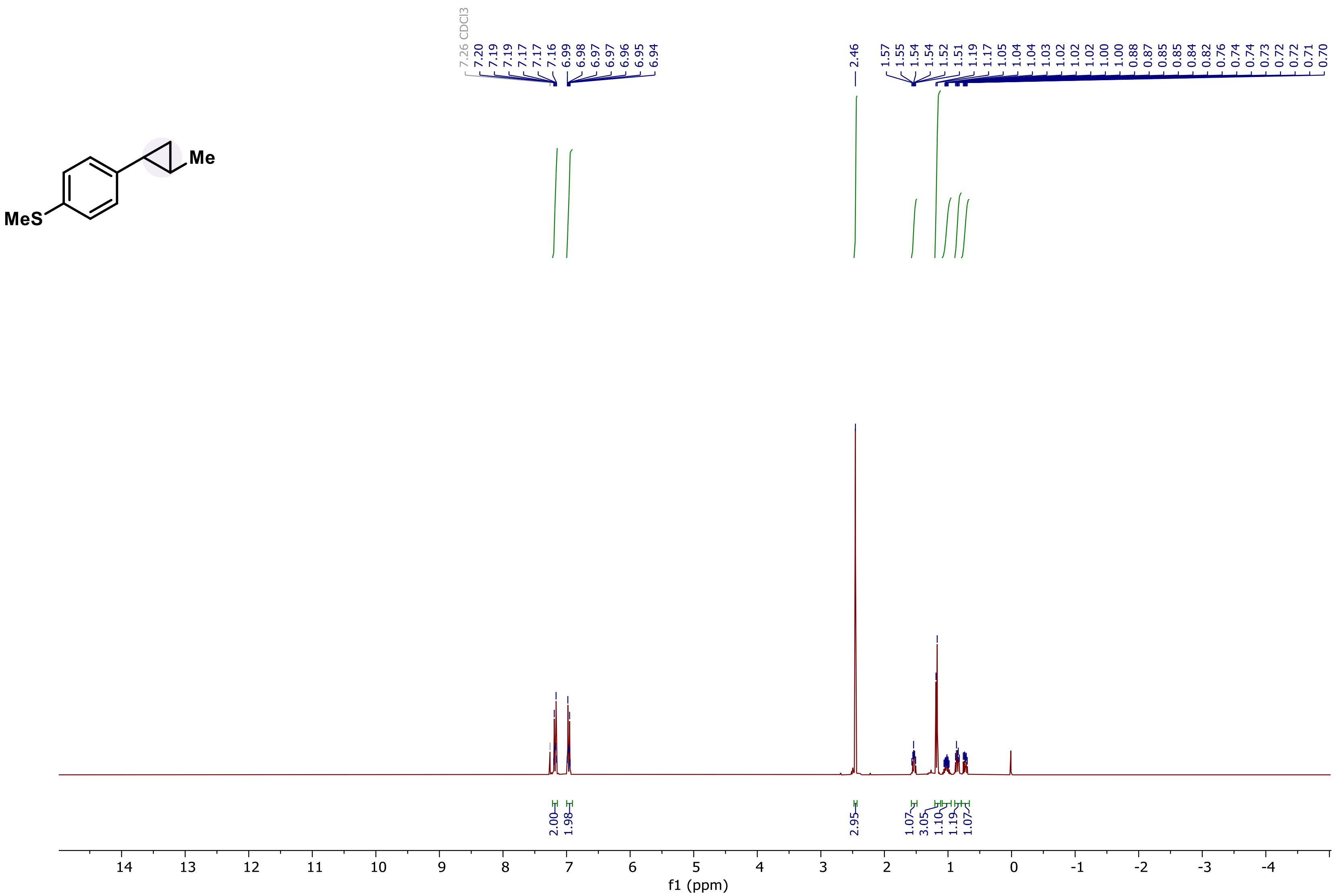

Compound 19 ^{13}C NMR in $CDCl_3$, 298 K, 75 MHz

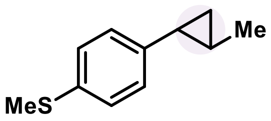

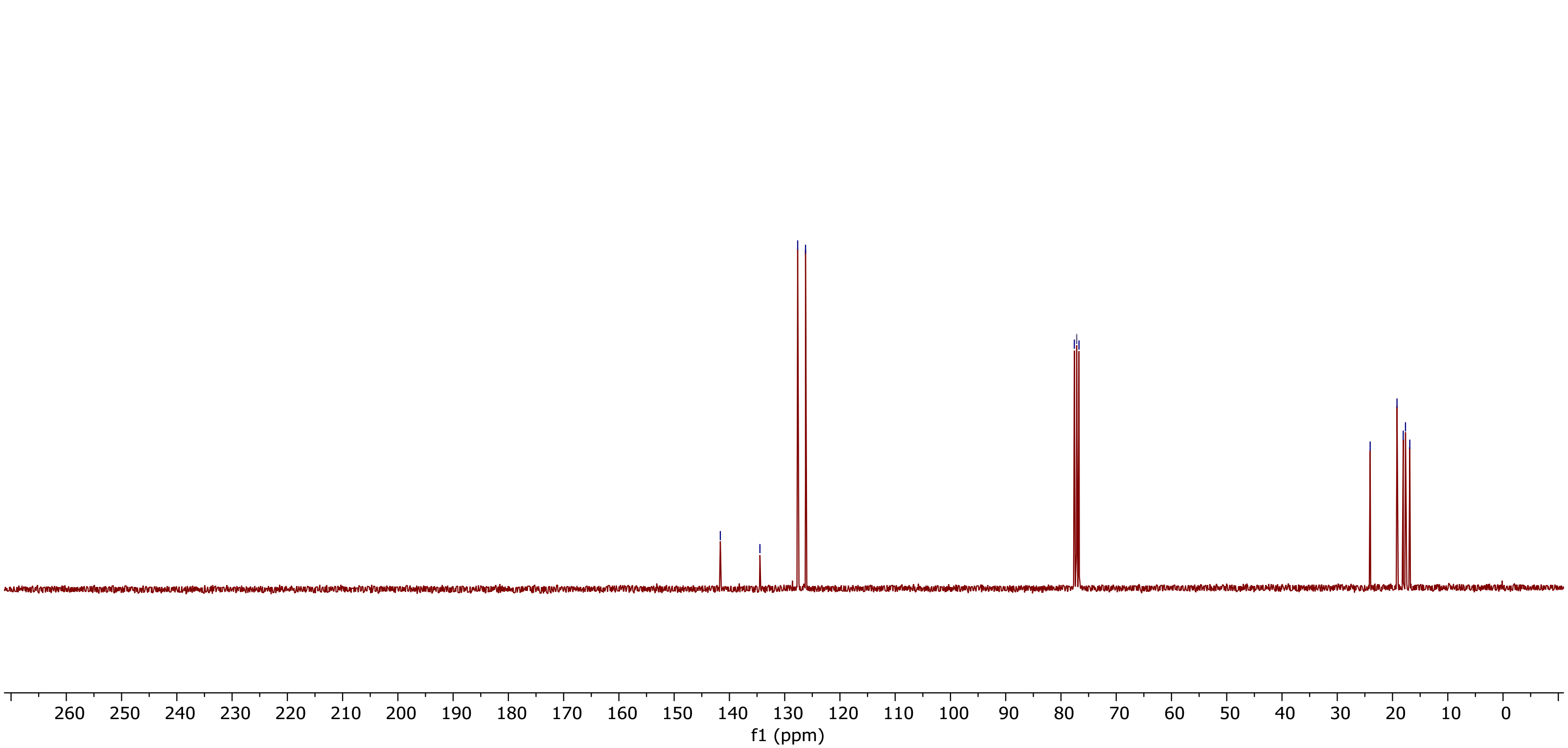

Compound 20 ^{1}H NMR in $CDCl_3$, 298 K, 300 MHz

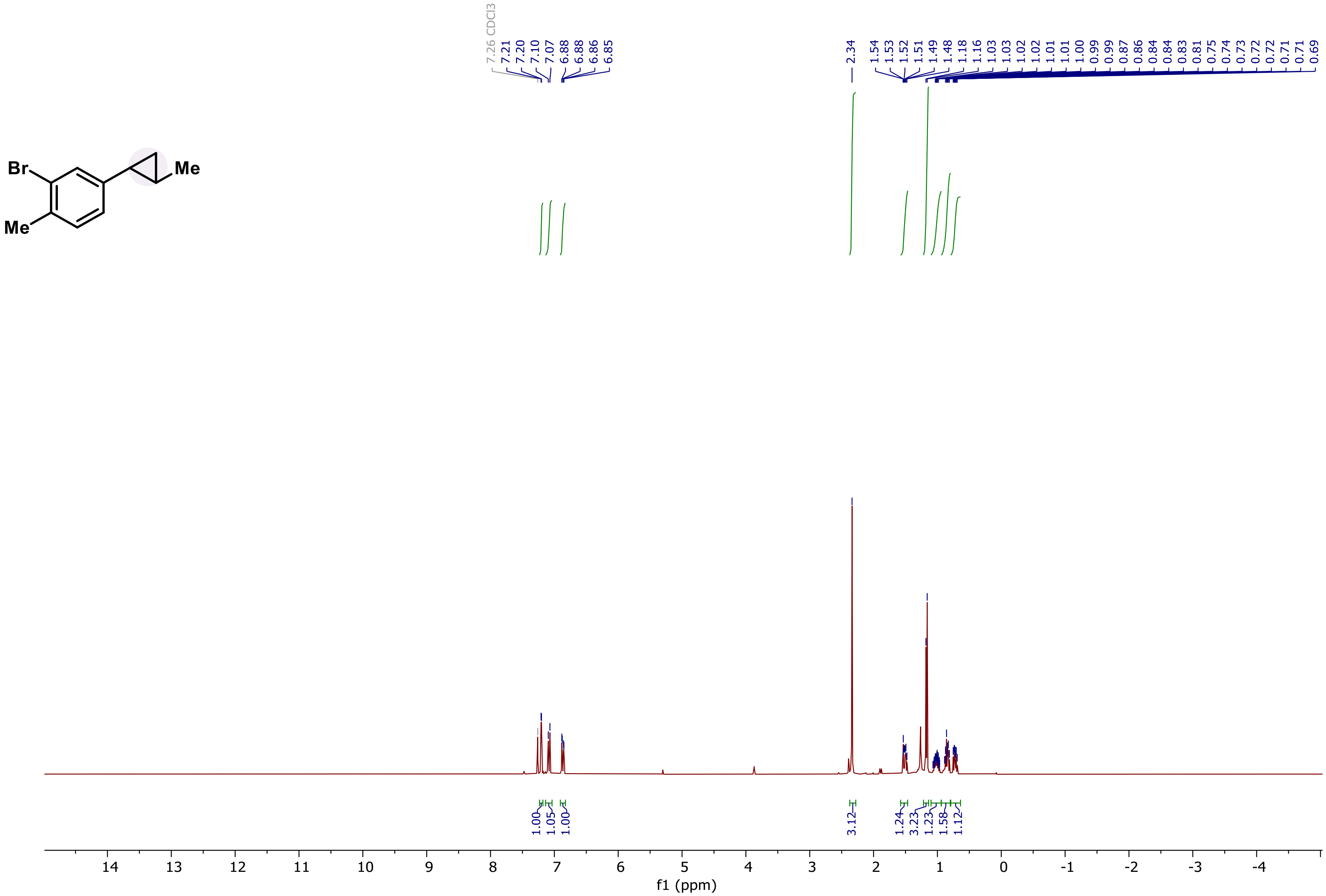

Compound 20 ^{13}C NMR in $CDCl_3$, 298 K, 75 MHz

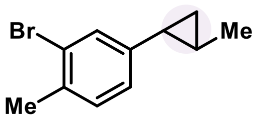

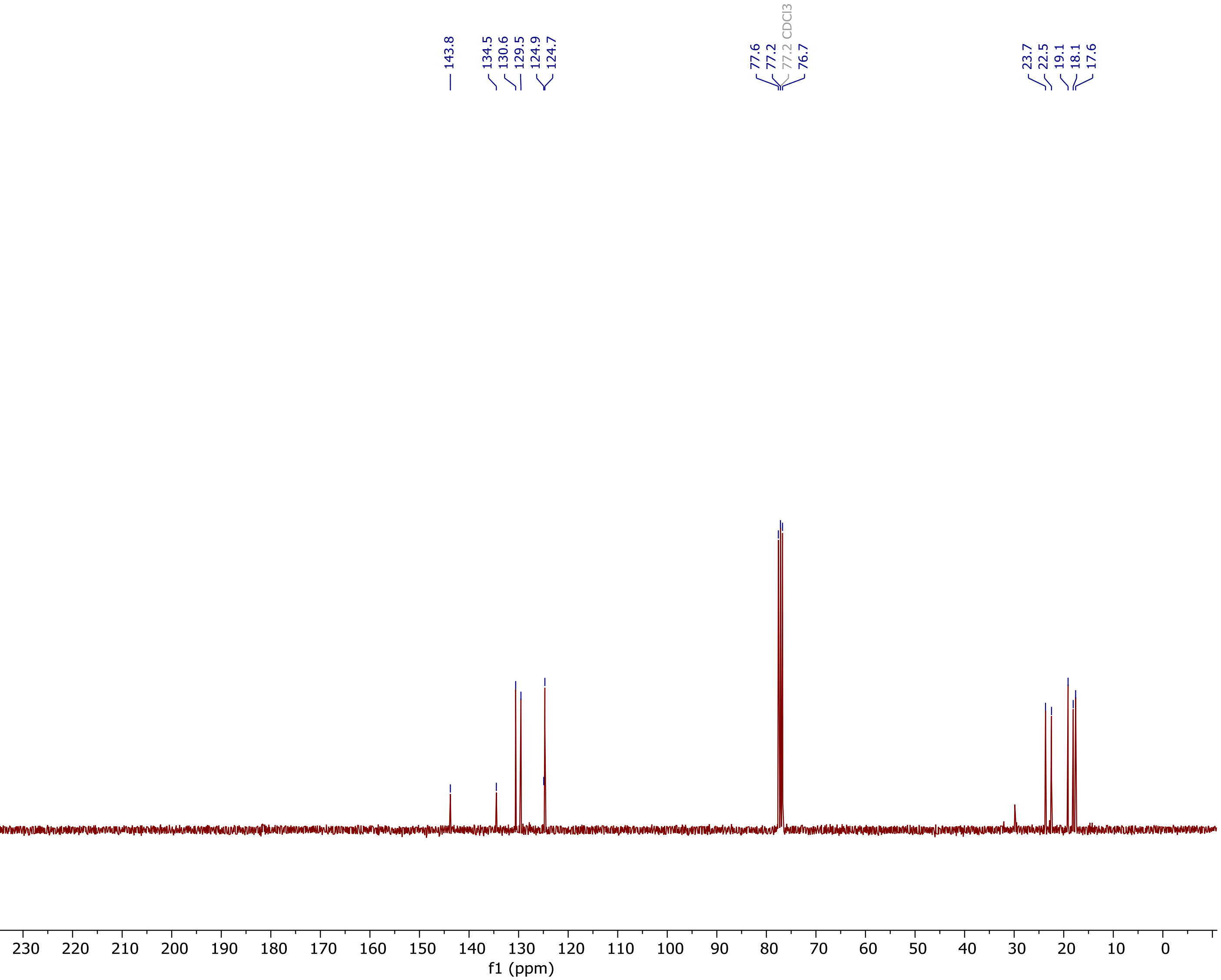

Compound 21 ^{1}H NMR in $CDCl_3$, 298 K, 300 MHz

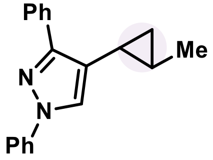

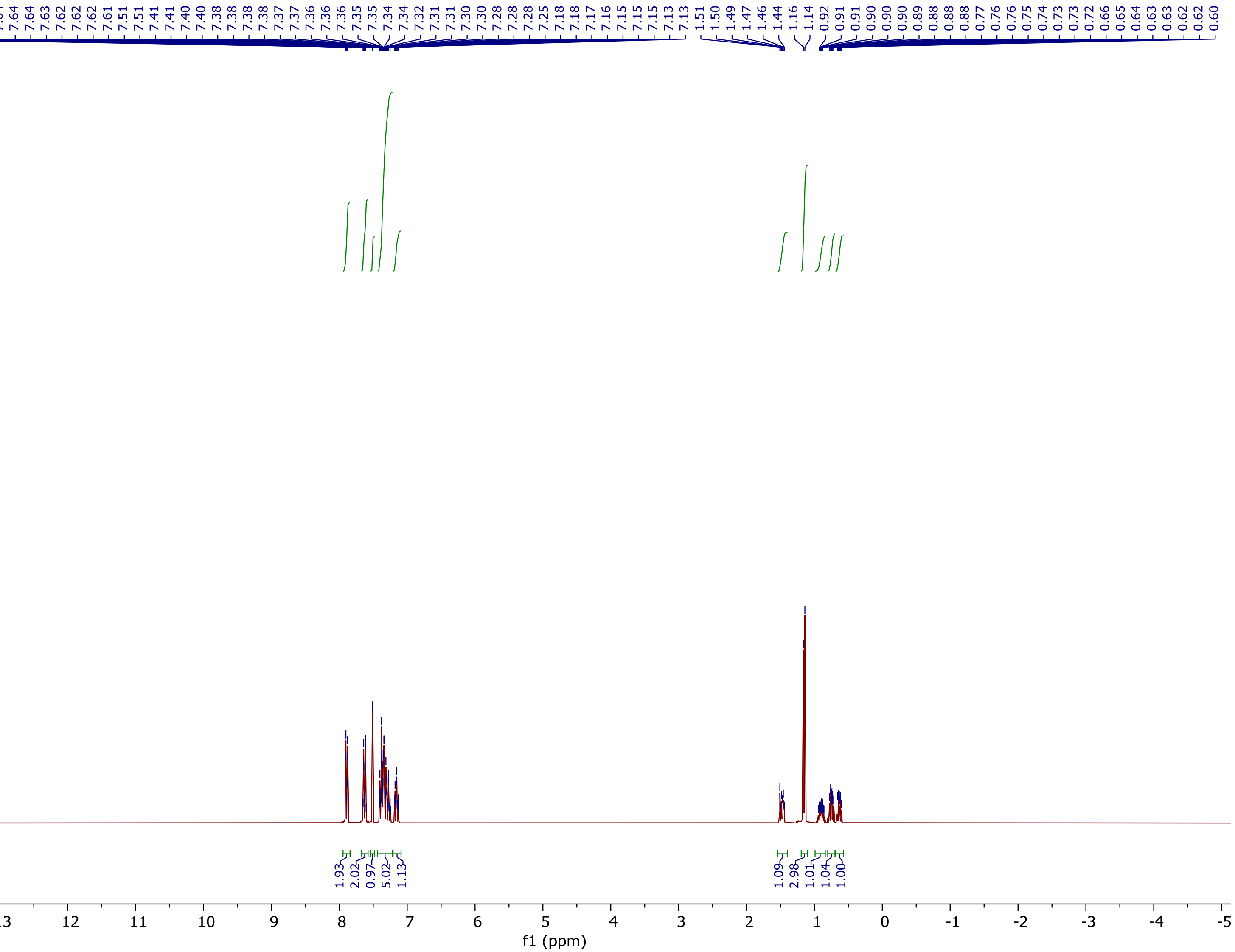

Compound 21 ^{13}C NMR in $CDCl_3$, 298 K, 75 MHz

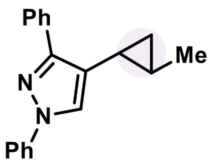

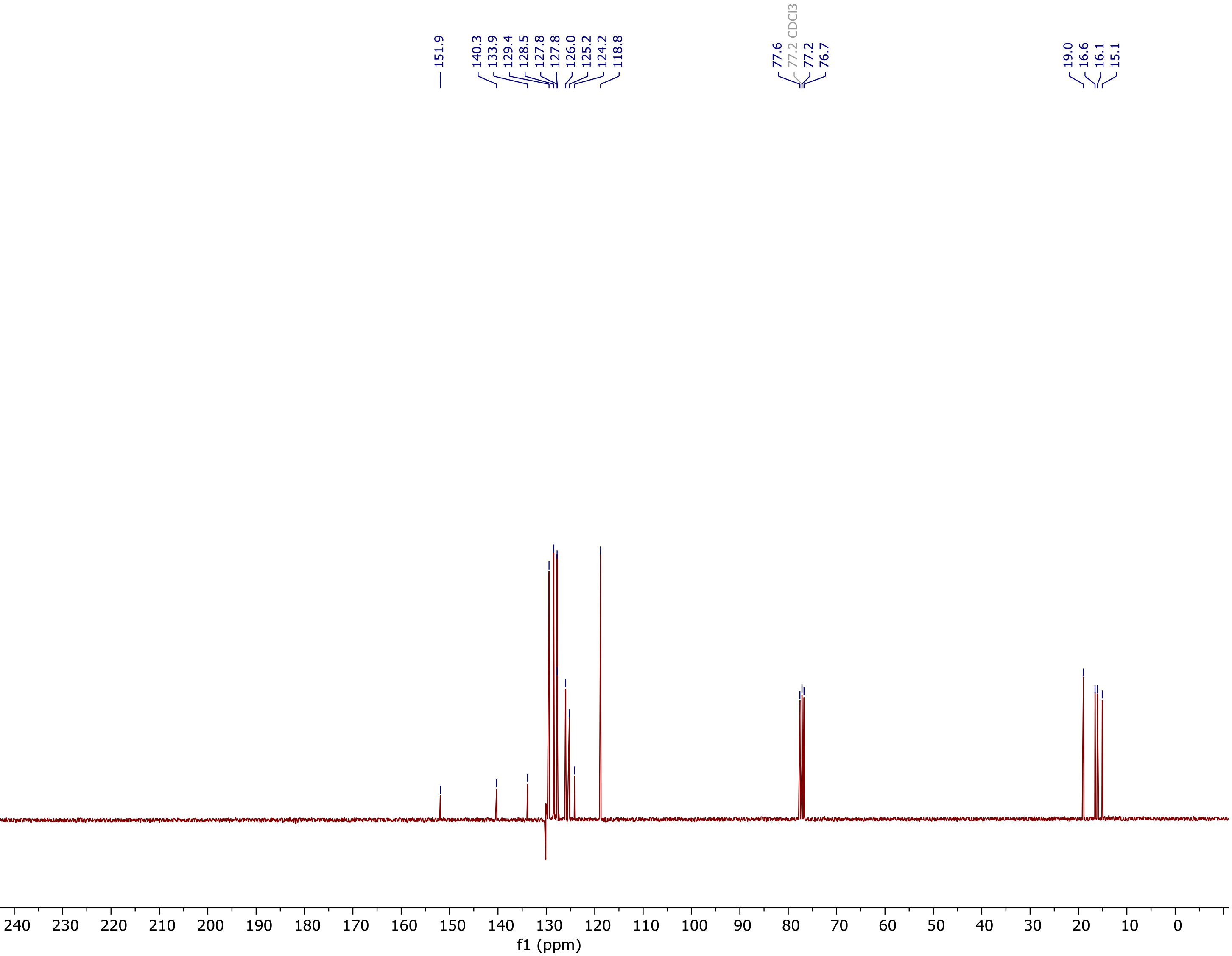

Compound 22 ^{1}H NMR in $CDCl_3$, 298 K, 300 MHz

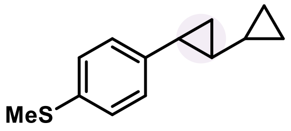

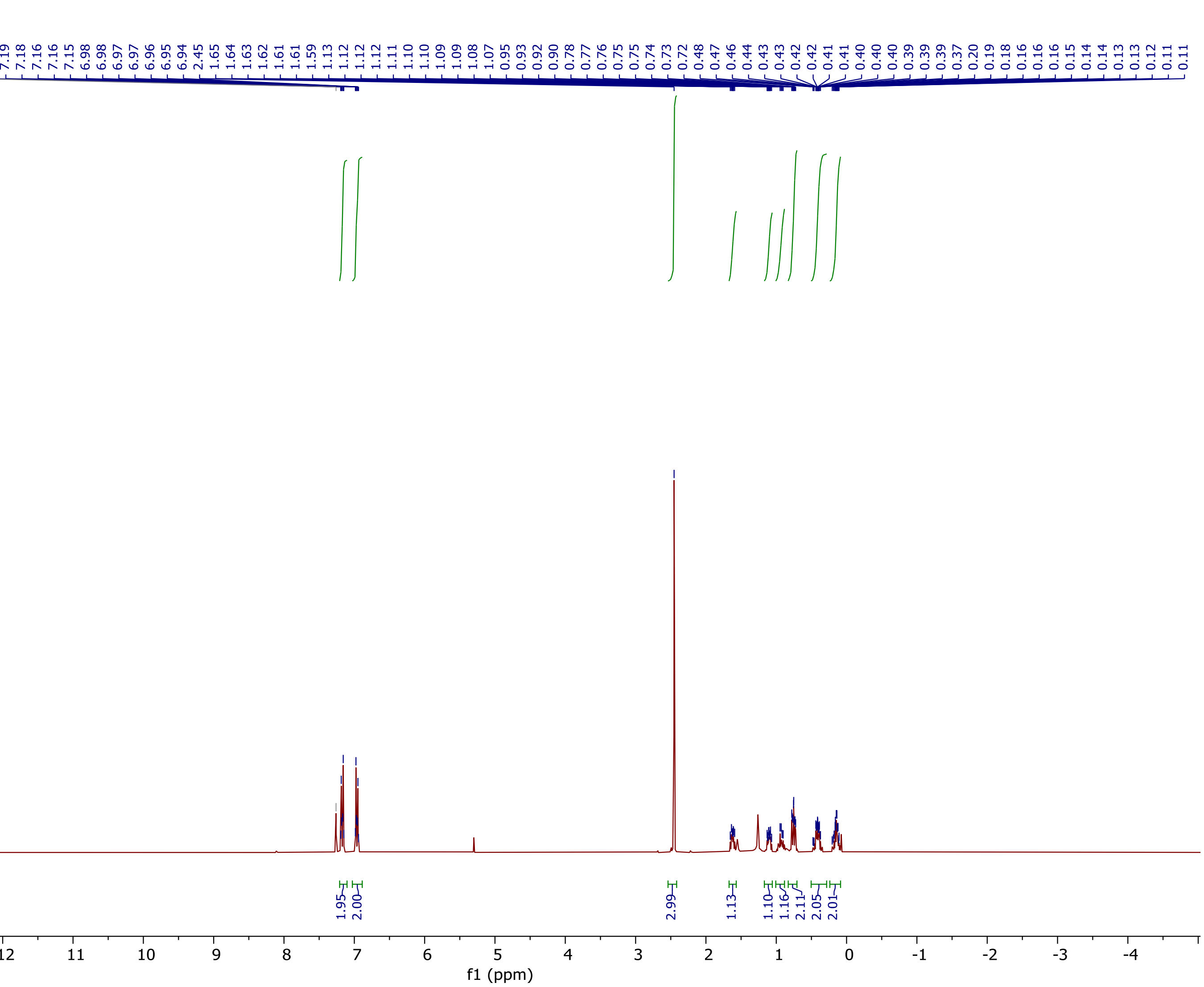

Compound 22 ^{13}C NMR in $CDCl_3$, 298 K, 75 MHz

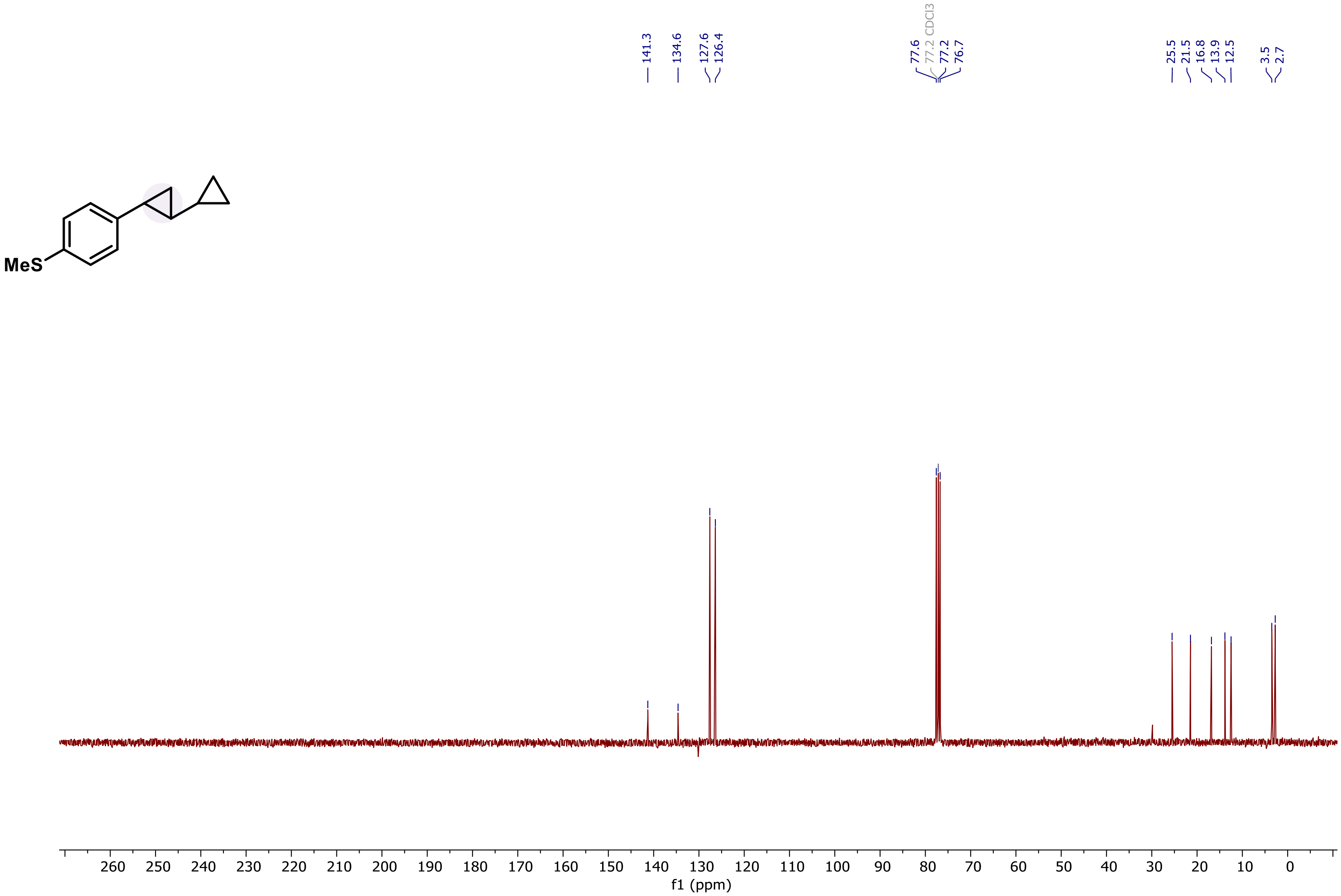

Compound 23 ^{1}H NMR in $CDCl_3$, 298 K, 600 MHz

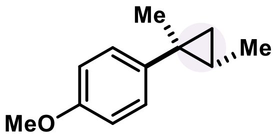

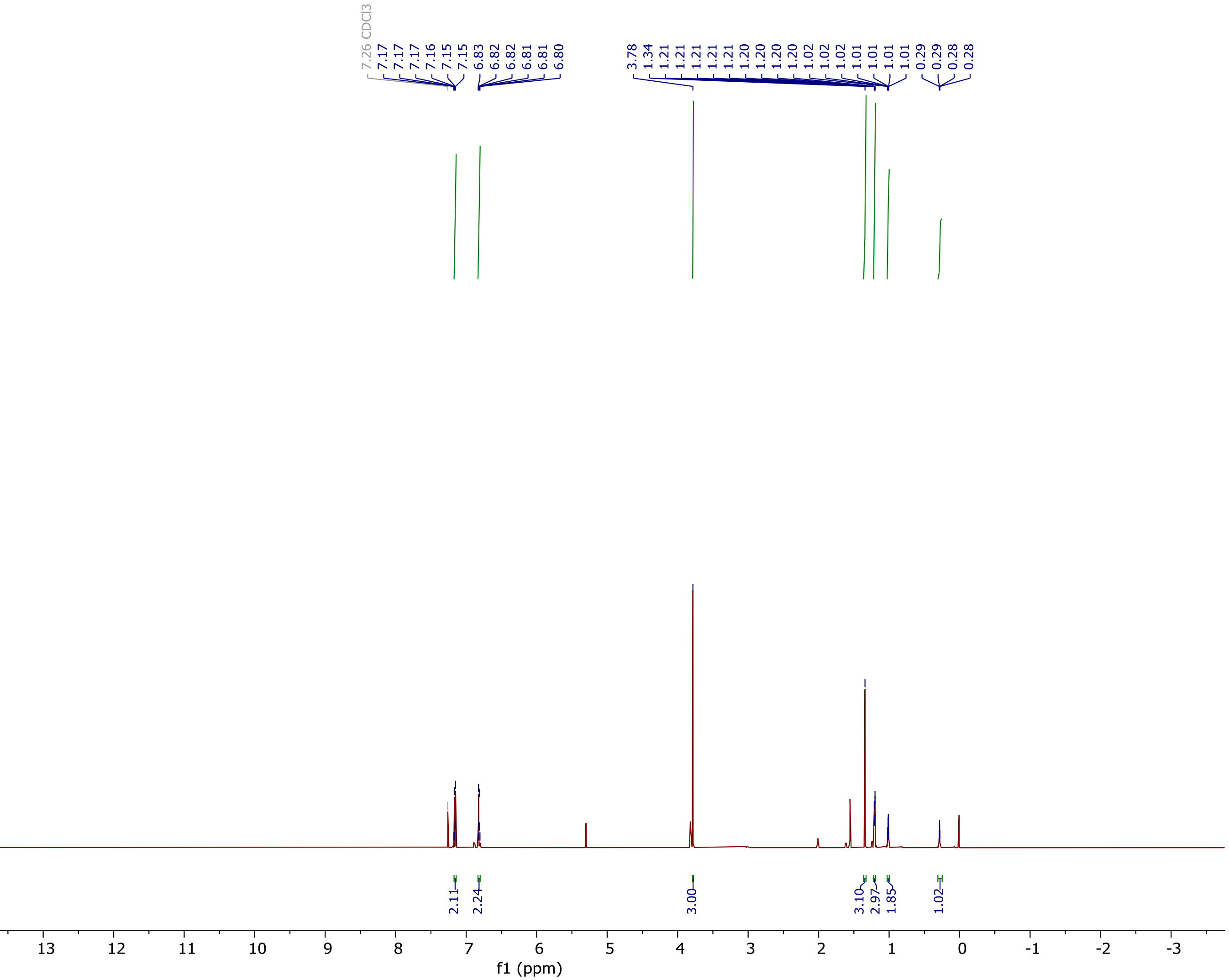

Compound 23 ^{13}C NMR in $CDCl_3$, 298 K, 151 MHz

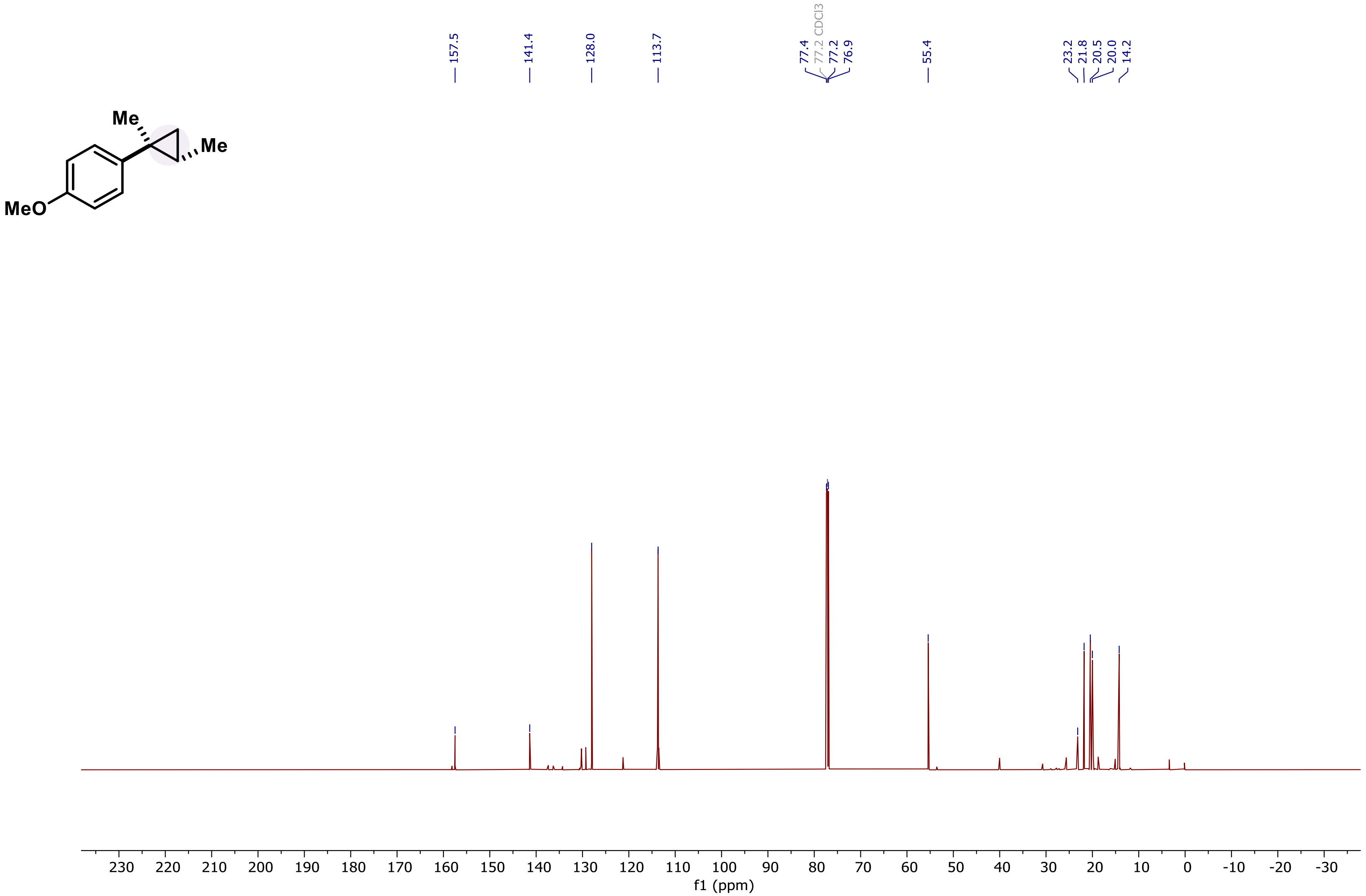

Compound 30 ^{1}H NMR in $CDCl_3$, 298 K, 300 MHz

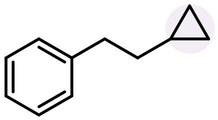

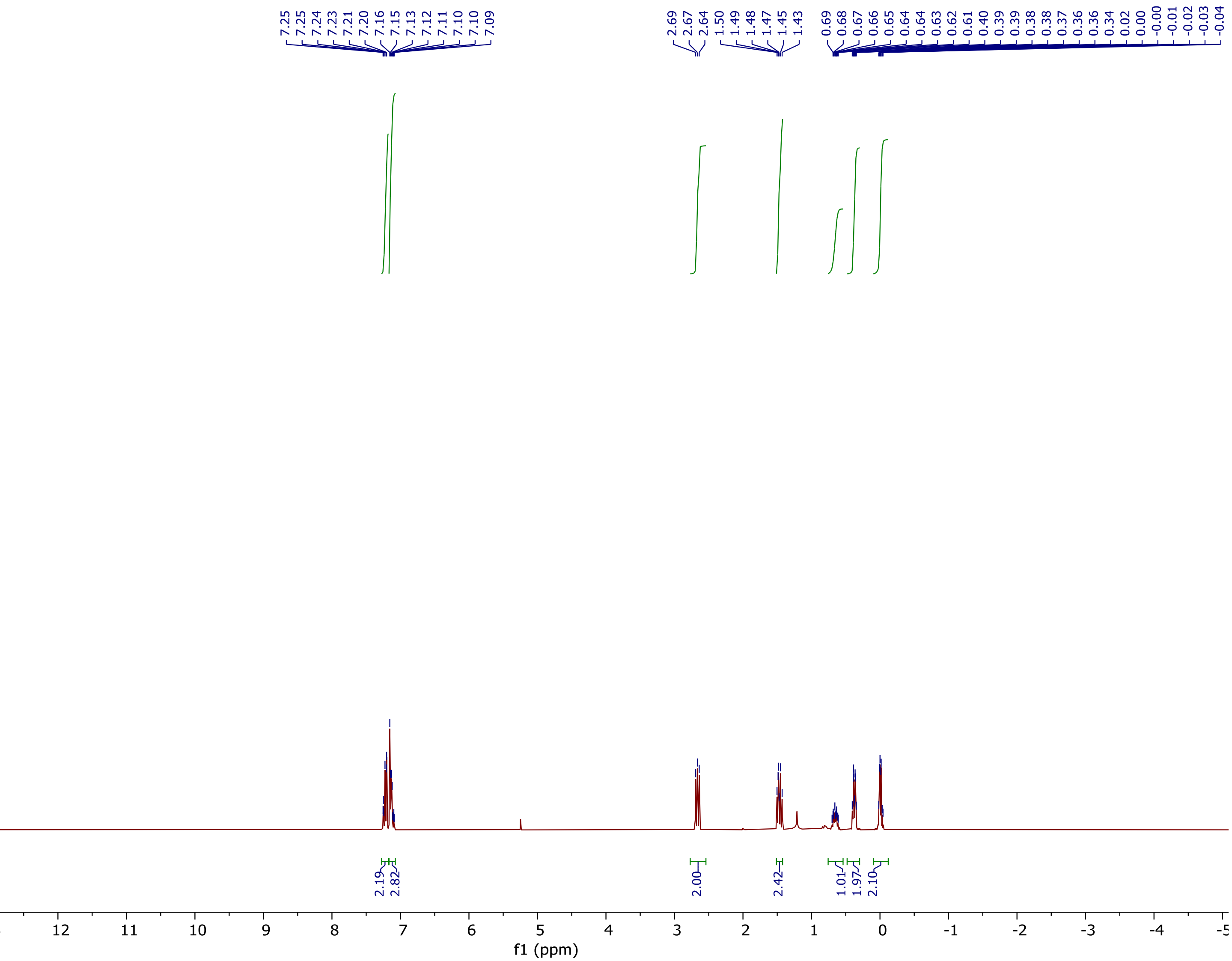

Compound 30 ^{13}C NMR in $CDCl_3$, 298 K, 75 MHz

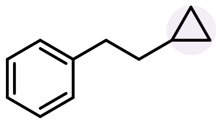

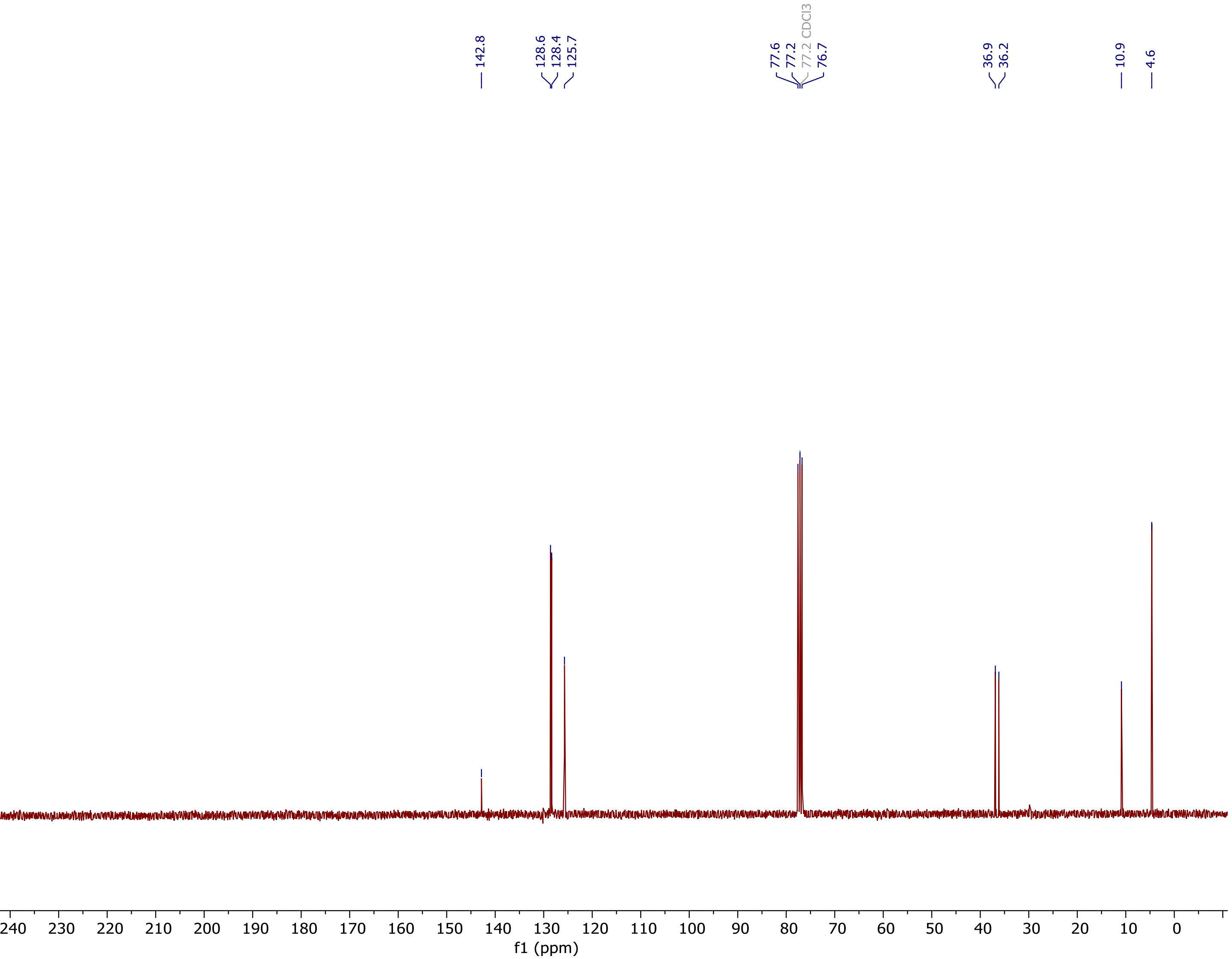

Compound 31 ^{1}H NMR in $CDCl_3$, 298 K, 300 MHz

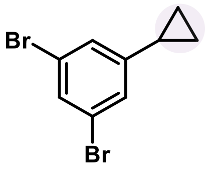

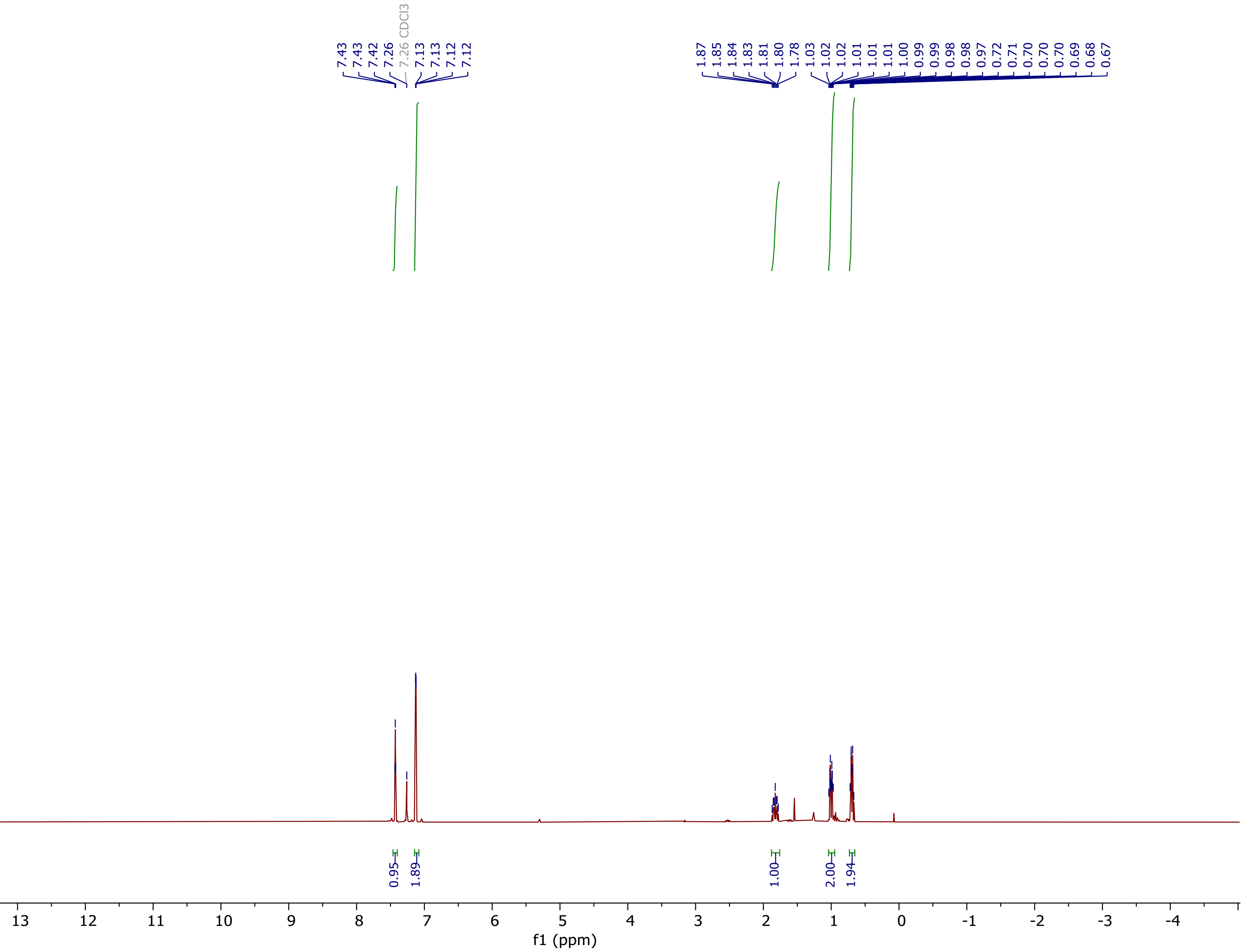

Compound 31 ^{13}C NMR in $CDCl_3$, 298 K, 75 MHz

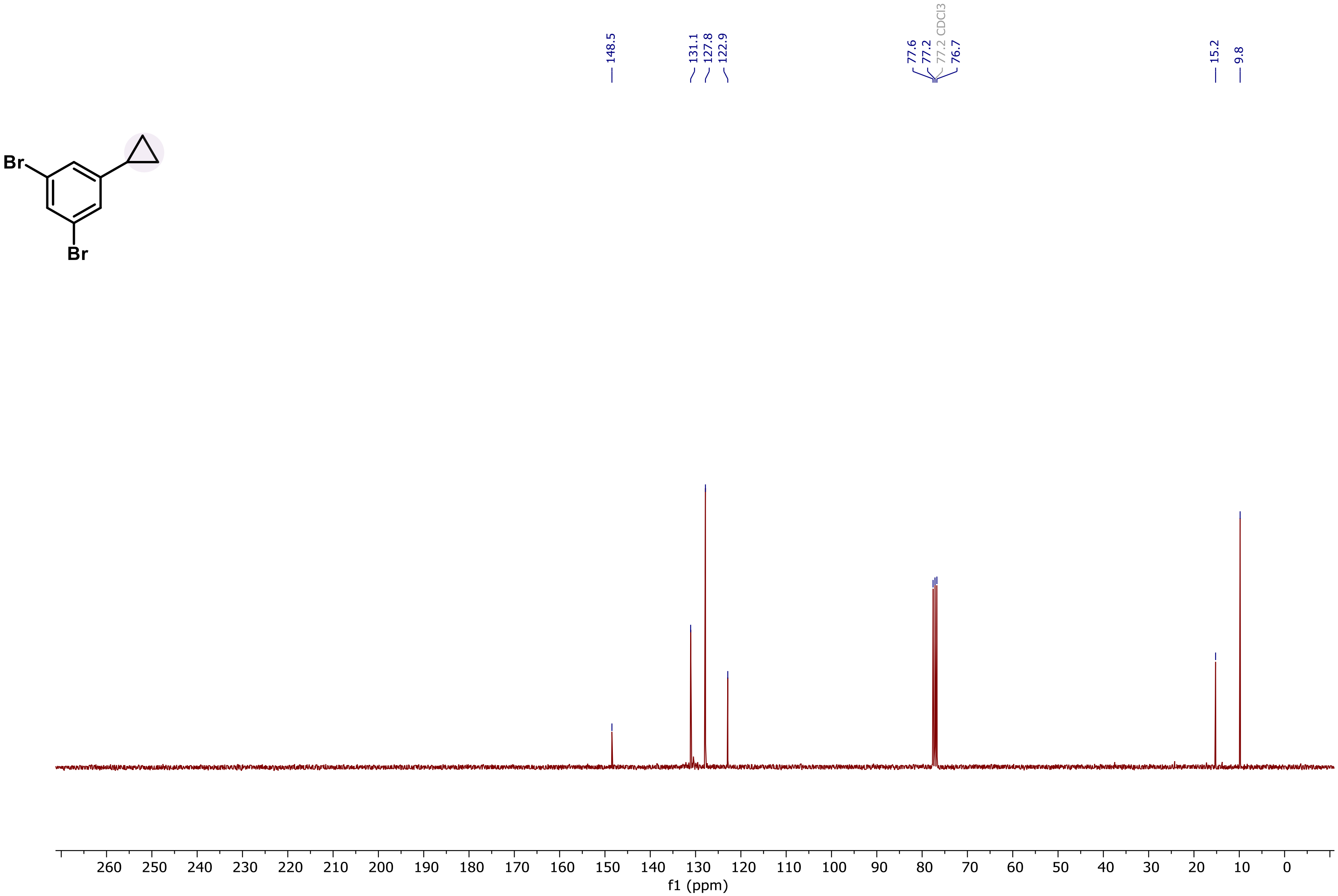

Compound 32 ^{1}H NMR in $CDCl_3$, 298 K, 300 MHz

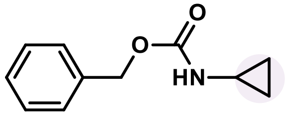

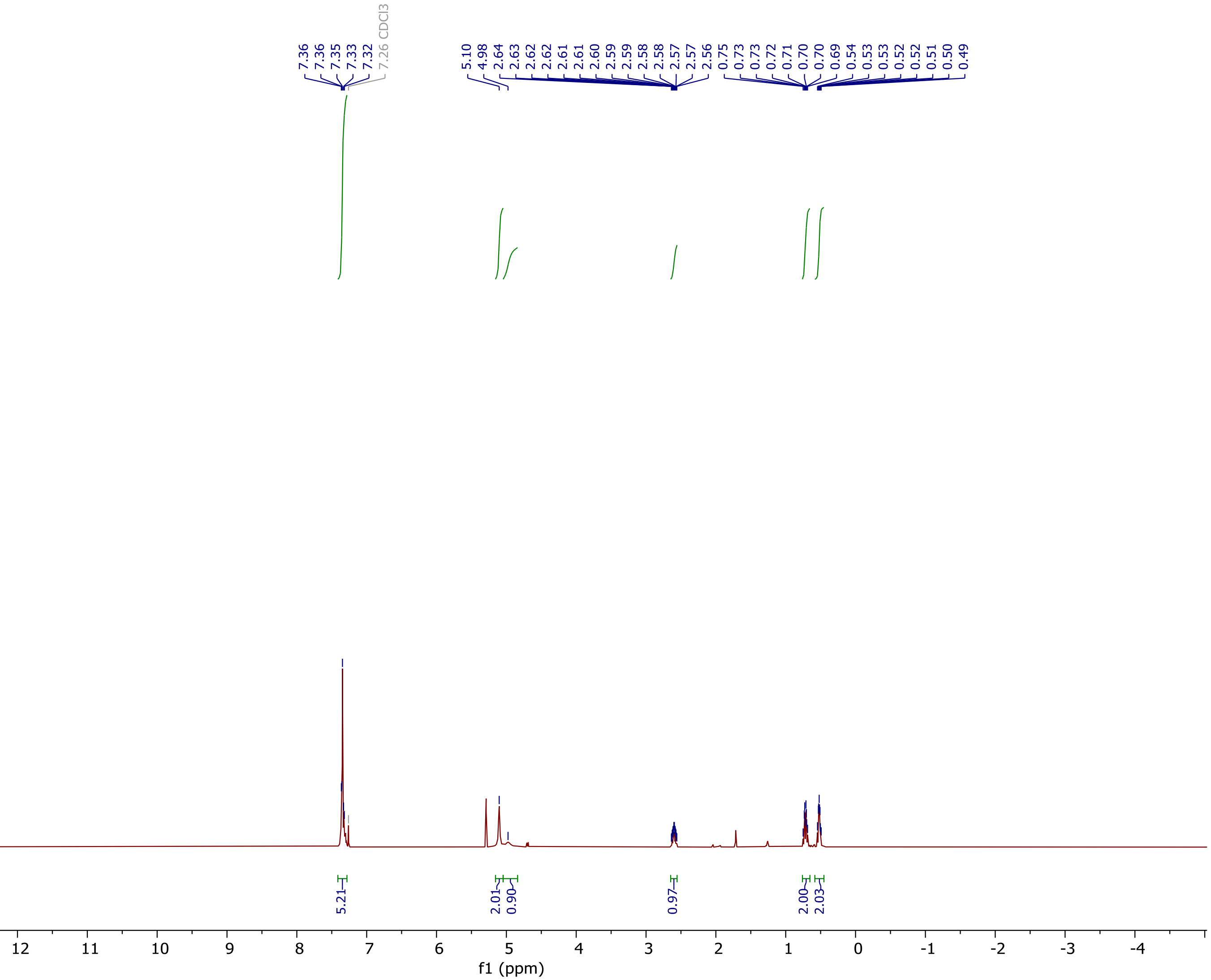

Compound 32 ^{13}C NMR in $CDCl_3$, 298 K, 75 MHz

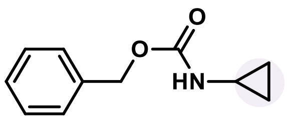

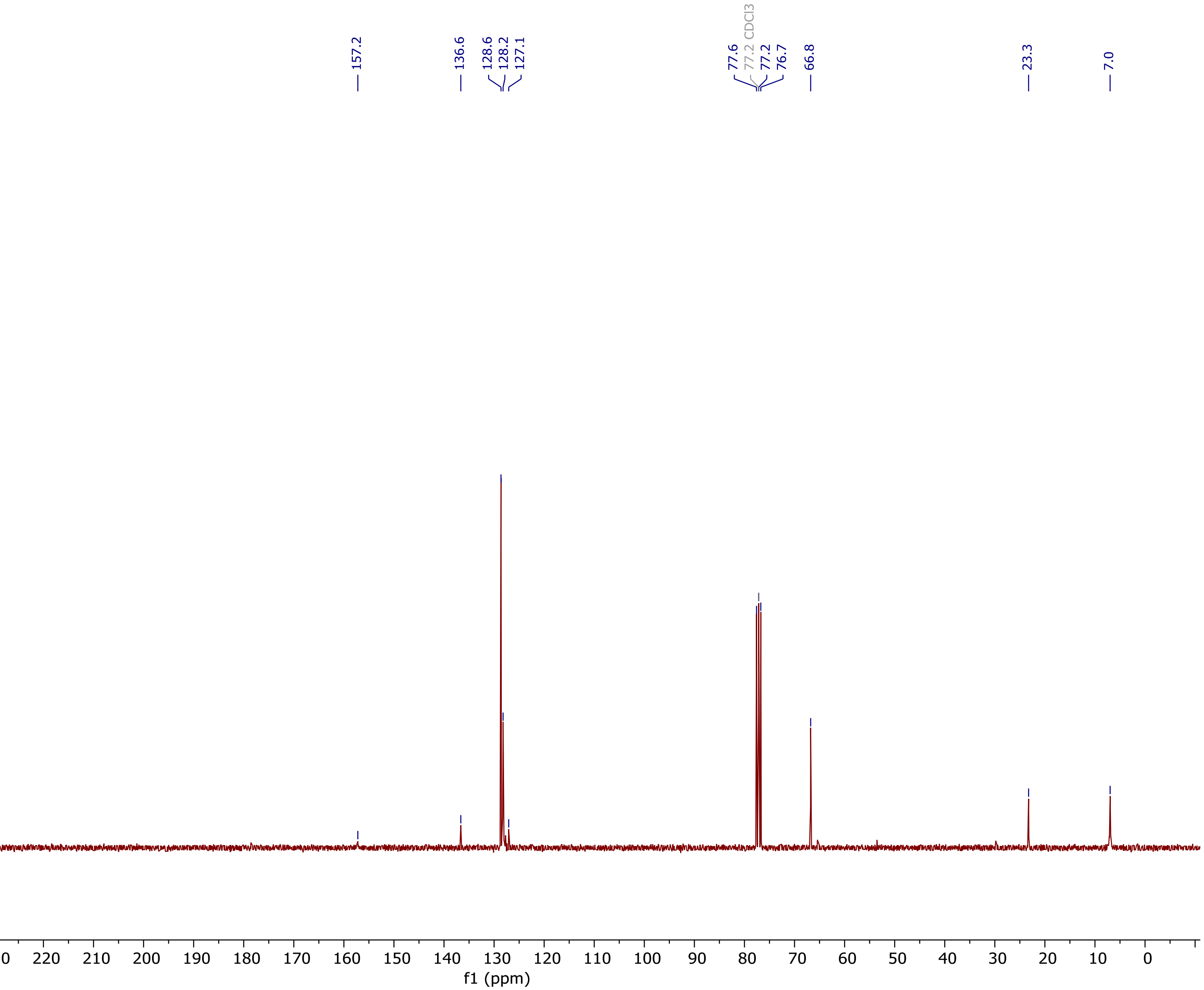

Supplement: Supplementary file 1 — ja4c07262_si_001.pdf [file ja4c07262_si_001.pdf]
